# Supplementary material for: Activity-dependent extracellular proteolytic cascade cleaves the ECM component brevican to promote structural plasticity
Source: EMBO Rep. 2025 Nov 19;27(1):163–85. doi: 10.1038/s44319-025-00644-w (PMC12796228; doi:10.1038/s44319-025-00644-w)
Supplement: Supplementary file 1 — Table EV1 [file 44319_2025_644_MOESM1_ESM.docx]

**Table EV1**

**Statistics: Figure 1D**

**145 kDa**

| **(t)** | **Dunnett's multiple comparisons test** | **Mean1** | **Mean2** | **SEM1** | **SEM2** | **n1** | **n2** | **Adjusted P Value** |
| --- | --- | --- | --- | --- | --- | --- | --- | --- |
| 0 | 0 min vs. PFR 0’ | 1.000 | 1.000 | 0 | 0 | 3 | 3 |  |
| 15 | 0 min vs. PFR 15 | 1.000 | 1.314 | 0 | 0,1581 | 3 | 3 | 0.3231 |
| 45 | 0 min vs. PFR`45’ | 1.000 | 1.285 | 0 | 0,1551 | 3 | 3 | 0.3975 |
| 90 | 0 min vs. PFR 90’ | 1.000 | 1.406 | 0 | 0,1707 | 3 | 3 | 0.1548 |
| 180 | 0 min vs. PFR 180’ | 1.000 | 0.9541 | 0 | 0,09078 | 3 | 3 | 0.9970 |

**53 kDa**

| **(t)** | **Dunnett's multiple comparisons test** | **Mean1** | **Mean2** | **SEM1** | **SEM2** | **n1** | **n2** | **Adjusted P Value** |
| --- | --- | --- | --- | --- | --- | --- | --- | --- |
| 0 | 0 min vs. PFR 0’ | 1.000 | 1.000 | 0 | 0 | 4 | 4 |  |
| 15 | 0 min vs. PFR 15 | 1.000 | 0.1581 | 0 | 0.2396 | 4 | 4 | 0.0099 |
| 45 | 0 min vs. PFR`45’ | 1.000 | 0.1551 | 0 | 0.1820 | 4 | 4 | 0.0304 |
| 90 | 0 min vs. PFR 90’ | 1.000 | 1.531 | 0 | 0.1049 | 4 | 4 | 0.0771 |
| 180 | 0 min vs. PFR 180’ | 1.000 | 1.020 | 0 | 0.1072 | 4 | 4 | >0.9999 |

**Neo**

| **(t)** | **Dunnett's multiple comparisons test** | **Mean1** | **Mean2** | **SEM1** | **SEM2** | **n1** | **n2** | **Adjusted P Value** |
| --- | --- | --- | --- | --- | --- | --- | --- | --- |
| 0 | 0 min vs. PFR 0’ | 1.000 | 1.000 | 0 | 0 | 4 | 4 |  |
| 15 | 0 min vs. PFR 15 | 1.000 | 1,488 | 0 | 0.1441 | 4 | 4 | 0.0029 |
| 45 | 0 min vs. PFR`45’ | 1.000 | 1,432 | 0 | 0.08545 | 4 | 4 | 0.0074 |
| 90 | 0 min vs. PFR 90’ | 1.000 | 1,406 | 0 | 0.04511 | 4 | 4 | 0.0116 |
| 180 | 0 min vs. PFR 180’ | 1.000 | 0,9331 | 0 | 0.06369 | 4 | 4 | 0.9412 |

**Figure 1F**

|  | **PFR** | **PFR+Pic.** | **PFR+TIMP3** | **PFR+GM6001** |
| --- | --- | --- | --- | --- |
| Number of values | 6 | 5 | 8 | 4 |
|  |  |  |  |  |
| Minimum | 1.176 | 0.6840 | 0.6628 | 0.6358 |
| 25% Percentile | 1.220 | 0.7748 | 0.7663 | 0.6629 |
| Median | 1.303 | 1.042 | 1.030 | 0.7595 |
| 75% Percentile | 1.343 | 1.105 | 1.148 | 0.8745 |
| Maximum | 1.370 | 1.130 | 1.188 | 0.9077 |
| Range | 0.1936 | 0.4458 | 0.5257 | 0.2719 |
|  |  |  |  |  |
| Mean | 1.287 | 0.9605 | 0.9788 | 0.7656 |
| Std. Deviation | 0.07257 | 0.1839 | 0.1982 | 0.1119 |
| Std. Error of Mean | 0.02963 | 0.08224 | 0.07008 | 0.05597 |

| **Šídák's multiple comparisons test** | **Mean1** | **Mean2** | **SEM1** | **SEM2** | **n1** | **n2** | **Adjusted P Value** |
| --- | --- | --- | --- | --- | --- | --- | --- |
| Ctl vs. PFR | 1.000 | 1.287 | 0 | 0.02963 | 4 | 6 | 0.04 |
| Ctl vs. PFR+Pic. | 1.000 | 0.9605 | 0 | 0.08224 | 4 | 5 | >0.99 |
| Ctl vs. PFR+Timp3 | 1.000 | 0.9788 | 0 | 0.07008 | 4 | 8 | >0.99 |
| Ctl vs. PFR+GM6001 | 1.000 | 0.7656 | 0 | 0.05597 | 4 | 4 | 0.22 |
| PFR vs. PFR+Pic. | 1.287 | 0.9605 | 0.02963 | 0.08224 | 6 | 5 | 0.009 |
| PFR vs. PFR+Timp3 | 1.287 | 0.9788 | 0.02963 | 0.07008 | 6 | 8 | 0.006 |
| PFR vs. PFR+GM6001 | 1.287 | 0.7656 | 0.02963 | 0.05597 | 6 | 4 | <0.001 |

**Figure 1G**

|  | **PFR** | **PFR+TIMP3** | **PFR+GM6001** | **PFR + Pic.** |
| --- | --- | --- | --- | --- |
| Number of values | 13 | 5 | 8 | 4 |
|  |  |  |  |  |
| Minimum | 1.108 | 1.258 | 1.202 | 1.109 |
| 25% Percentile | 1.140 | 1.290 | 1.264 | 1.131 |
| Median | 1.245 | 1.429 | 1.302 | 1.242 |
| 75% Percentile | 1.288 | 1.552 | 1.420 | 1.375 |
| Maximum | 1.424 | 1.670 | 1.595 | 1.403 |
| Range | 0.3165 | 0.4119 | 0.3929 | 0.2944 |
|  |  |  |  |  |
| Mean | 1.231 | 1.423 | 1.341 | 1.249 |
| Std. Deviation | 0.09739 | 0.1572 | 0.1248 | 0.1265 |
| Std. Error of Mean | 0.02701 | 0.07028 | 0.04412 | 0.06323 |

| **Šídák's multiple comparisons test** | **Mean1** | **Mean2** | **SEM1** | **SEM2** | **n1** | **n2** | **Adjusted P Value** |
| --- | --- | --- | --- | --- | --- | --- | --- |
| Ctl vs. PFR | 1.000 | 1.231 | 0 | 0.02701 | 13 | 13 | <0.001 |
| Ctl vs. PFR+Timp3 | 1.000 | 1.423 | 0 | 0.07028 | 13 | 5 | <0.001 |
| Ctl vs. PFR+GM6001 | 1.000 | 1.341 | 0 | 0.04412 | 13 | 8 | <0.001 |
| Ctl vs. PFR + Pic. | 1.000 | 1.249 | 0 | 0.05597 | 13 | 4 | <0.001 |
| PFR vs. PFR+Timp3 | 1.231 | 1.423 | 0.02701 | 0.07028 | 13 | 5 | 0.005 |
| PFR vs. PFR+GM6001 | 1.231 | 1.341 | 0.02701 | 0.04412 | 13 | 8 | 0.12 |
| PFR vs. PFR + Pic. | 1.231 | 1.249 | 0.02701 | 0.05597 | 13 | 4 | >0.99 |

**Figure 1H**

| **Šídák's multiple comparisons test** | **Mean1** | **Mean2** | **SEM1** | **SEM2** | **n1** | **n2** | **Adjusted P Value** |
| --- | --- | --- | --- | --- | --- | --- | --- |
| Ctl vs. PFR | 1.000 | 1.446 | 0 | 0.01106 | 11 | 9 | <0.001 |
| Ctl vs. PFR + Pic. | 1.000 | 0.9990 | 0 | 0.1216 | 11 | 4 | >0.99 |
| Ctl vs. PFR+Timp3 | 1.000 | 0.9679 | 0 | 0.07309 | 11 | 6 | >0.99 |
| PFR vs. PFR + Pic. | 1.446 | 0.9990 | 0.01106 | 0.1216 | 9 | 4 | 0.008 |
| PFR vs. PFR+Timp3 | 1.446 | 0.9679 | 0.01106 | 0.07309 | 9 | 6 | <0.001 |
| PFR vs. PFR+GM6001 | 1.446 | 0.7954 | 0.01106 | 0.0245 | 9 | 4 | <0.001 |
| Ctl vs. PFR+GM6001 | 1.000 | 0.7954 | 0 | 0.0245 | 11 | 4 | 0.52 |

|  | **PFR** | **PFR + Pic.** | **PFR+TIMP3** | **PFR+GM6001** |
| --- | --- | --- | --- | --- |
| Number of values | 9 | 4 | 6 | 4 |
|  |  |  |  |  |
| Minimum | 1.118 | 0.7404 | 0.7645 | 0.7564 |
| 25% Percentile | 1.200 | 0.7878 | 0.8090 | 0.7600 |
| Median | 1.410 | 0.9655 | 0.9641 | 0.7795 |
| 75% Percentile | 1.585 | 1.244 | 1.143 | 0.8468 |
| Maximum | 2.196 | 1.324 | 1.150 | 0.8663 |
| Range | 1.078 | 0.5839 | 0.3854 | 0.1099 |
|  |  |  |  |  |
| Mean | 1.446 | 0.9990 | 0,9679 | 0,7954 |
| Std. Deviation | 0.3318 | 0.2432 | 0,1790 | 0,04900 |
| Std. Error of Mean | 0.1106 | 0.1216 | 0,07309 | 0,02450 |

**Figure 1K**

|  | **PFR** | **PFR+TIMP3** |
| --- | --- | --- |
| Number of values | 7 | 7 |
|  |  |  |
| Minimum | 1.151 | 0.7248 |
| 25% Percentile | 1.183 | 0.8072 |
| Median | 1.213 | 0.9398 |
| 75% Percentile | 1.378 | 1.226 |
| Maximum | 1.452 | 1.298 |
| Range | 0.3013 | 0.5732 |
|  |  |  |
| Mean | 1.261 | 1.005 |
| Std. Deviation | 0.1109 | 0.2301 |
| Std. Error of Mean | 0.04192 | 0.08695 |

| **Šídák's multiple comparisons test** | **Mean1** | **Mean2** | **SEM1** | **SEM2** | **n1** | **n2** | **Adjusted P Value** |
| --- | --- | --- | --- | --- | --- | --- | --- |
| Ctl vs. PFR | 1.000 | 1.261 | 0 | 0.04192 | 7 | 7 | 0.01 |
| Ctl vs. PFR+Timp3 | 1.000 | 1.005 | 0 | 0.08695 | 7 | 7 | >0.99 |
| PFR vs. PFR+Timp3 | 1.261 | 1.005 | 0.04192 | 0.08695 | 7 | 7 | 0.01 |
