# Supplementary material for: Activity-dependent extracellular proteolytic cascade cleaves the ECM component brevican to promote structural plasticity
Source: EMBO Rep. 2025 Nov 19;27(1):163–85. doi: 10.1038/s44319-025-00644-w (PMC12796228; doi:10.1038/s44319-025-00644-w)
Supplement: Supplementary file 2 — Table EV2 [file 44319_2025_644_MOESM2_ESM.docx]

**Table EV2**

**Figure 2B**

53 kDa

|  | **PFR** | **PFR+Furini-II** | **PFR+PCi** | **PFR+Furini-I** |
| --- | --- | --- | --- | --- |
| Number of values | 21 | 4 | 7 | 10 |
|  |  |  |  |  |
| Minimum | 1.021 | 0.6772 | 0.9285 | 0.8120 |
| 25% Percentile | 1.144 | 0.7284 | 0.9712 | 0.9546 |
| Median | 1.217 | 0.9671 | 1.081 | 1.051 |
| 75% Percentile | 1.516 | 1.077 | 1.107 | 1.316 |
| Maximum | 2.196 | 1.086 | 1.148 | 1.354 |
| Range | 1.175 | 0.4086 | 0.2191 | 0.5422 |
|  |  |  |  |  |
| Mean | 1.358 | 0.9243 | 1.056 | 1.087 |
| Std. Deviation | 0.3151 | 0.1873 | 0.07833 | 0.1853 |
| Std. Error of Mean | 0.06875 | 0.09366 | 0.02961 | 0.05860 |

| **Šídák's multiple comparisons test** | **Mean1** | **Mean2** | **SEM1** | **SEM2** | **n1** | **n2** | **Adjusted P Value** |
| --- | --- | --- | --- | --- | --- | --- | --- |
| Ctl vs. PFR | 1.000 | 1.358 | 0 | 0.06875 | 18 | 21 | <0.001 |
| Ctl vs. PFR+Furini-II | 1.000 | 0.9243 | 0 | 0.09366 | 18 | 4 | >0.99 |
| Ctl vs. PFR+PCi | 1.000 | 1.056 | 0 | 0.02961 | 18 | 7 | >0.99 |
| Ctl vs. PFR+Furini-I | 1.000 | 1.087 | 0 | 0.05860 | 18 | 10 | 0.92 |
| PFR vs. PFR+Furini-II | 1.358 | 0.9243 | 0.06875 | 0.09366 | 21 | 4 | 0.003 |
| PFR vs. PFR+PCi | 1.358 | 1.056 | 0.06875 | 0.02961 | 21 | 7 | 0.01 |
| PFR vs. PFR+Furini-I | 1.358 | 1.087 | 0.06875 | 0.05860 | 21 | 10 | 0.01 |

145 kDa

|  | **PFR** | **PFR+Furini-II** | **PFR+Furini-I** | **PFR+PCi** |
| --- | --- | --- | --- | --- |
| Number of values | 15 | 6 | 10 | 6 |
|  |  |  |  |  |
| Minimum | 1.078 | 1.059 | 1.149 | 1.160 |
| 25% Percentile | 1.094 | 1.173 | 1.174 | 1.211 |
| Median | 1.253 | 1.412 | 1.409 | 1.295 |
| 75% Percentile | 1.468 | 1.560 | 1.607 | 1.415 |
| Maximum | 1.702 | 1.605 | 1.752 | 1.459 |
| Range | 0.6247 | 0.5457 | 0.6025 | 0.2985 |
|  |  |  |  |  |
| Mean | 1.305 | 1.374 | 1.416 | 1.306 |
| Std. Deviation | 0.2147 | 0.2083 | 0.2202 | 0.1099 |
| Std. Error of Mean | 0.05544 | 0.08504 | 0.06963 | 0.04489 |

| **Šídák's multiple comparisons test** | **Mean1** | **Mean2** | **SEM1** | **SEM2** | **n1** | **n2** | **Adjusted P Value** |
| --- | --- | --- | --- | --- | --- | --- | --- |
| Ctl vs. PFR | 1.000 | 1.305 | 0 | 0.05544 | 16 | 15 | <0.001 |
| Ctl vs. PFR+Furini-II | 1.000 | 1.374 | 0 | 0.08504 | 16 | 6 | <0.001 |
| Ctl vs. PFR+PCi | 1.000 | 1.306 | 0 | 0.04489 | 16 | 6 | 0.004 |
| Ctl vs. PFR+Furini-I | 1.000 | 1.416 | 0 | 0.06963 | 16 | 10 | <0.001 |
| PFR vs. PFR+Furini-II | 1.305 | 1.374 | 0.05544 | 0.08504 | 15 | 6 | >0.99 |
| PFR vs. PFR+PCi | 1.305 | 1.306 | 0.05544 | 0.04489 | 15 | 6 | >0.99 |
| PFR vs. PFR+Furini-I | 1.305 | 1.416 | 0.05544 | 0.06963 | 15 | 10 | 0.71 |

Neo:

|  | **PFR** | **PFR+Furini-I** | **PFR+Furini- II** | **PFR+PCi** |
| --- | --- | --- | --- | --- |
| Number of values | 14 | 9 | 8 | 7 |
|  |  |  |  |  |
| Minimum | 1.115 | 0.9556 | 0.8495 | 1.002 |
| 25% Percentile | 1.226 | 0.9859 | 0.9490 | 1.053 |
| Median | 1.335 | 1.133 | 1.079 | 1.094 |
| 75% Percentile | 1.632 | 1.363 | 1.274 | 1.242 |
| Maximum | 1.755 | 1.580 | 1.471 | 1.296 |
| Range | 0.6401 | 0.6248 | 0.6216 | 0.2947 |
|  |  |  |  |  |
| Mean | 1.404 | 1.172 | 1.104 | 1.132 |
| Std. Deviation | 0.2290 | 0.2367 | 0.2068 | 0.1063 |
| Std. Error of Mean | 0.06120 | 0.07891 | 0.07312 | 0.04019 |

| **Šídák's multiple comparisons test** | **Mean1** | **Mean2** | **SEM1** | **SEM2** | **n1** | **n2** | **Adjusted P Value** |
| --- | --- | --- | --- | --- | --- | --- | --- |
| Ctl vs. PFR | 1.000 | 1.404 | 0 | 0.0612 | 15 | 14 | <0.001 |
| Ctl vs. PFR+Furini- II | 1.000 | 1.104 | 0 | 0.07312 | 15 | 8 | 0.76 |
| Ctl vs. PFR+Furini-I | 1.000 | 1.136 | 0 | 0.07891 | 15 | 9 | 0.16 |
| Ctl vs. PFR+PCi | 1.000 | 1.132 | 0 | 0.04019 | 15 | 7 | 0.56 |
| PFR vs. PFR+Furini- II | 1.404 | 1.104 | 0.0612 | 0.07312 | 14 | 8 | 0.003 |
| PFR vs. PFR+Furini-I | 1.404 | 1.136 | 0.0612 | 0.07891 | 14 | 9 | 0.02 |
| PFR vs. PFR+PCi | 1.404 | 1.132 | 0.0612 | 0.04019 | 14 | 7 | 0.01 |

**Figure 2C**

|  | **APMA** | **APMA+PFR** |
| --- | --- | --- |
| Number of values | 4 | 3 |
|  |  |  |
| Minimum | 1.156 | 1.294 |
| 25% Percentile | 1.197 | 1.294 |
| Median | 1.381 | 1.451 |
| 75% Percentile | 1.473 | 1.530 |
| Maximum | 1.483 | 1.530 |
| Range | 0.3272 | 0.2359 |
|  |  |  |
| Mean | 1.350 | 1.425 |
| Std. Deviation | 0.1470 | 0.1201 |
| Std. Error of Mean | 0.07349 | 0.06932 |

| **Dunnett's multiple comparisons test** | **Mean1** | **Mean2** | **SEM1** | **SEM2** | **n1** | **n2** | **Adjusted P Value** |
| --- | --- | --- | --- | --- | --- | --- | --- |
| Ctl vs. APMA | 1.000 | 1.350 | 0 | 0.07349 | 4 | 4 | 0.0404 |
| Ctl vs. APMA+PFR | 1.000 | 1.425 | 0 | 0.06932 | 4 | 3 | 0.0220 |
