# Supplementary material for: Activity-dependent extracellular proteolytic cascade cleaves the ECM component brevican to promote structural plasticity
Source: EMBO Rep. 2025 Nov 19;27(1):163–85. doi: 10.1038/s44319-025-00644-w (PMC12796228; doi:10.1038/s44319-025-00644-w)
Supplement: Supplementary file 3 — Table EV3 [file 44319_2025_644_MOESM3_ESM.docx]

**Table EV3**

**Figure 3B**

|  | **PFR** | **PFR+CBX** | **PFR+Endo** |
| --- | --- | --- | --- |
| Number of values | 7 | 6 | 7 |
|  |  |  |  |
| Minimum | 1.072 | 0.7986 | 0.7170 |
| 25% Percentile | 1.098 | 0.8812 | 0.8296 |
| Median | 1.185 | 0.9293 | 0.9969 |
| 75% Percentile | 1.207 | 0.9899 | 1.068 |
| Maximum | 1.218 | 1.009 | 1.150 |
| Range | 0.1462 | 0.2106 | 0.4331 |
|  |  |  |  |
| Mean | 1.165 | 0.9264 | 0.9517 |
| Std. Deviation | 0.05650 | 0.07346 | 0.1474 |
| Std. Error of Mean | 0.02135 | 0.02999 | 0.05570 |

| **Šídák's multiple comparisons test** | **Mean1** | **Mean2** | **SEM1** | **SEM2** | **n1** | **n2** | **Adjusted P Value** |
| --- | --- | --- | --- | --- | --- | --- | --- |
| Ctl vs. PFR | 1.000 | 1.165 | 0 | 0.02135 | 8 | 7 | 0.0055 |
| Ctl vs. PFR+CBX | 1.000 | 0.9264 | 0 | 0.02999 | 8 | 6 | 0.4879 |
| Ctl vs. PFR+Endo | 1.000 | 0.9517 | 0 | 0.05570 | 8 | 7 | 0.8164 |
| PFR vs. PFR+CBX | 1.165 | 0.9264 | 0.02135 | 0.02999 | 7 | 6 | 0.0002 |
| PFR vs. PFR+Endo | 1.165 | 0.9517 | 0.02135 | 0.05570 | 7 | 7 | 0.0005 |

**Figure 3D**

|  | **D-Ser** | **D-Ser+PFR** | **D-Ser+PFR+CBX** | **D-Ser+PFR+D-APV** |
| --- | --- | --- | --- | --- |
| Number of values | 7 | 9 | 9 | 6 |
|  |  |  |  |  |
| Minimum | 1.071 | 1.064 | 0.8812 | 0.7935 |
| 25% Percentile | 1.134 | 1.281 | 1.147 | 0.8435 |
| Median | 1.347 | 1.333 | 1.304 | 0.9848 |
| 75% Percentile | 1.398 | 1.617 | 1.452 | 1.082 |
| Maximum | 1.404 | 1.912 | 1.729 | 1.180 |
| Range | 0.3332 | 0.8484 | 0.8480 | 0.3868 |
|  |  |  |  |  |
| Mean | 1.282 | 1.439 | 1.291 | 0.9754 |
| Std. Deviation | 0.1325 | 0.2574 | 0.2480 | 0.1398 |
| Std. Error of Mean | 0.05007 | 0.08579 | 0.08267 | 0.05709 |

| **Šídák's multiple comparisons test** | **Mean1** | **Mean2** | **SEM1** | **SEM2** | **n1** | **n2** | **Adjusted P Value** |
| --- | --- | --- | --- | --- | --- | --- | --- |
| Ctl vs. D-Ser | 1.000 | 1.282 | 0 | 0.05007 | 10 | 7 | 0.03 |
| Ctl vs. D-Ser+PFR | 1.000 | 1.439 | 0 | 0.08579 | 10 | 9 | <0.001 |
| Ctl vs. D-Ser+PFR+CBX | 1.000 | 1.291 | 0 | 0.08267 | 10 | 9 | 0.01 |
| Ctl vs. D-Ser+PFR+D-APV | 1.000 | 0.9754 | 0 | 0.05709 | 10 | 6 | >0.99 |
| D-Ser vs. D-Ser+PFR | 1.282 | 1.439 | 0.05007 | 0.08579 | 7 | 9 | 0.52 |
| D-Ser vs. D-Ser+PFR+CBX | 1.282 | 1.291 | 0.05007 | 0.08267 | 7 | 9 | >0.99 |
| D-Ser vs. D-Ser+PFR+D-APV | 1.282 | 0.9754 | 0.05007 | 0.05709 | 7 | 6 | 0.03 |
