# Supplementary material for: Activity-dependent extracellular proteolytic cascade cleaves the ECM component brevican to promote structural plasticity
Source: EMBO Rep. 2025 Nov 19;27(1):163–85. doi: 10.1038/s44319-025-00644-w (PMC12796228; doi:10.1038/s44319-025-00644-w)
Supplement: Supplementary file 4 — Table EV4 [file 44319_2025_644_MOESM4_ESM.docx]

**Table EV4**

**Figure 4B**

145 kDa

|  | **PFR** | **PFR+CNQX** | **PFR+MK801** | **PFR+Nifedipine** | **PFR+RO** |
| --- | --- | --- | --- | --- | --- |
| Number of values | 15 | 5 | 4 | 4 | 4 |
|  |  |  |  |  |  |
| Minimum | 1.094 | 0.5765 | 1.039 | 0.8812 | 1.244 |
| 25% Percentile | 1.167 | 0.6749 | 1.063 | 0.9216 | 1.286 |
| Median | 1.354 | 0.8254 | 1.312 | 1.105 | 1.465 |
| 75% Percentile | 1.577 | 1.151 | 1.555 | 1.181 | 1.704 |
| Maximum | 1.885 | 1.221 | 1.578 | 1.185 | 1.765 |
| Range | 0.7907 | 0.6447 | 0.5393 | 0.3040 | 0.5205 |
|  |  |  |  |  |  |
| Mean | 1.391 | 0.8953 | 1.310 | 1.069 | 1.485 |
| Std. Deviation | 0.2464 | 0.2558 | 0.2626 | 0.1403 | 0.2185 |
| Std. Error of Mean | 0.06361 | 0.1144 | 0.1313 | 0.07014 | 0.1092 |

| **Šídák's multiple comparisons test** | **Mean1** | **Mean2** | **SEM1** | **SEM2** | **n1** | **n2** | **Adjusted P Value** |
| --- | --- | --- | --- | --- | --- | --- | --- |
| Ctl vs. PFR | 1.000 | 1.391 | 0 | 0.06361 | 16 | 15 | <0.001 |
| Ctl vs. PFR+CNQX | 1.000 | 0.8953 | 0 | 0.1144 | 16 | 5 | 0.95 |
| Ctl vs. PFR+MK801 | 1.000 | 1.310 | 0 | 0.1313 | 16 | 4 | 0.05 |
| Ctl vs. PFR+Nifedipine | 1.000 | 1.069 | 0 | 0.07014 | 16 | 4 | >0.99 |
| Ctl vs. PFR+RO | 1.000 | 1.485 | 0 | 0.1092 | 16 | 4 | <0.001 |
| PFR vs. PFR+CNQX | 1.391 | 0.8953 | 0.06361 | 0.1144 | 15 | 5 | <0.001 |
| PFR vs. PFR+MK801 | 1.391 | 1.310 | 0.06361 | 0.1313 | 15 | 4 | >0.99 |
| PFR vs. PFR+Nifedipine | 1.391 | 1.069 | 0.06361 | 0.07014 | 15 | 4 | 0.04 |
| PFR vs. PFR+RO | 1.391 | 1.485 | 0.06361 | 0.1092 | 15 | 4 | 0.99 |

**Figure 4C**

Neo

|  | **PFR** | **PFR+CNQX** | **PFR+MK801** | **PFR+Nifedipine** | **PFR+RO** |
| --- | --- | --- | --- | --- | --- |
| Number of values | 9 | 3 | 4 | 4 | 4 |
|  |  |  |  |  |  |
| Minimum | 1.147 | 0.5528 | 0.7447 | 0.7853 | 0.7193 |
| 25% Percentile | 1.273 | 0.5528 | 0.7818 | 0.8067 | 0.7531 |
| Median | 1.410 | 0.7627 | 1.015 | 0.9145 | 0.9240 |
| 75% Percentile | 1.607 | 1.023 | 1.139 | 1.240 | 1.078 |
| Maximum | 1.753 | 1.023 | 1.140 | 1.334 | 1.106 |
| Range | 0.6060 | 0.4702 | 0.3950 | 0.5489 | 0.3864 |
|  |  |  |  |  |  |
| Mean | 1.435 | 0.7795 | 0.9787 | 0.9871 | 0.9182 |
| Std. Deviation | 0.2021 | 0.2355 | 0.1942 | 0.2419 | 0.1678 |
| Std. Error of Mean | 0.06738 | 0.1360 | 0.09710 | 0.1209 | 0.08391 |

| **Šídák's multiple comparisons test** | **Mean1** | **Mean2** | **SEM1** | **SEM2** | **n1** | **n2** | **Adjusted P Value** |
| --- | --- | --- | --- | --- | --- | --- | --- |
| Ctl vs. PFR | 1.000 | 1.435 | 0 | 0.06738 | 6 | 9 | 0.001 |
| Ctl vs. PFR+CNQX | 1.000 | 0.7795 | 0 | 0.1360 | 6 | 3 | 0.62 |
| Ctl vs. PFR+MK801 | 1.000 | 0.9787 | 0 | 0.09710 | 6 | 4 | >0.99 |
| Ctl vs. PFR+Nifedipine | 1.000 | 0.9871 | 0 | 0.1209 | 6 | 4 | >0.99 |
| Ctl vs. PFR+RO | 1.000 | 0.9182 | 0 | 0.08391 | 6 | 4 | >0.99 |
| PFR vs. PFR+CNQX | 1.435 | 0.7795 | 0.06738 | 0.1360 | 9 | 3 | <0.001 |
| PFR vs. PFR+MK801 | 1.435 | 0.9787 | 0.06738 | 0.09710 | 9 | 4 | 0.003 |
| PFR vs. PFR+Nifedipine | 1.435 | 0.9871 | 0.06738 | 0.1209 | 9 | 4 | 0.004 |
| PFR vs. PFR+RO | 1.435 | 0.9182 | 0.06738 | 0.08391 | 9 | 4 | <0.001 |

**Figure 4E**

145 kDa

|  | **PFR** | **AIP** | **PFR+AIP** |
| --- | --- | --- | --- |
| Number of values | 7 | 5 | 8 |
|  |  |  |  |
| Minimum | 1.042 | 0.6732 | 0.6844 |
| 25% Percentile | 1.111 | 0.7738 | 0.7999 |
| Median | 1.198 | 1.008 | 0.9525 |
| 75% Percentile | 1.428 | 1.116 | 1.026 |
| Maximum | 1.566 | 1.131 | 1.056 |
| Range | 0.5240 | 0.4582 | 0.3714 |
|  |  |  |  |
| Mean | 1.260 | 0.9576 | 0.9219 |
| Std. Deviation | 0.1851 | 0.1878 | 0.1333 |
| Std. Error of Mean | 0.06996 | 0.08397 | 0.04711 |

| **Šídák's multiple comparisons test** | **Mean1** | **Mean2** | **SEM1** | **SEM2** | **n1** | **n2** | **Adjusted P Value** |
| --- | --- | --- | --- | --- | --- | --- | --- |
| Ctl vs. PFR | 1.000 | 1.260 | 0 | 0.06996 | 8 | 7 | 0.007 |
| Ctl vs. AIP | 1.000 | 0.9576 | 0 | 0.08397 | 8 | 5 | 0.99 |
| Ctl vs. PFR+AIP | 1.000 | 0.9219 | 0 | 0.04711 | 4 | 8 | 0.80 |
| PFR vs. AIP | 1.260 | 0.9576 | 0.06996 | 0.08397 | 7 | 5 | 0.006 |
| PFR vs. PFR+AIP | 1.260 | 0.9219 | 0.06996 | 0.04711 | 7 | 8 | <0.001 |

**Figure 4F**

Neo

|  | **PFR** | **AIP** | **PFR+AIP** |
| --- | --- | --- | --- |
| Number of values | 5 | 5 | 8 |
|  |  |  |  |
| Minimum | 1.126 | 0.7030 | 0.7190 |
| 25% Percentile | 1.172 | 0.7086 | 0.7536 |
| Median | 1.247 | 1.100 | 0.9016 |
| 75% Percentile | 1.538 | 1.214 | 1.075 |
| Maximum | 1.732 | 1.264 | 1.234 |
| Range | 0.6057 | 0.5614 | 0.5147 |
|  |  |  |  |
| Mean | 1.333 | 0.9890 | 0.9322 |
| Std. Deviation | 0.2361 | 0.2626 | 0.1798 |
| Std. Error of Mean | 0.1056 | 0.1174 | 0.06357 |

| **Šídák's multiple comparisons test** | **Mean1** | **Mean2** | **SEM1** | **SEM2** | **n1** | **n2** | **Adjusted P Value** |
| --- | --- | --- | --- | --- | --- | --- | --- |
| Ctl vs. PFR | 1.000 | 1.333 | 0 | 0.1056 | 8 | 5 | 0.02 |
| Ctl vs. AIP | 1.000 | 0.9890 | 0 | 0.1174 | 8 | 5 | >0.99 |
| Ctl vs. PFR+AIP | 1.000 | 0.9322 | 0 | 0.06357 | 8 | 8 | 0.96 |
| PFR vs. AIP | 1.333 | 0.9890 | 0.1056 | 0.1174 | 5 | 5 | 0.03 |
| PFR vs. PFR+AIP | 1.333 | 0.9322 | 0.1056 | 0.06357 | 5 | 8 | 0.004 |
