# Supplementary material for: Activity-dependent extracellular proteolytic cascade cleaves the ECM component brevican to promote structural plasticity
Source: EMBO Rep. 2025 Nov 19;27(1):163–85. doi: 10.1038/s44319-025-00644-w (PMC12796228; doi:10.1038/s44319-025-00644-w)
Supplement: Supplementary file 5 — Table EV5 [file 44319_2025_644_MOESM5_ESM.docx]

**Table EV5**

**Figure 5B**

pERK/ERK

|  | **PFR** | **PFR+TIMP3** |
| --- | --- | --- |
| Number of values | 4 | 4 |
|  |  |  |
| Minimum | 1.330 | 1.264 |
| 25% Percentile | 1.330 | 1.345 |
| Median | 1.378 | 1.615 |
| 75% Percentile | 1.451 | 1.667 |
| Maximum | 1.459 | 1.675 |
| Range | 0.1288 | 0.4113 |
|  |  |  |
| Mean | 1.386 | 1.543 |
| Std. Deviation | 0.06596 | 0.1890 |
| Std. Error of Mean | 0.03298 | 0.09451 |

| **Šídák's multiple comparisons test** | **Mean1** | **Mean2** | **SEM1** | **SEM2** | **n1** | **n2** | **Adjusted P Value** |
| --- | --- | --- | --- | --- | --- | --- | --- |
| Ctl vs. PFR | 1.000 | 1.386 | 0 | 0.03298 | 3 | 4 | 0.010 |
| Ctl vs. PFR+Timp3 | 1.000 | 1.543 | 0 | 0.09451 | 3 | 4 | 0.001 |
| PFR vs. PFR+Timp3 | 1.386 | 1.543 | 0.03298 | 0.1731 | 4 | 4 | 0.29 |

**Figure 5C**

pCaMKII/CaMKII

|  | **PFR** | **PFR+TIMP3** |
| --- | --- | --- |
| Number of values | 7 | 7 |
|  |  |  |
| Minimum | 1.055 | 1.081 |
| 25% Percentile | 1.068 | 1.156 |
| Median | 1.114 | 1.208 |
| 75% Percentile | 1.503 | 1.335 |
| Maximum | 1.591 | 1.532 |
| Range | 0.5364 | 0.4511 |
|  |  |  |
| Mean | 1.234 | 1.247 |
| Std. Deviation | 0.2221 | 0.1470 |
| Std. Error of Mean | 0.08395 | 0.05556 |

| **Šídák's multiple comparisons test** | **Mean1** | **Mean2** | **SEM1** | **SEM2** | **n1** | **n2** | **Adjusted P Value** |
| --- | --- | --- | --- | --- | --- | --- | --- |
| Ctl vs. PFR | 1.000 | 1.234 | 0 | 0.08395 | 6 | 7 | 0.0492 |
| Ctl vs. PFR+Timp3 | 1.000 | 1.247 | 0 | 0.05556 | 6 | 7 | 0.0363 |
| PFR vs. PFR+Timp3 | 1.234 | 1.247 | 0.08395 | 0.05556 | 7 | 7 | 0.9983 |

**Figure 5E**

| **Šídák's multiple comparisons test** | **Mean1** | **Mean2** | **SEM1** | **SEM2** | **n1** | **n2** | **Two-tailed P Value** |
| --- | --- | --- | --- | --- | --- | --- | --- |
| Veh vs. Timp3 | 160.692 | 182.135 | 9.803 | 14.816 | 10 | 7 | 0.227 |
