# Supplementary material for: Activity-dependent extracellular proteolytic cascade cleaves the ECM component brevican to promote structural plasticity
Source: EMBO Rep. 2025 Nov 19;27(1):163–85. doi: 10.1038/s44319-025-00644-w (PMC12796228; doi:10.1038/s44319-025-00644-w)
Supplement: Supplementary file 6 — Table EV6 [file 44319_2025_644_MOESM6_ESM.docx]

**Table EV6**

**Figure 6H**

|  | **Ctl** | **PFR** |
| --- | --- | --- |
| Number of values | 71 | 73 |
|  |  |  |
| Minimum | 0.1170 | 0.8423 |
| 25% Percentile | 0.7687 | 1.034 |
| Median | 1.073 | 1.195 |
| 75% Percentile | 1.216 | 1.464 |
| Maximum | 1.640 | 3.152 |
| Range | 1.523 | 2.310 |
|  |  |  |
| Mean | 1.001 | 1.376 |
| Std. Deviation | 0.3138 | 0.5104 |
| Std. Error of Mean | 0.03724 | 0.05974 |

| **Unpaired t test** | **Mean1** | **Mean2** | **SEM1** | **SEM2** | **n1** | **n2** | **Two-tailed P Value** |
| --- | --- | --- | --- | --- | --- | --- | --- |
| Ctl vs. PFR | 1.001 | 1.201 | 0.03724 | 0.03061 | 71 | 63 | <0.0001 |

**Figure 6K**

|  | **Ctl** | **PFR** |
| --- | --- | --- |
| Number of values | 16 | 16 |
|  |  |  |
| Minimum | 0.7743 | 0.9292 |
| 25% Percentile | 0.9444 | 1.057 |
| Median | 1.016 | 1.128 |
| 75% Percentile | 1.115 | 1.285 |
| Maximum | 1.189 | 1.527 |
| Range | 0.4152 | 0.5980 |
|  |  |  |
| Mean | 1.013 | 1.166 |
| Std. Deviation | 0.1173 | 0.1650 |
| Std. Error of Mean | 0.02932 | 0.04124 |

| **Unpaired t test** | **Mean1** | **Mean2** | **SEM1** | **SEM2** | **n1** | **n2** | **Two-tailed P Value** |
| --- | --- | --- | --- | --- | --- | --- | --- |
| Ctl vs. PFR | 1.013 | 1.166 | 0.02932 | 0.04124 | 16 | 16 | 0.005 |
