# Supplementary material for: Activity-dependent extracellular proteolytic cascade cleaves the ECM component brevican to promote structural plasticity
Source: EMBO Rep. 2025 Nov 19;27(1):163–85. doi: 10.1038/s44319-025-00644-w (PMC12796228; doi:10.1038/s44319-025-00644-w)
Supplement: Supplementary file 7 — Table EV7 [file 44319_2025_644_MOESM7_ESM.docx]

**Table EV7**

**Figure 7F**

|  | **Ctl** | **PFR** | **PFR+TIMP3** |
| --- | --- | --- | --- |
| Number of values | 462 | 1140 | 675 |
|  |  |  |  |
| Minimum | 0.01492 | 0.02271 | 0.02449 |
| 25% Percentile | 0.2957 | 0.3953 | 0.2996 |
| Median | 0.6326 | 0.7944 | 0.5537 |
| 75% Percentile | 1.060 | 1.387 | 1.059 |
| Maximum | 2.973 | 3.000 | 2.992 |
| Range | 2.958 | 2.977 | 2.968 |
|  |  |  |  |
| Mean | 0.7600 | 0.9797 | 0.7707 |
| Std. Deviation | 0.5698 | 0.7240 | 0.6367 |
| Std. Error of Mean | 0.02651 | 0.02144 | 0.02451 |

| **Šídák's multiple comparisons test** | **Mean1** | **Mean2** | **SEM1** | **SEM2** | **n1** | **n2** | **Adjusted P Value** |
| --- | --- | --- | --- | --- | --- | --- | --- |
| Ctl vs. PFR | 0.7600 | 0.9797 | 0.02651 | 0.02144 | 462 | 1140 | <0.001 |
| Ctl vs. PFR+Timp3 | 0.7600 | 0.7684 | 0.02651 | 0.02451 | 462 | 675 | >0.99 |
| PFR vs. PFR+Timp3 | 0.9797 | 0.7684 | 0.02144 | 0.02451 | 1140 | 675 | <0.001 |

**Figure 7G**

|  | **Ctl** | **PFR** | **PFR+TIMP3** |
| --- | --- | --- | --- |
| Number of values | 19 | 16 | 17 |
|  |  |  |  |
| Minimum | 8.00 | 15.00 | 11.00 |
| 25% Percentile | 12.00 | 16.25 | 13.50 |
| Median | 13.00 | 18.00 | 15.00 |
| 75% Percentile | 16.00 | 19.00 | 17.00 |
| Maximum | 17.00 | 22.00 | 19.00 |
| Range | 9.00 | 7.00 | 8.00 |
|  |  |  |  |
| Mean | 13.63 | 17.94 | 15.06 |
| Std. Deviation | 2.891 | 1.948 | 2.331 |
| Std. Error of Mean | 0.6632 | 0.4871 | 0.5654 |

| **Šídák's multiple comparisons test** | **Mean1** | **Mean2** | **SEM1** | **SEM2** | **n1** | **n2** | **Adjusted P Value** |
| --- | --- | --- | --- | --- | --- | --- | --- |
| Ctl vs. PFR | 13.63 | 17.94 | 0.663 | 0.565 | 19 | 16 | <0.001 |
| Ctl vs. PFR+Timp3 | 13.63 | 15.06 | 0.663 | 0.487 | 19 | 17 | 0.24 |
| PFR vs. PFR+Timp3 | 17.94 | 15.06 | 0.565 | 0.487 | 16 | 17 | 0.004 |
