# Supplementary material for: Activity-dependent extracellular proteolytic cascade cleaves the ECM component brevican to promote structural plasticity
Source: EMBO Rep. 2025 Nov 19;27(1):163–85. doi: 10.1038/s44319-025-00644-w (PMC12796228; doi:10.1038/s44319-025-00644-w)
Supplement: Supplementary file 8 — Table EV8 [file 44319_2025_644_MOESM8_ESM.docx]

**Table EV8**

Figure EV4

145 kDa

|  | **PFR** | **PFR+Anisomycin** |
| --- | --- | --- |
| Number of values | 4 | 4 |
|  |  |  |
| Minimum | 1.141 | 1.177 |
| 25% Percentile | 1.146 | 1.188 |
| Median | 1.215 | 1.279 |
| 75% Percentile | 1.491 | 1.735 |
| Maximum | 1.564 | 1.868 |
| Range | 0.4229 | 0.6905 |
|  |  |  |
| Mean | 1.284 | 1.400 |
| Std. Deviation | 0.1954 | 0.3187 |
| Std. Error of Mean | 0.09771 | 0.1593 |

| **Šídák's multiple comparisons test** | **Mean1** | **Mean2** | **SEM1** | **SEM2** | **n1** | **n2** | **Adjusted P Value** |
| --- | --- | --- | --- | --- | --- | --- | --- |
| Ctl vs. PFR | 1 | 1.284 | 0.0 | 0.09771 | 4 | 4 | 0.26 |
| Ctl vs. PFR+Anisomycin | 1 | 1.400 | 0.0 | 0.1593 | 4 | 4 | 0.08 |
| PFR vs. PFR+Anisomycin | 1.284 | 1.400 | 0.09771 | 0.1593 | 4 | 4 | 0.85 |

53 kDa

|  | **PFR** | **PFR+Anisomycin** |
| --- | --- | --- |
| Number of values | 4 | 4 |
|  |  |  |
| Minimum | 1.161 | 1.244 |
| 25% Percentile | 1.167 | 1.264 |
| Median | 1.267 | 1.336 |
| 75% Percentile | 1.379 | 1.432 |
| Maximum | 1.389 | 1.461 |
| Range | 0.2274 | 0.2172 |
|  |  |  |
| Mean | 1.271 | 1.344 |
| Std. Deviation | 0.1156 | 0.08959 |
| Std. Error of Mean | 0.05778 | 0.04479 |

| **Šídák's multiple comparisons test** | **Mean1** | **Mean2** | **SEM1** | **SEM2** | **n1** | **n2** | **Adjusted P Value** |
| --- | --- | --- | --- | --- | --- | --- | --- |
| Ctl vs. PFR | 1 | 1.271 | 0.0 | 0.05778 | 4 | 4 | 0.04 |
| Ctl vs. PFR+Anisomycin | 1 | 1.344 | 0.0 | 0.04479 | 4 | 4 | <0.001 |
| PFR vs. PFR+Anisomycin | 1.271 | 1.344 | 0.05778 | 0.04479 | 4 | 4 | 0.58 |
