# Supplementary figures and images for: Activity-dependent extracellular proteolytic cascade cleaves the ECM component brevican to promote structural plasticity (part 1 of 3)
Source: EMBO Rep. 2025 Nov 19;27(1):163–85. doi: 10.1038/s44319-025-00644-w (PMC12796228; doi:10.1038/s44319-025-00644-w)

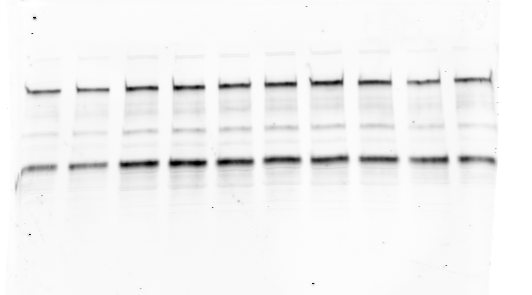

Supplement: Supplementary file 10 — Source data Fig. 1 [file 44319_2025_644_MOESM10_ESM.zip › Figure 1/Figure 1C MsBC.tif]

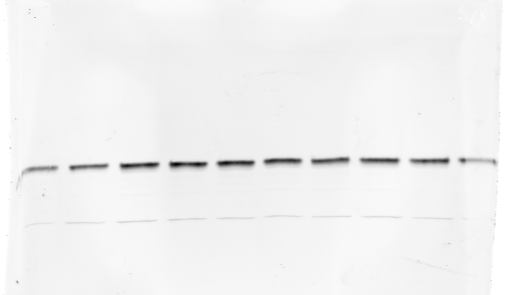

Supplement: Supplementary file 10 — Source data Fig. 1 [file 44319_2025_644_MOESM10_ESM.zip › Figure 1/Figure 1C Neo.tif]

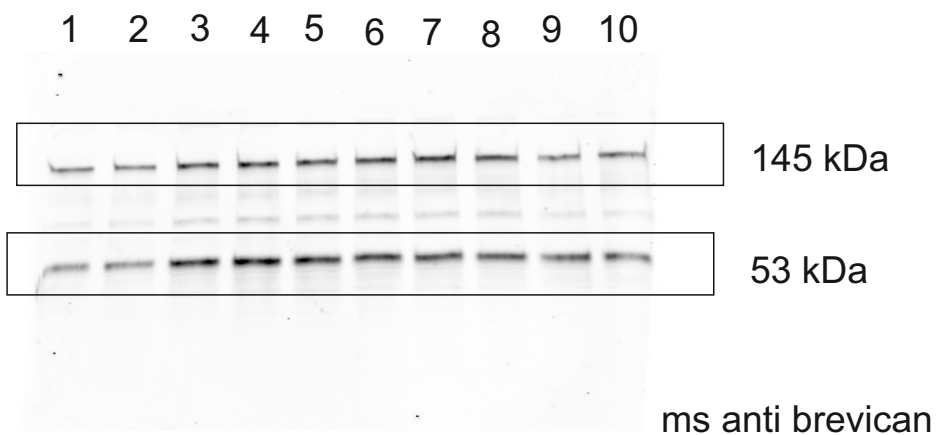

1&2 Control 0 min  
3&4 15 min PFR  
5&6 45 min PFR  
7&8 90 min PFR  
9&10 180 min PFR

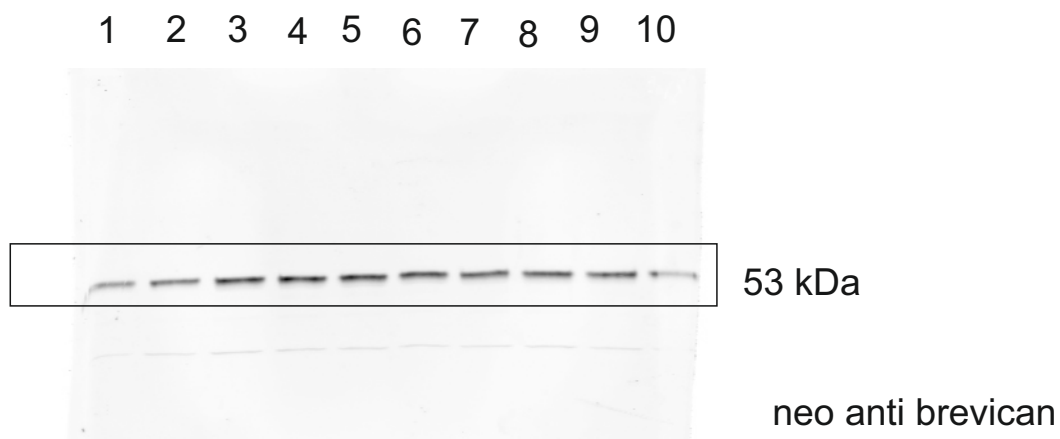

Supplement: Supplementary file 10 — Source data Fig. 1 [file 44319_2025_644_MOESM10_ESM.zip › Figure 1/Figure 1c.pdf]

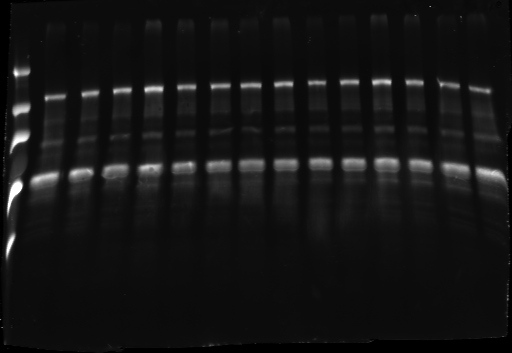

Supplement: Supplementary file 10 — Source data Fig. 1 [file 44319_2025_644_MOESM10_ESM.zip › Figure 1/Figure 1E Ms BC.tif]

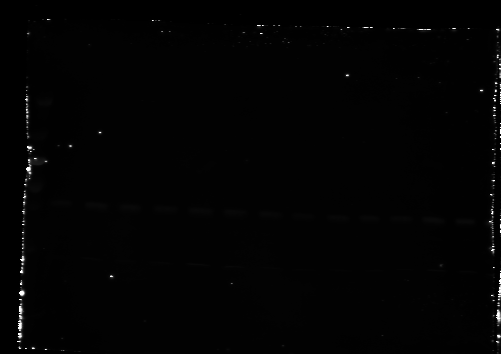

Supplement: Supplementary file 10 — Source data Fig. 1 [file 44319_2025_644_MOESM10_ESM.zip › Figure 1/Figure 1E Neo.tif]

1 PFR  
2 PFR & GM6001  
3 PFR & Pic.  
4 PFR & TIMP3  
5 Ctl

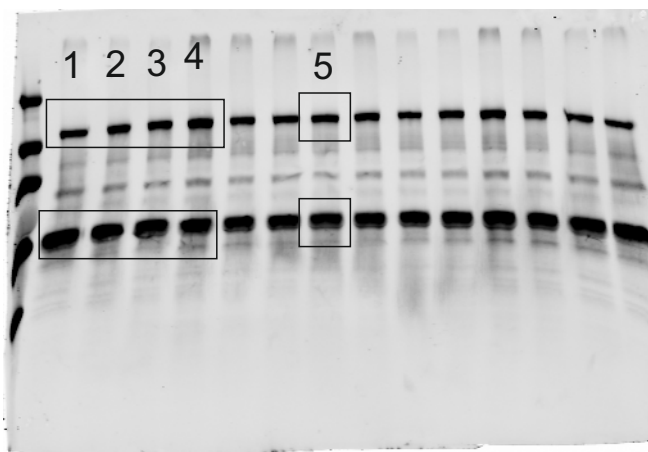

ms anti brevican

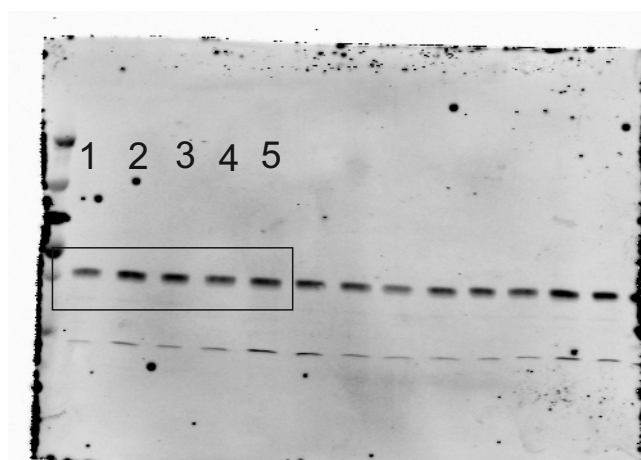

neo anti brevican

1 Ctl  
2 PFR  
3 PFR & TIMP3  
4 PFR & Pic.  
5 PFR & Gm6001

Supplement: Supplementary file 10 — Source data Fig. 1 [file 44319_2025_644_MOESM10_ESM.zip › Figure 1/Figure 1E.pdf]

neo anti aggrecan

1&2 Control 0 min

3&4 PFR

5&6 PFR & Timp3

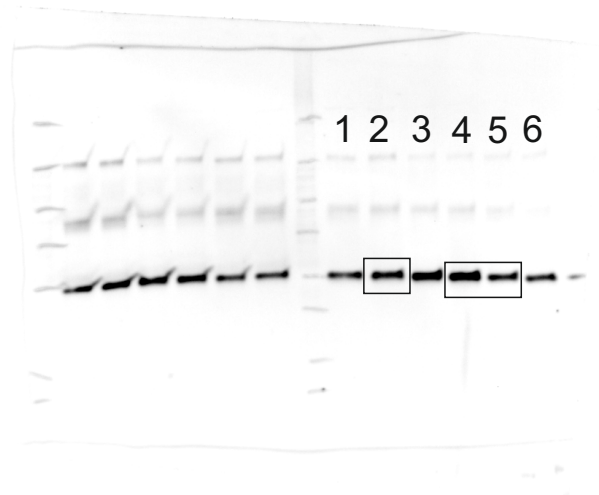

Supplement: Supplementary file 10 — Source data Fig. 1 [file 44319_2025_644_MOESM10_ESM.zip › Figure 1/Figure 1I.pdf]

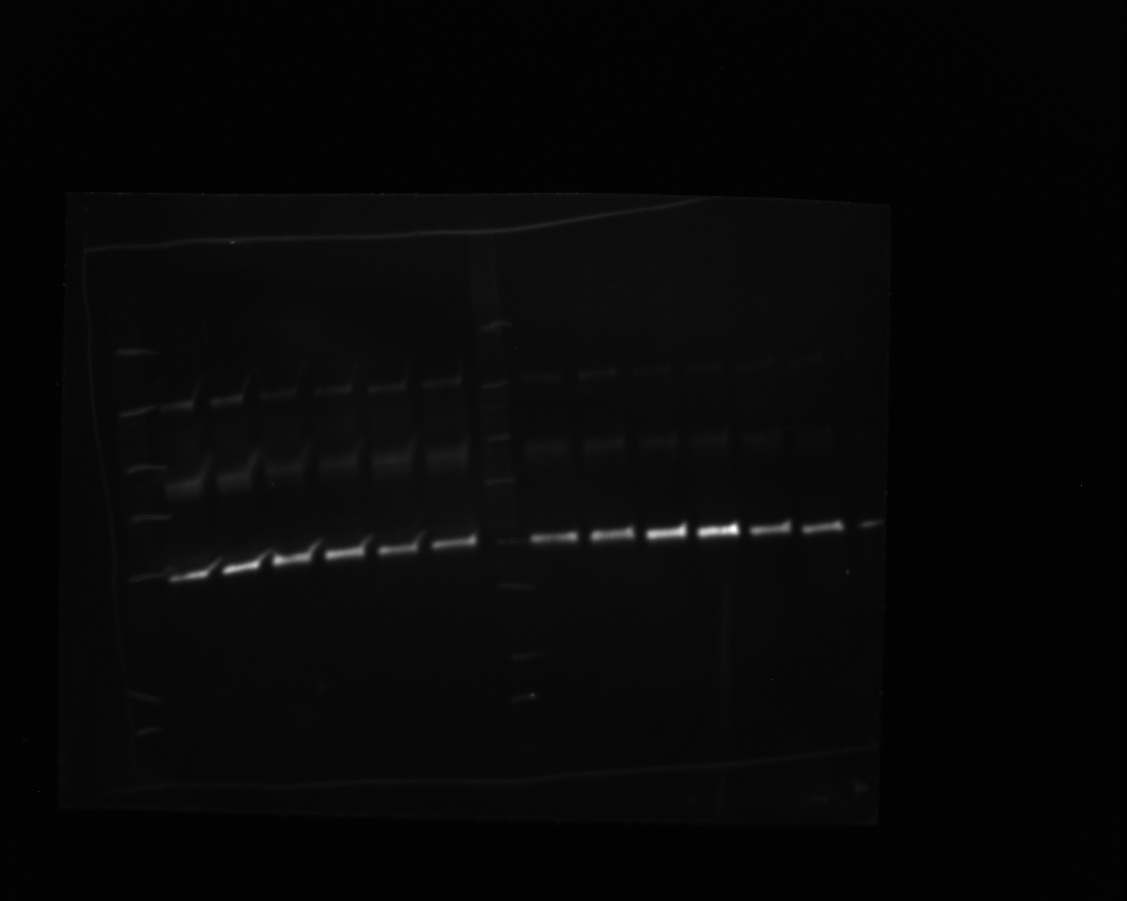

Supplement: Supplementary file 10 — Source data Fig. 1 [file 44319_2025_644_MOESM10_ESM.zip › Figure 1/Figure 1I.tif]

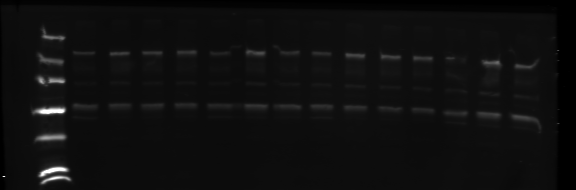

Supplement: Supplementary file 11 — Source data Fig. 2 [file 44319_2025_644_MOESM11_ESM.zip › Figure 2/Figure 2A MsBC-Ctl-PFR-PFR+Furini II.tif]

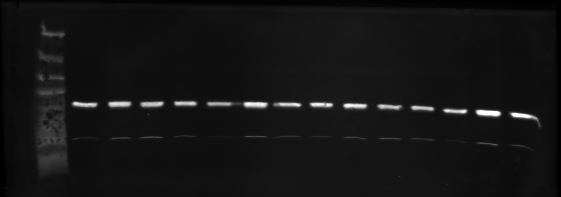

Supplement: Supplementary file 11 — Source data Fig. 2 [file 44319_2025_644_MOESM11_ESM.zip › Figure 2/Figure 2A Neo-Ctl-PFR-PFR+Furini I.tif]

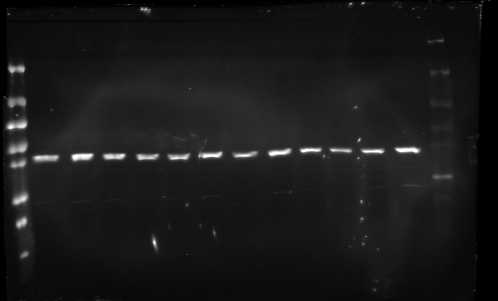

Supplement: Supplementary file 11 — Source data Fig. 2 [file 44319_2025_644_MOESM11_ESM.zip › Figure 2/Figure 2A Neo-Ctl-PFR-PFR+Furini II.tif]

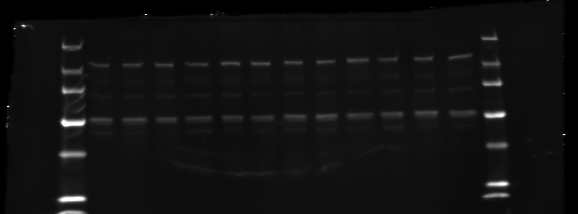

Supplement: Supplementary file 11 — Source data Fig. 2 [file 44319_2025_644_MOESM11_ESM.zip › Figure 2/Figure 2A PCi MS BC.tif]

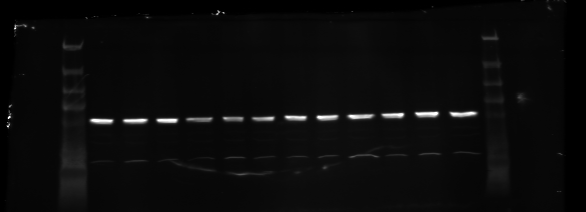

Supplement: Supplementary file 11 — Source data Fig. 2 [file 44319_2025_644_MOESM11_ESM.zip › Figure 2/Figure 2A PCi Neo.tif]

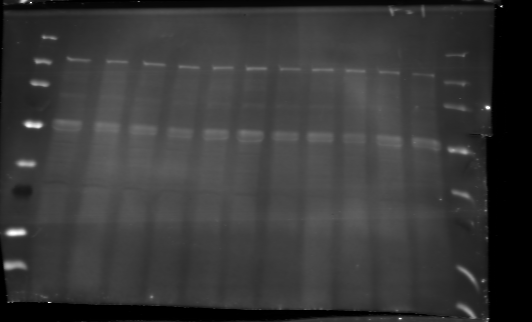

Supplement: Supplementary file 11 — Source data Fig. 2 [file 44319_2025_644_MOESM11_ESM.zip › Figure 2/Figure 2A_msBC-Ctl-PFR-PFRFurini.tif]

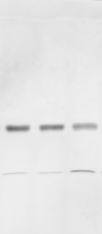

Supplement: Supplementary file 11 — Source data Fig. 2 [file 44319_2025_644_MOESM11_ESM.zip › Figure 2/Figure 2C_Neo-APMA-PFR APMA-Ctl.tif]

neo anti brevican

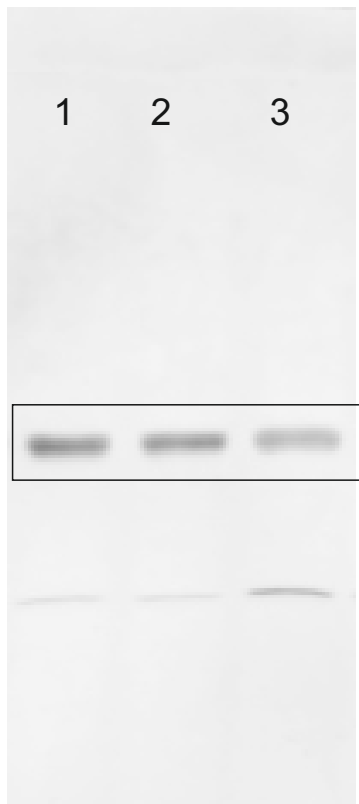

1 APMA  
2 APMA & PFR  
3 Ctl

Supplement: Supplementary file 11 — Source data Fig. 2 [file 44319_2025_644_MOESM11_ESM.zip › Figure 2/Figure 2C_image.pdf]

1 Ctl  
2 PFR  
3 PFR & Furini II

ms anti BC

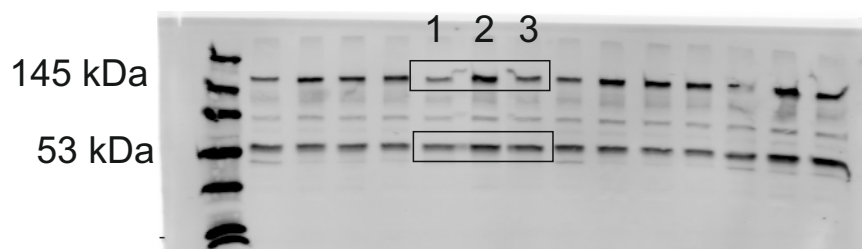

1 Ctl  
2 PFR  
3 PFR & PCI

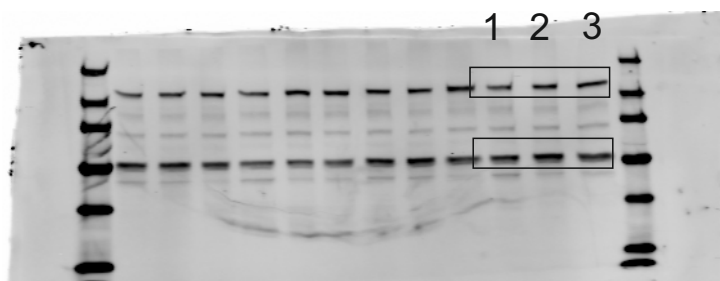

1 Ctl  
2 PFR  
3 PFR & Furini I

ms anti BC

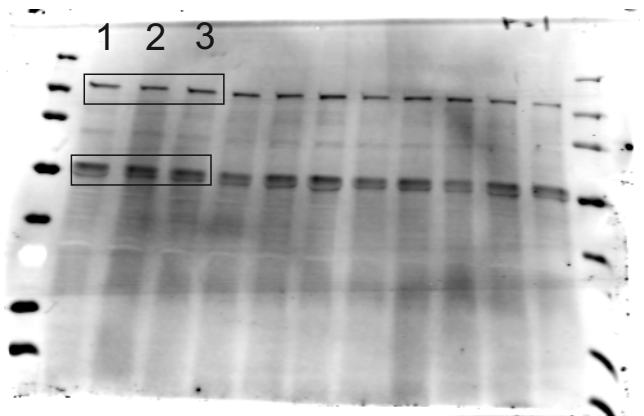

ms anti BC

Supplement: Supplementary file 11 — Source data Fig. 2 [file 44319_2025_644_MOESM11_ESM.zip › Figure 2/figure 2A ms BC.pdf]

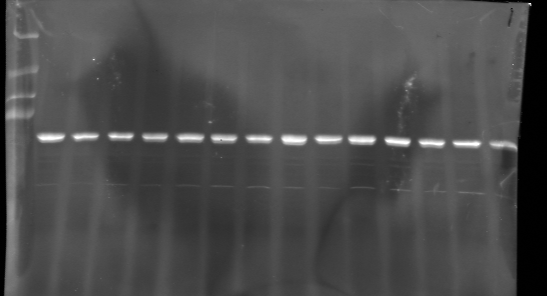

Supplement: Supplementary file 12 — Source data Fig. 3 [file 44319_2025_644_MOESM12_ESM.zip › Figure 3/Figure 3A -Neo_ PFR-PFR+CBX-PFR+Endo.tif]

Neo anti brevican

- 1 PFR
- 2 PFR & CBX
- 3 PFR & Endo
- 4 Ctl

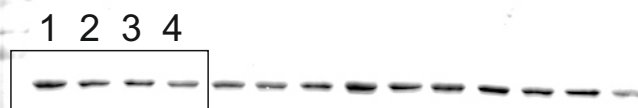

Supplement: Supplementary file 12 — Source data Fig. 3 [file 44319_2025_644_MOESM12_ESM.zip › Figure 3/Figure 3A.pdf]

Neo anti brevican

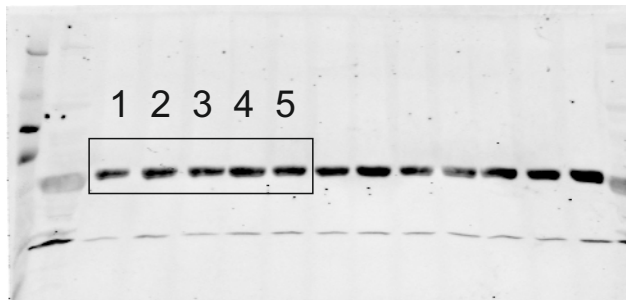

- 1 Ctl
- 2 D-serine
- 3 PFR + D-serine
- 4 PFR + D-serine + CBX
- 5 D-serine + PFR + D-APV

Supplement: Supplementary file 12 — Source data Fig. 3 [file 44319_2025_644_MOESM12_ESM.zip › Figure 3/Figure 3C.pdf]

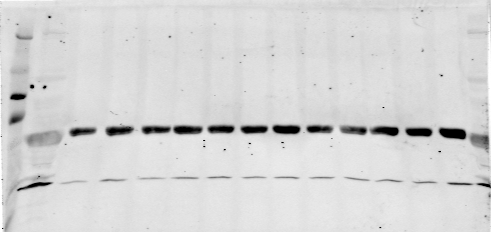

Supplement: Supplementary file 12 — Source data Fig. 3 [file 44319_2025_644_MOESM12_ESM.zip › Figure 3/Figure 3C.tif]

# Ms anti brevican

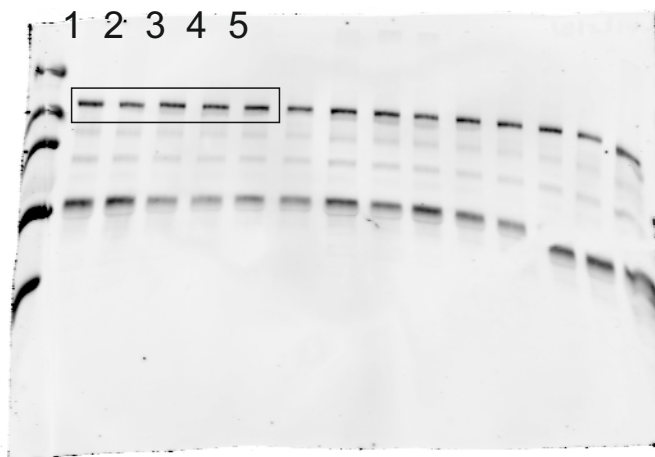

- 1 PFR
- 2 CNQX
- 3 Mk801
- 4 Nifedipine
- 5 Ctl

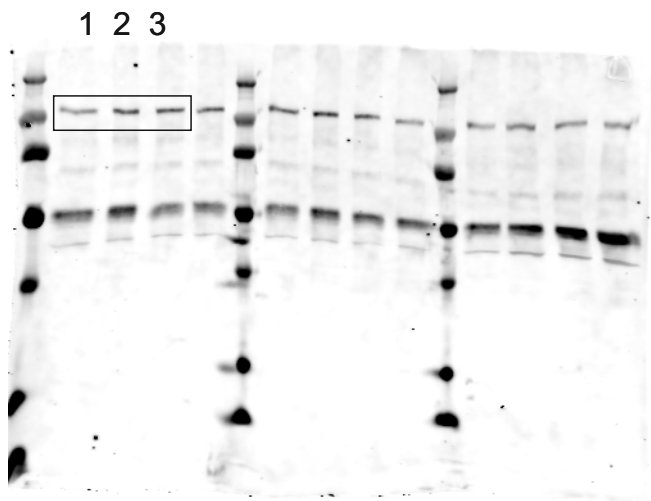

- 1 Ctl
- 2 PFR
- 3 PFR & Ro256981

Supplement: Supplementary file 13 — Source data Fig. 4 [file 44319_2025_644_MOESM13_ESM.zip › Figure 4/figure 4 A ms BC.pdf]

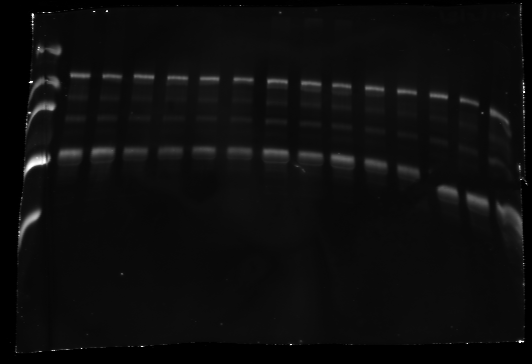

Supplement: Supplementary file 13 — Source data Fig. 4 [file 44319_2025_644_MOESM13_ESM.zip › Figure 4/Figure 4A Ms BC_PFR-PFR+CNQX-PFR+MK801-PFR+Nifedipine.tif]

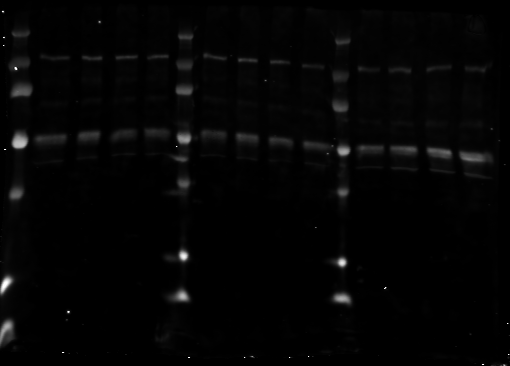

Supplement: Supplementary file 13 — Source data Fig. 4 [file 44319_2025_644_MOESM13_ESM.zip › Figure 4/Figure 4A msBC ctl-PFR-RO.tif]

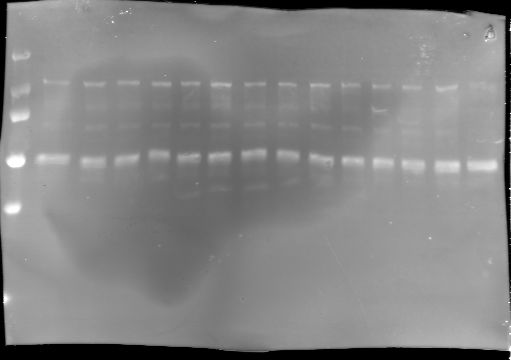

Supplement: Supplementary file 13 — Source data Fig. 4 [file 44319_2025_644_MOESM13_ESM.zip › Figure 4/Figure 4A MsBC_Ctl-PFR-PFR+RO.tif]

# Neo anti brevican

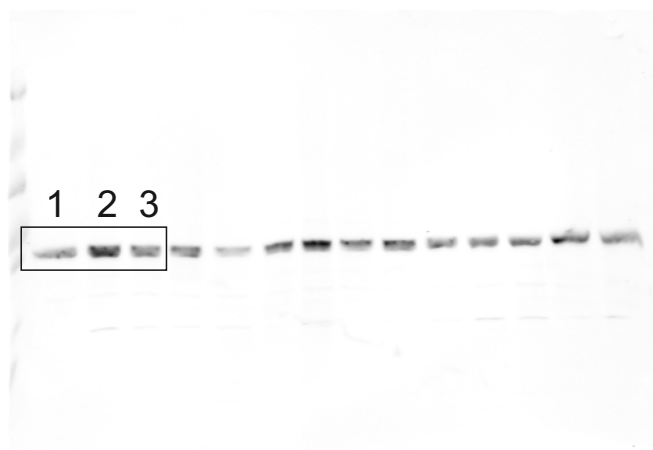

- 1 Ctl
- 2 PFR
- 3 RO 256981

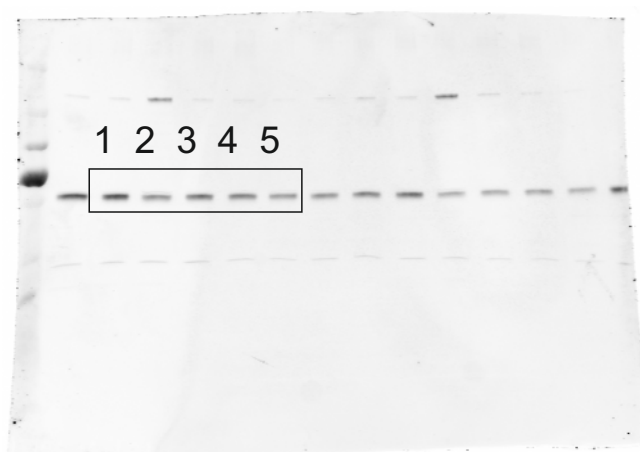

- 1 PFR
- 2 CNQX
- 3 Mk801
- 4 Nifedipine
- 5 Ctl

Supplement: Supplementary file 13 — Source data Fig. 4 [file 44319_2025_644_MOESM13_ESM.zip › Figure 4/Figure 4A neo.pdf]

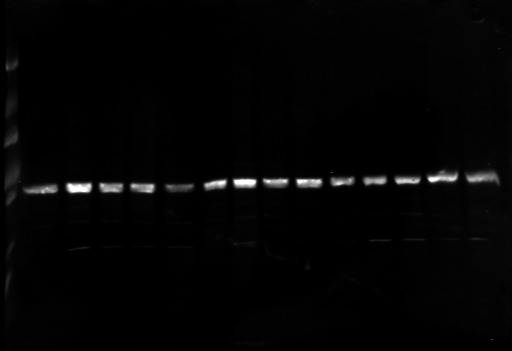

Supplement: Supplementary file 13 — Source data Fig. 4 [file 44319_2025_644_MOESM13_ESM.zip › Figure 4/Figure 4A Neo_Ctl-PFR-PFR+RO.tif]

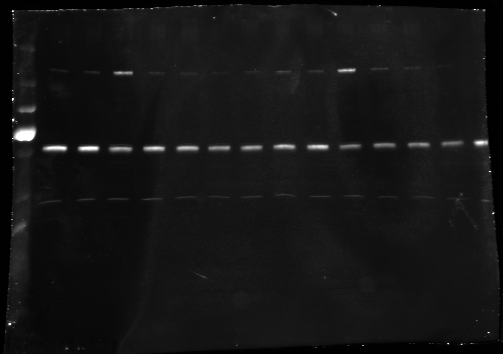

Supplement: Supplementary file 13 — Source data Fig. 4 [file 44319_2025_644_MOESM13_ESM.zip › Figure 4/Figure 4A Neo_PFR-PFR+CNQX-PFR+MK801-PFR+Nifedipine.tif]

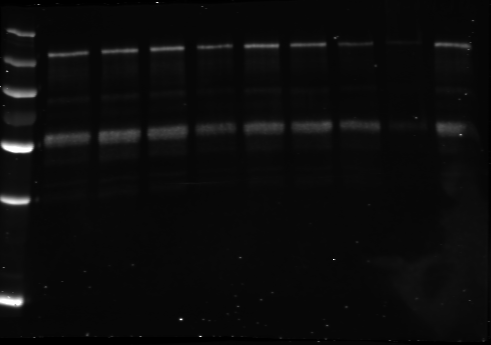

Supplement: Supplementary file 13 — Source data Fig. 4 [file 44319_2025_644_MOESM13_ESM.zip › Figure 4/Figure 4D MsBC_Ctl-PFR-AIP-PFR+AIP.tif]

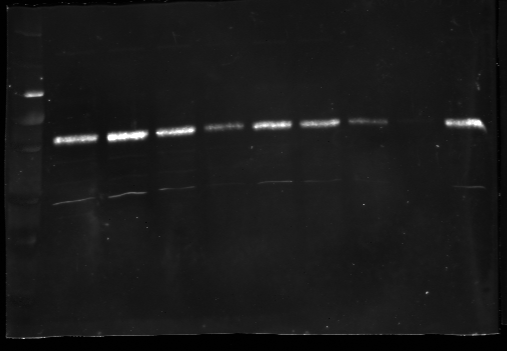

Supplement: Supplementary file 13 — Source data Fig. 4 [file 44319_2025_644_MOESM13_ESM.zip › Figure 4/Figure 4D Neo_Ctl-PFR-AIP-PFR+AIP.tif]

ms anti brevican

1 2 3 4

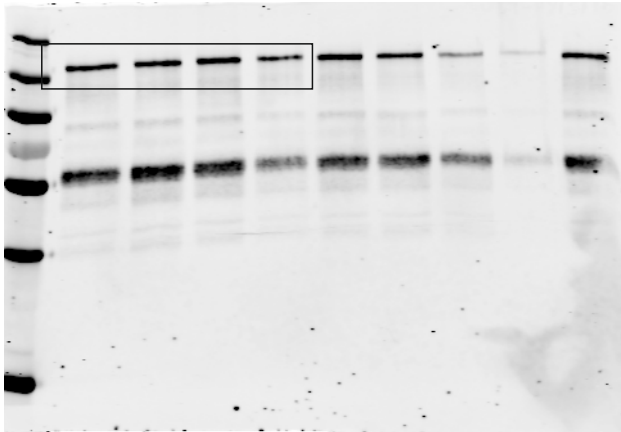

1 Ctl  
2 PFR  
3 AIP  
4 AIP & PFR

Neo anti brevican

1 2 3 4

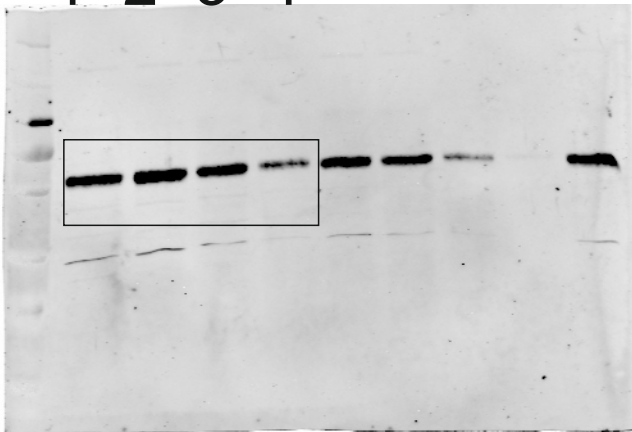

Supplement: Supplementary file 13 — Source data Fig. 4 [file 44319_2025_644_MOESM13_ESM.zip › Figure 4/Figure 4D.pdf]

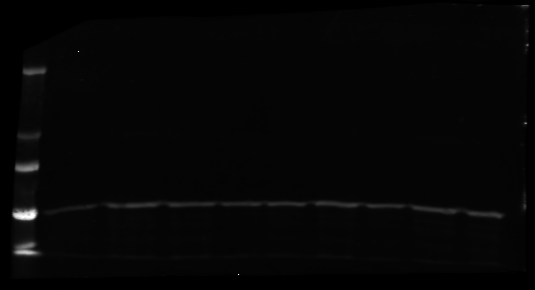

Supplement: Supplementary file 14 — Source data Fig. 5 [file 44319_2025_644_MOESM14_ESM.zip › Figure 5/Figure 5A CamKII .tif]

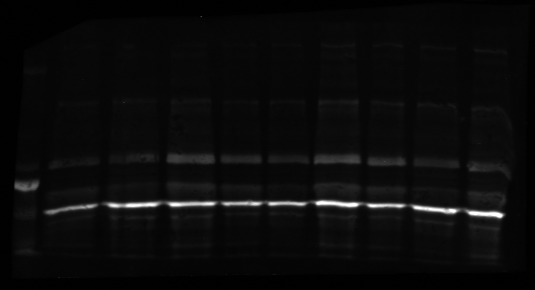

Supplement: Supplementary file 14 — Source data Fig. 5 [file 44319_2025_644_MOESM14_ESM.zip › Figure 5/Figure 5A pCaMKII.tif]

1 Ctl  
2 PFR  
3 PFR & TIMP3

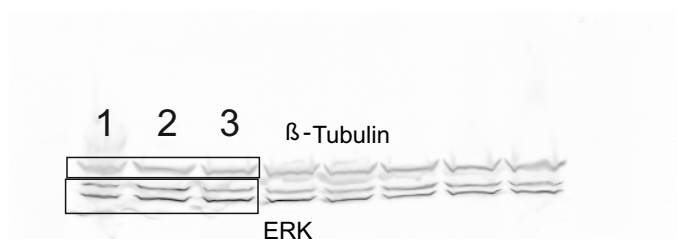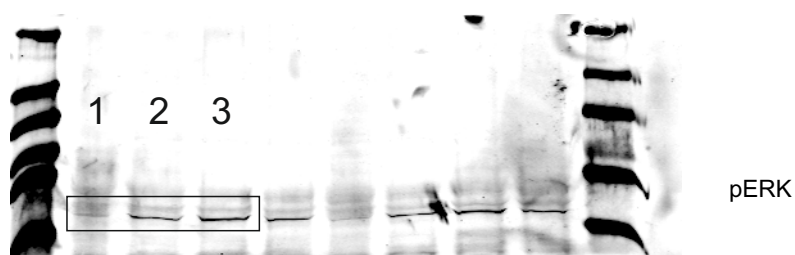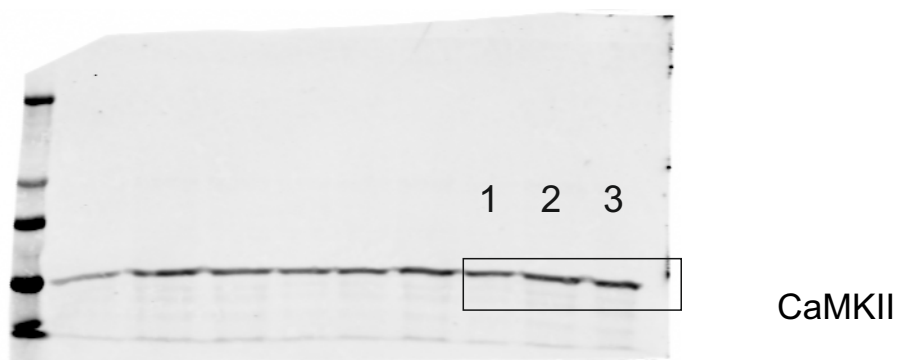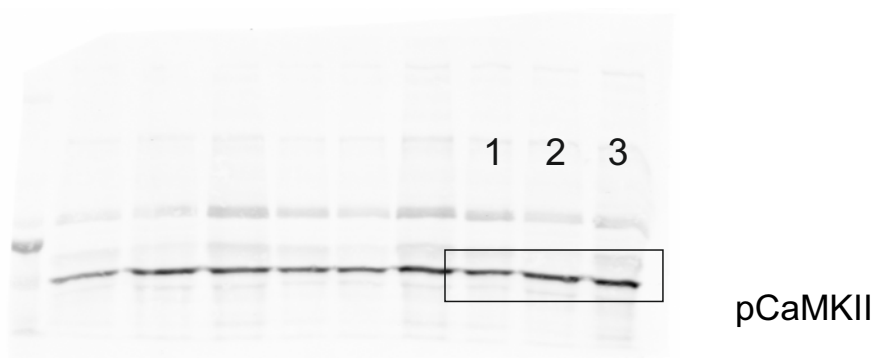

Figure 5

Supplement: Supplementary file 14 — Source data Fig. 5 [file 44319_2025_644_MOESM14_ESM.zip › Figure 5/Figure 5A.pdf]

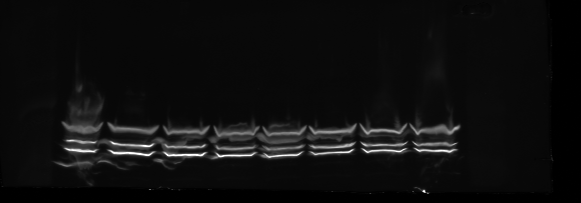

Supplement: Supplementary file 14 — Source data Fig. 5 [file 44319_2025_644_MOESM14_ESM.zip › Figure 5/Figure 5A_ERK&B-Tubulin.tif]

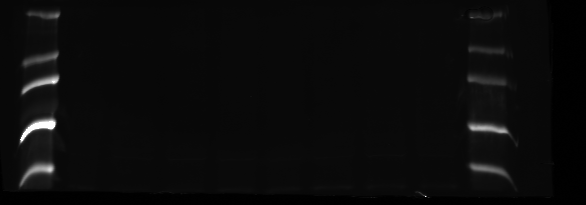

Supplement: Supplementary file 14 — Source data Fig. 5 [file 44319_2025_644_MOESM14_ESM.zip › Figure 5/Figure 5A_pERK.tif]

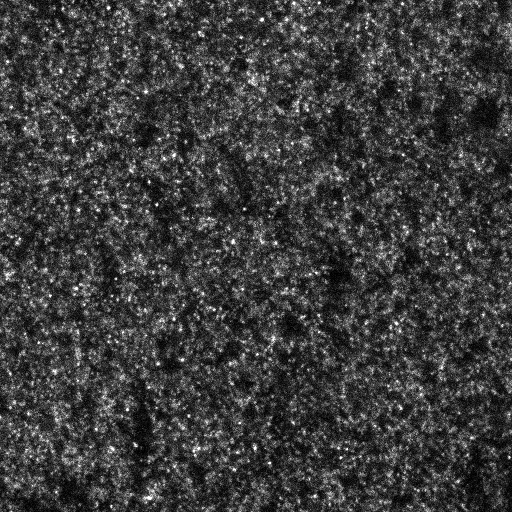

Supplement: Supplementary file 15 — Source data Fig. 6 [file 44319_2025_644_MOESM15_ESM.zip › Figure 6/6G/Ctl/Ctl3_2-1-1-3/AVG_C2-ctl1_exp14aug_rb399-ctl3_2-1-1-3.tif512-1.tif]

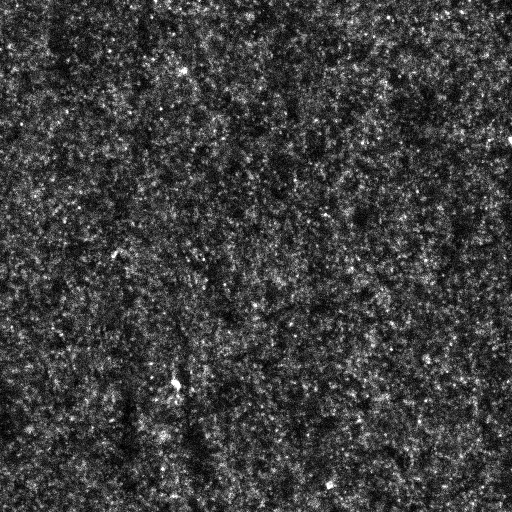

Supplement: Supplementary file 15 — Source data Fig. 6 [file 44319_2025_644_MOESM15_ESM.zip › Figure 6/6G/Ctl/Ctl3_2-1-1-3/AVG_C2-ctl1_exp14aug_rb399-ctl3_2-1-1-3.tif512-2.tif]

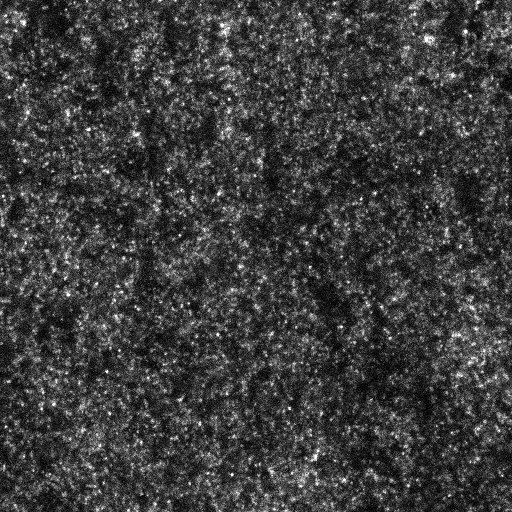

Supplement: Supplementary file 15 — Source data Fig. 6 [file 44319_2025_644_MOESM15_ESM.zip › Figure 6/6G/Ctl/Ctl3_2-1-1-3/AVG_C2-ctl1_exp14aug_rb399-ctl3_2-1-1-3.tif512-3.tif]

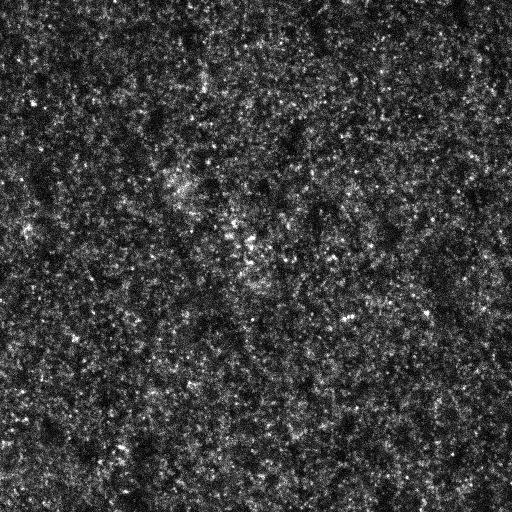

Supplement: Supplementary file 15 — Source data Fig. 6 [file 44319_2025_644_MOESM15_ESM.zip › Figure 6/6G/Ctl/Ctl3_2-1-1-3/AVG_C2-ctl1_exp14aug_rb399-ctl3_2-1-1-3.tif512-4.tif]

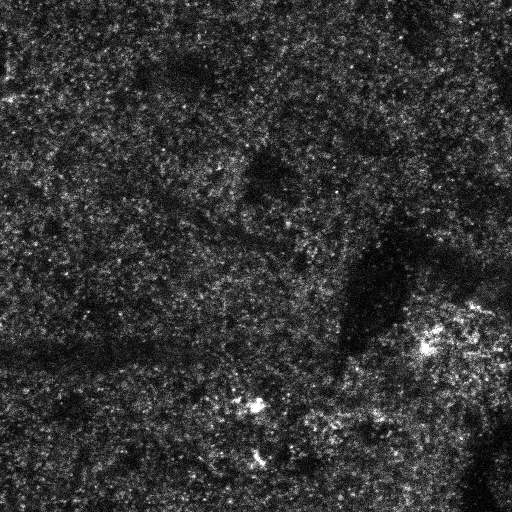

Supplement: Supplementary file 15 — Source data Fig. 6 [file 44319_2025_644_MOESM15_ESM.zip › Figure 6/6G/Ctl/Slice1_3-6-1/AVG_C3-ctl_exp14aug_rb399- slice1_3-6-1.tif512-1.tif]

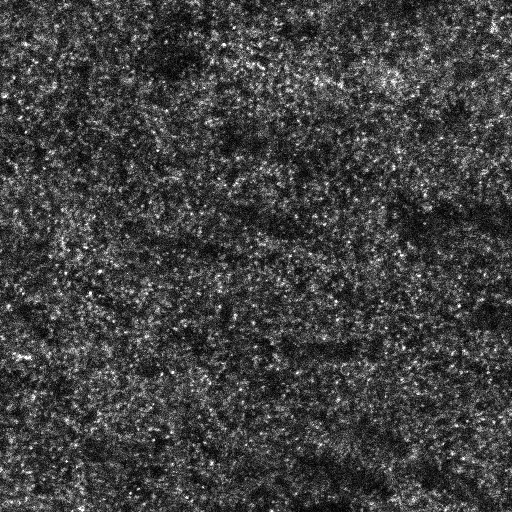

Supplement: Supplementary file 15 — Source data Fig. 6 [file 44319_2025_644_MOESM15_ESM.zip › Figure 6/6G/Ctl/Slice1_3-6-1/AVG_C3-ctl_exp14aug_rb399- slice1_3-6-1.tif512-2.tif]

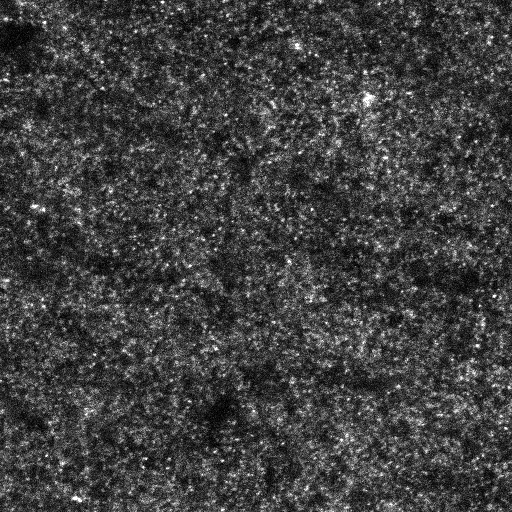

Supplement: Supplementary file 15 — Source data Fig. 6 [file 44319_2025_644_MOESM15_ESM.zip › Figure 6/6G/Ctl/Slice1_3-6-1/AVG_C3-ctl_exp14aug_rb399- slice1_3-6-1.tif512-3.tif]

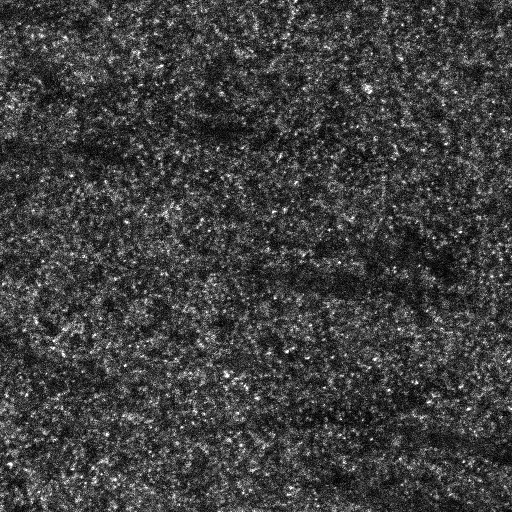

Supplement: Supplementary file 15 — Source data Fig. 6 [file 44319_2025_644_MOESM15_ESM.zip › Figure 6/6G/Ctl/Slice1_3-6-1/AVG_C3-ctl_exp14aug_rb399- slice1_3-6-1.tif512-4.tif]

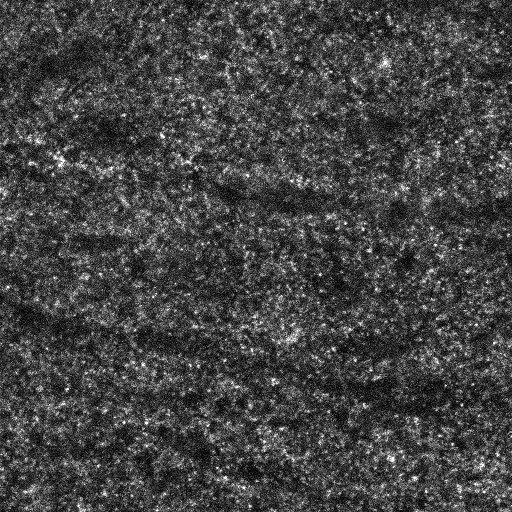

Supplement: Supplementary file 15 — Source data Fig. 6 [file 44319_2025_644_MOESM15_ESM.zip › Figure 6/6G/Ctl/Slice1_3-6-8/AVG_C3-ctl_exp14aug_rb399- slice1_3-6-8.tif512-1.tif]

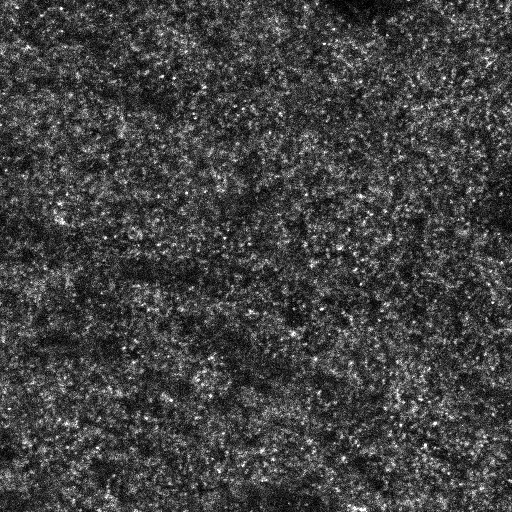

Supplement: Supplementary file 15 — Source data Fig. 6 [file 44319_2025_644_MOESM15_ESM.zip › Figure 6/6G/Ctl/Slice1_3-6-8/AVG_C3-ctl_exp14aug_rb399- slice1_3-6-8.tif512-2.tif]

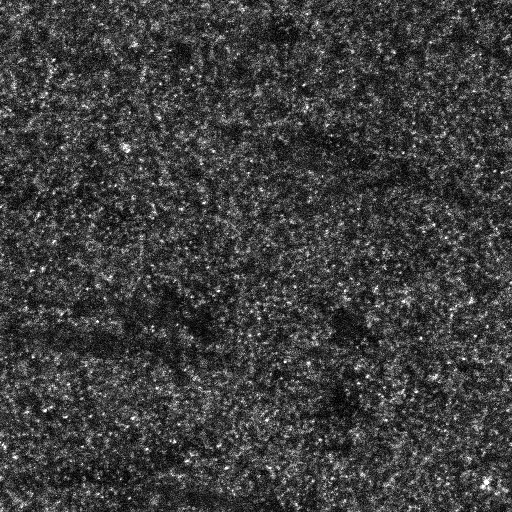

Supplement: Supplementary file 15 — Source data Fig. 6 [file 44319_2025_644_MOESM15_ESM.zip › Figure 6/6G/Ctl/Slice1_3-6-8/AVG_C3-ctl_exp14aug_rb399- slice1_3-6-8.tif512-3.tif]

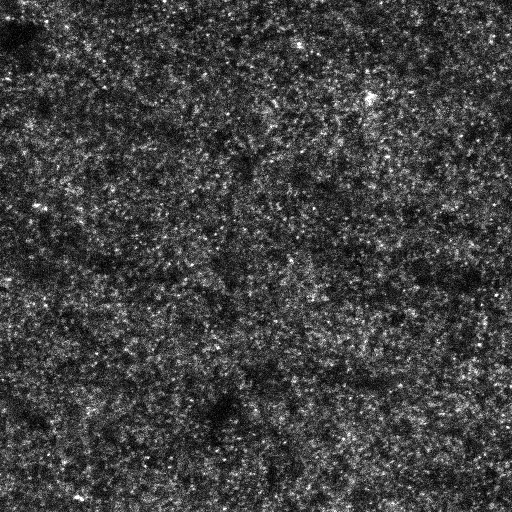

Supplement: Supplementary file 15 — Source data Fig. 6 [file 44319_2025_644_MOESM15_ESM.zip › Figure 6/6G/Ctl/Slice1_3-6-8/AVG_C3-ctl_exp14aug_rb399- slice1_3-6-8.tif512-4.tif]

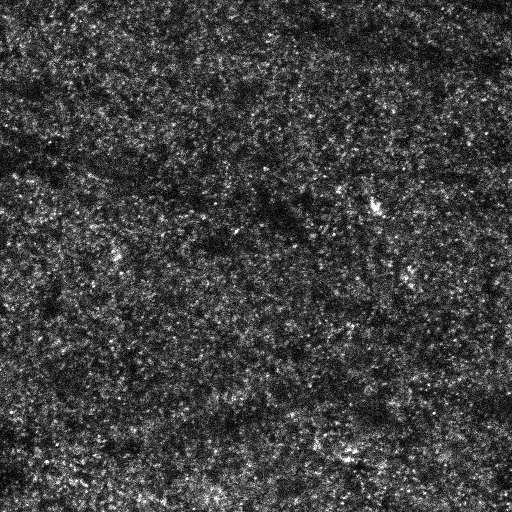

Supplement: Supplementary file 15 — Source data Fig. 6 [file 44319_2025_644_MOESM15_ESM.zip › Figure 6/6G/Ctl/Slice1_3-6-8/AVG_C3-ctl_exp14aug_rb399- slice1_3-6-8.tif512-5.tif]

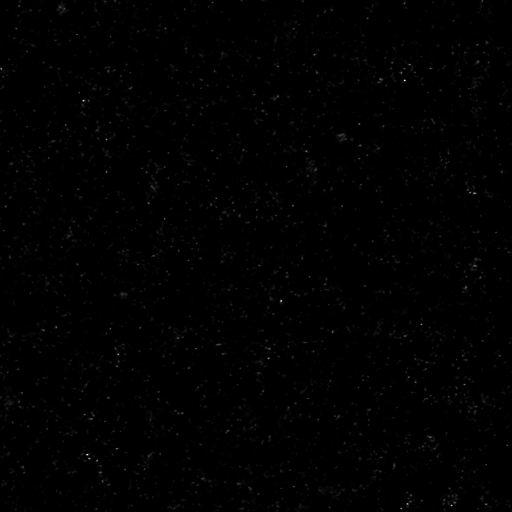

Supplement: Supplementary file 15 — Source data Fig. 6 [file 44319_2025_644_MOESM15_ESM.zip › Figure 6/6G/Ctl/Slice2_1-1-3/AVG_C2-ctl1_exp14aug_homer-slice2_1-1-3.tif512-1_deconv.tif]

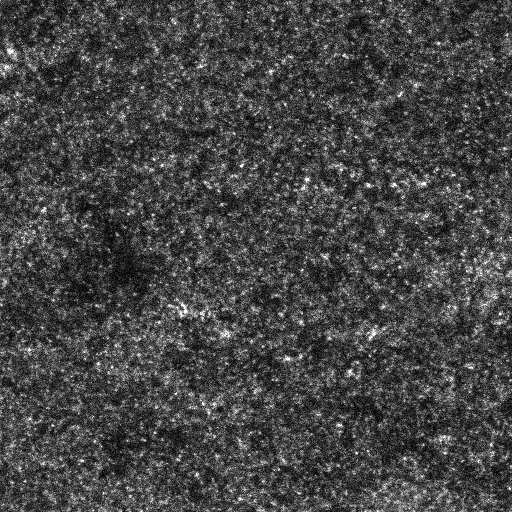

Supplement: Supplementary file 15 — Source data Fig. 6 [file 44319_2025_644_MOESM15_ESM.zip › Figure 6/6G/Ctl/Slice2_1-1-3/AVG_C3-ctl1_exp14aug_rb399 - slice2_1-1-3.tif512-2.tif]

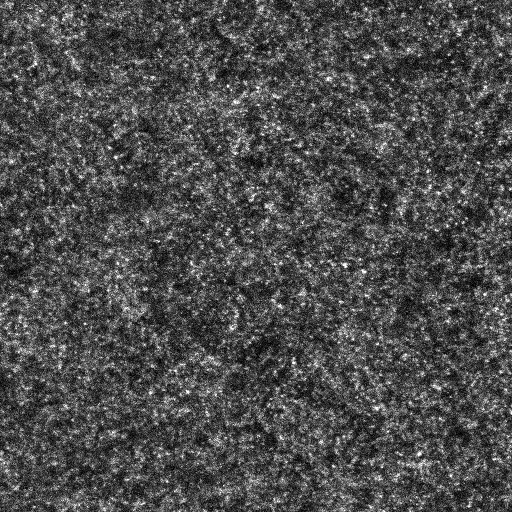

Supplement: Supplementary file 15 — Source data Fig. 6 [file 44319_2025_644_MOESM15_ESM.zip › Figure 6/6G/Ctl/Slice2_1-1-3/AVG_C3-ctl1_exp14aug_rb399 - slice2_1-1-3.tif512-3.tif]

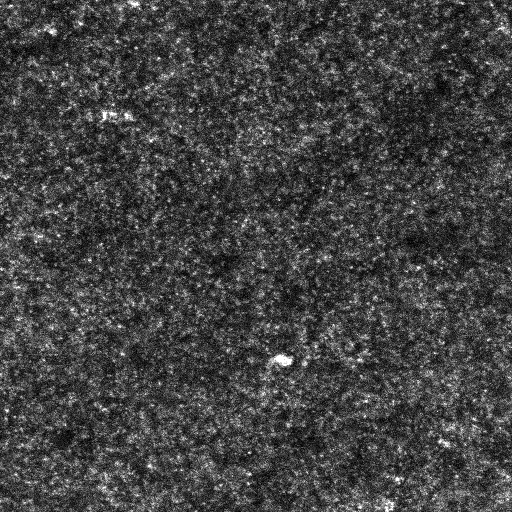

Supplement: Supplementary file 15 — Source data Fig. 6 [file 44319_2025_644_MOESM15_ESM.zip › Figure 6/6G/Ctl/Slice2_1-1-3/AVG_C3-ctl1_exp14aug_rb399 - slice2_1-1-3.tif512-4.tif]

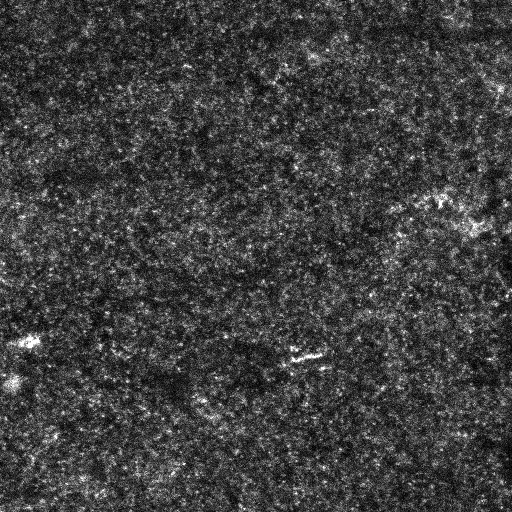

Supplement: Supplementary file 15 — Source data Fig. 6 [file 44319_2025_644_MOESM15_ESM.zip › Figure 6/6G/Ctl/Slice2_1-1-3/AVG_C3-ctl1_exp14aug_rb399- slice2_1-1-3.tif512-1.tif]

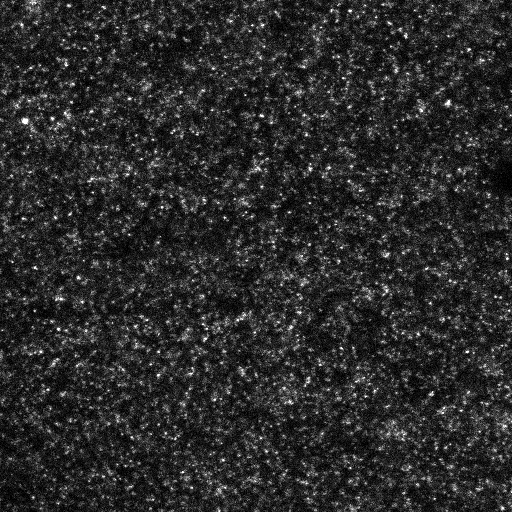

Supplement: Supplementary file 15 — Source data Fig. 6 [file 44319_2025_644_MOESM15_ESM.zip › Figure 6/6G/Ctl/Slice2_1-6-9/AVG_C3-ctl1_exp14aug_homer- slice2_1-6-9.tif512-1.tif]

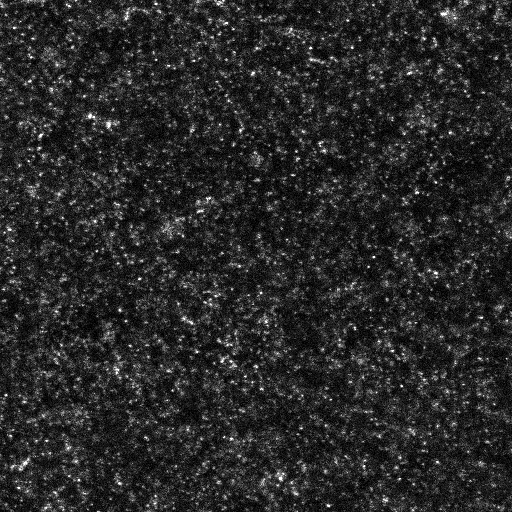

Supplement: Supplementary file 15 — Source data Fig. 6 [file 44319_2025_644_MOESM15_ESM.zip › Figure 6/6G/Ctl/Slice2_1-6-9/AVG_C3-ctl1_exp14aug_homer- slice2_1-6-9.tif512-2.tif]

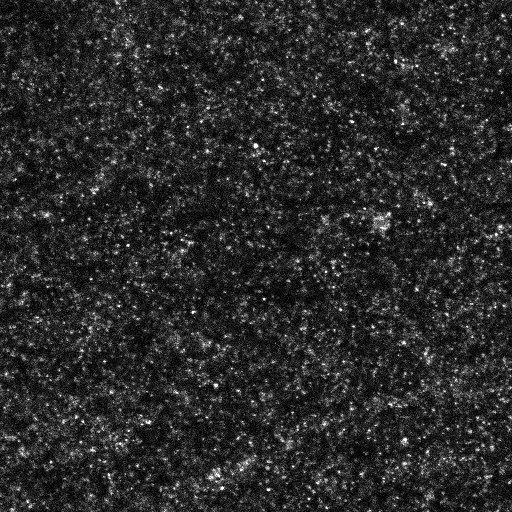

Supplement: Supplementary file 15 — Source data Fig. 6 [file 44319_2025_644_MOESM15_ESM.zip › Figure 6/6G/Ctl/Slice2_1-6-9/AVG_C3-ctl1_exp14aug_homer- slice2_1-6-9.tif512-3.tif]

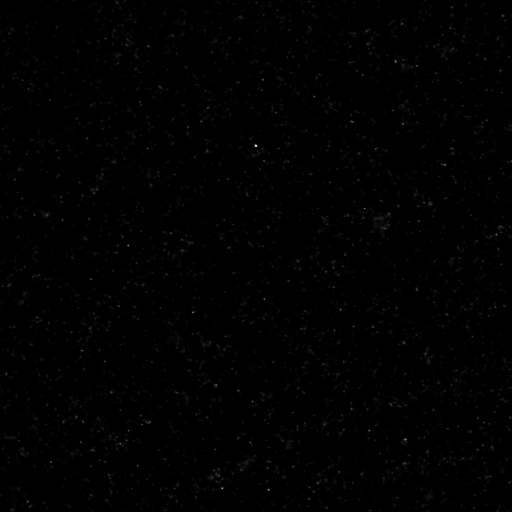

Supplement: Supplementary file 15 — Source data Fig. 6 [file 44319_2025_644_MOESM15_ESM.zip › Figure 6/6G/Ctl/Slice2_1-6-9/AVG_C3-ctl1_exp14aug_homer- slice2_1-6-9.tif512-3_deconv.tif]

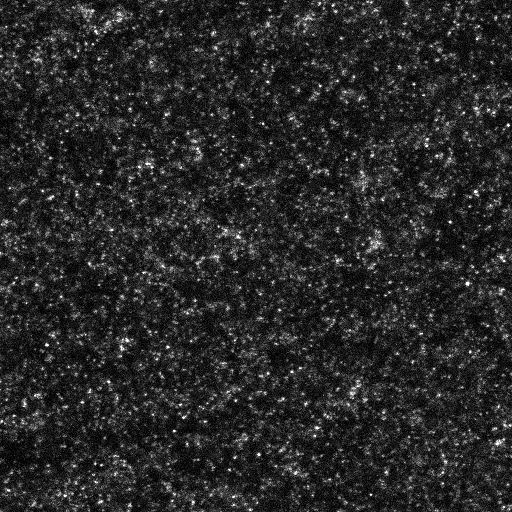

Supplement: Supplementary file 15 — Source data Fig. 6 [file 44319_2025_644_MOESM15_ESM.zip › Figure 6/6G/Ctl/Slice2_1-6-9/AVG_C3-ctl1_exp14aug_homer- slice2_1-6-9.tif512-4.tif]

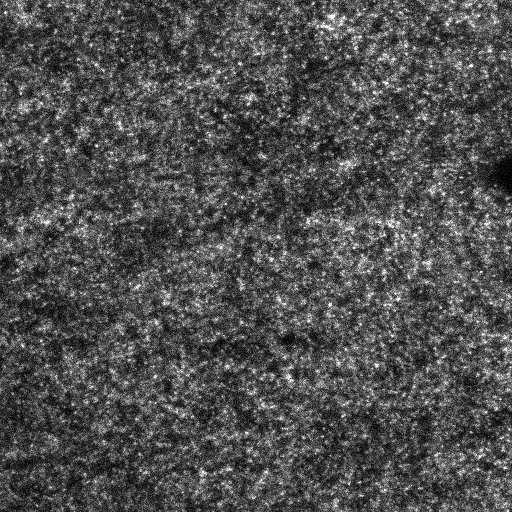

Supplement: Supplementary file 15 — Source data Fig. 6 [file 44319_2025_644_MOESM15_ESM.zip › Figure 6/6G/Ctl/Slice2_1-6-9/AVG_C3-ctl1_exp14aug_rb399- slice2_1-6-9.tif512-1.tif]

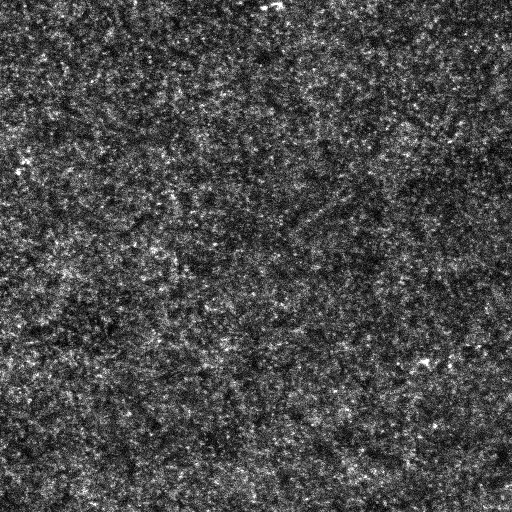

Supplement: Supplementary file 15 — Source data Fig. 6 [file 44319_2025_644_MOESM15_ESM.zip › Figure 6/6G/Ctl/Slice2_1-6-9/AVG_C3-ctl1_exp14aug_rb399- slice2_1-6-9.tif512-2.tif]

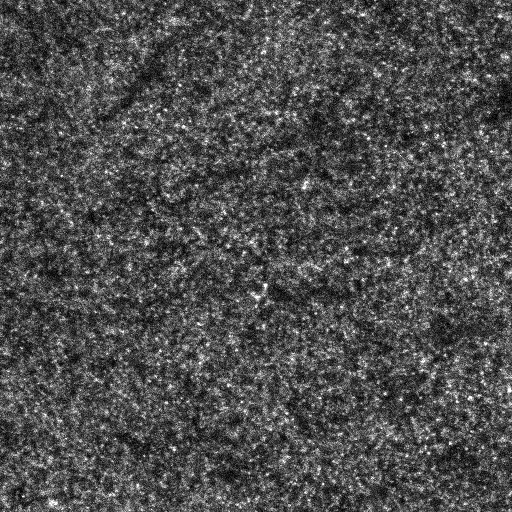

Supplement: Supplementary file 15 — Source data Fig. 6 [file 44319_2025_644_MOESM15_ESM.zip › Figure 6/6G/Ctl/Slice2_1-6-9/AVG_C3-ctl1_exp14aug_rb399- slice2_1-6-9.tif512-3.tif]

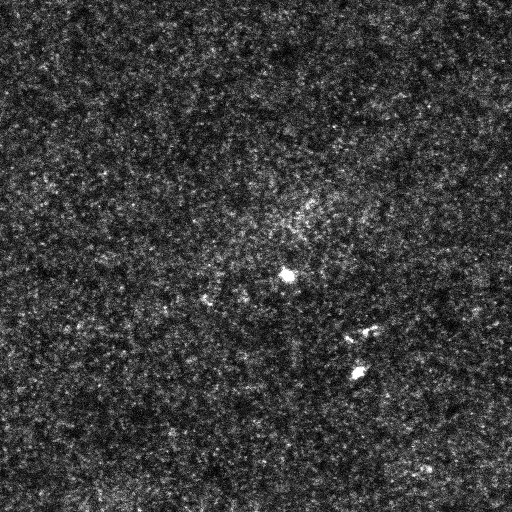

Supplement: Supplementary file 15 — Source data Fig. 6 [file 44319_2025_644_MOESM15_ESM.zip › Figure 6/6G/Ctl/Slice2_1-6-9/AVG_C3-ctl1_exp14aug_rb399- slice2_1-6-9.tif512-4.tif]

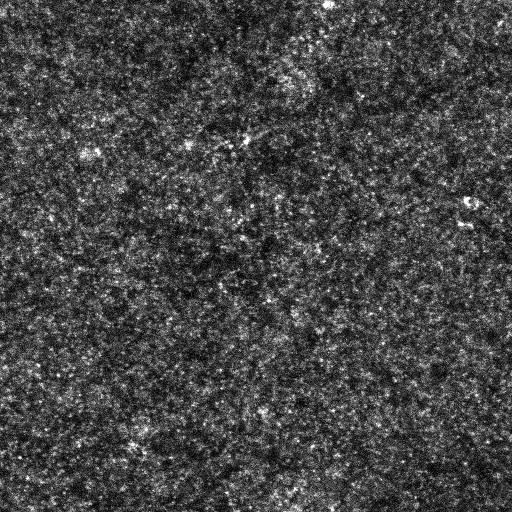

Supplement: Supplementary file 15 — Source data Fig. 6 [file 44319_2025_644_MOESM15_ESM.zip › Figure 6/6G/Ctl/Slice2_2-6/AVG_C2-ctl1_exp14aug_rb399-slice2_2-6-8tif512-1.tif]

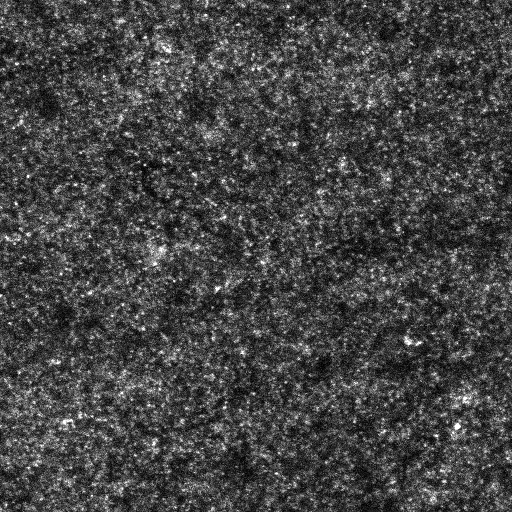

Supplement: Supplementary file 15 — Source data Fig. 6 [file 44319_2025_644_MOESM15_ESM.zip › Figure 6/6G/Ctl/Slice2_2-6/AVG_C2-ctl1_exp14aug_rb399-slice2_2-6-8tif512-2.tif]

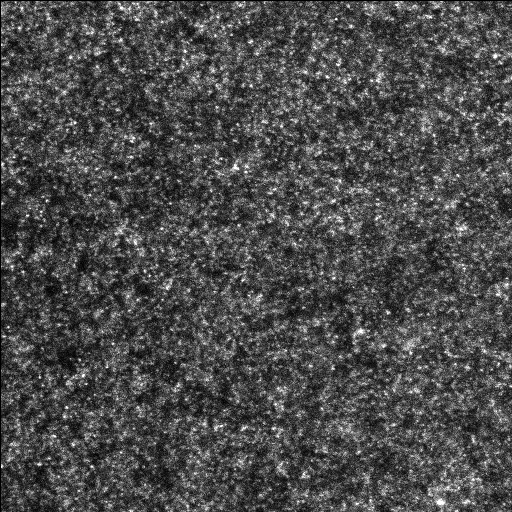

Supplement: Supplementary file 15 — Source data Fig. 6 [file 44319_2025_644_MOESM15_ESM.zip › Figure 6/6G/Ctl/Slice2_2-6/AVG_C2-ctl1_exp14aug_rb399-slice2_2-6-8tif512-3.tif]

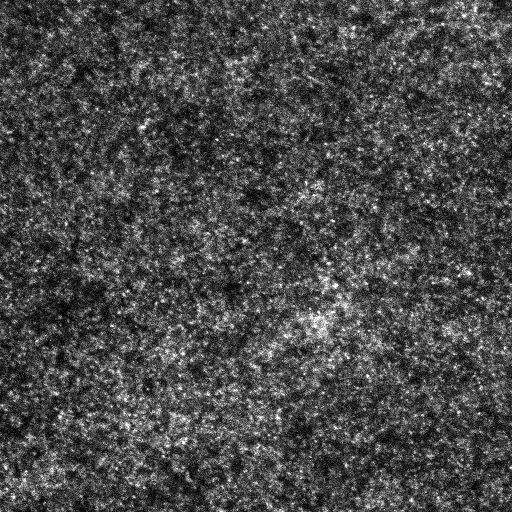

Supplement: Supplementary file 15 — Source data Fig. 6 [file 44319_2025_644_MOESM15_ESM.zip › Figure 6/6G/Ctl/Slice2_2-6/AVG_C2-ctl1_exp14aug_rb399-slice2_2-6-8tif512-4.tif]

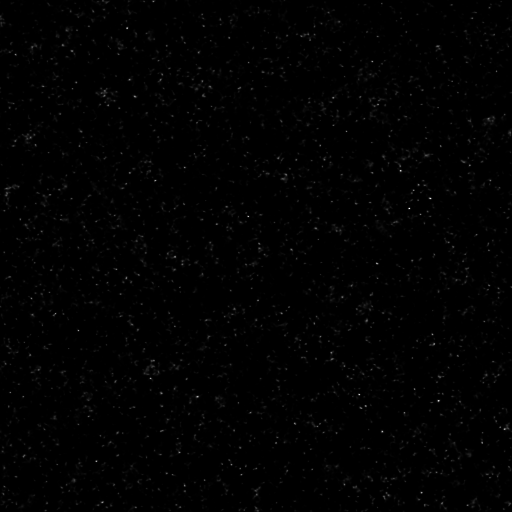

Supplement: Supplementary file 15 — Source data Fig. 6 [file 44319_2025_644_MOESM15_ESM.zip › Figure 6/6G/Ctl/Slice2_2_1-3/AVG_C2-ctl1_exp14aug_homer- slice2_2-1-3.tif512-3_deconv.tif]

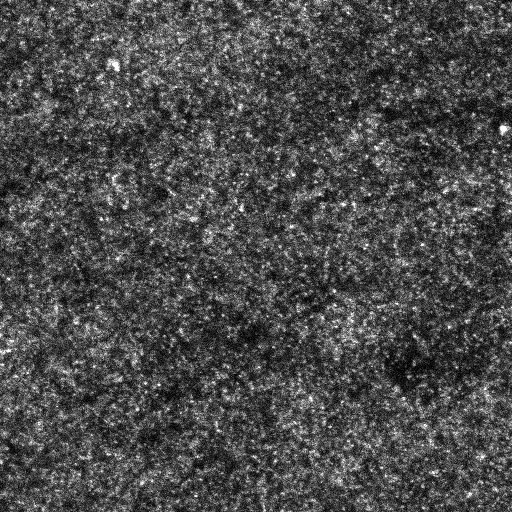

Supplement: Supplementary file 15 — Source data Fig. 6 [file 44319_2025_644_MOESM15_ESM.zip › Figure 6/6G/Ctl/Slice2_2_1-3/AVG_C2-ctl1_exp14aug_rb399- slice2_2-1-3.tif512-1.tif]

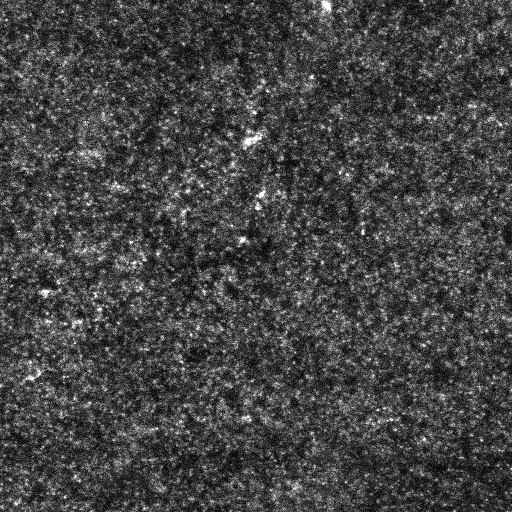

Supplement: Supplementary file 15 — Source data Fig. 6 [file 44319_2025_644_MOESM15_ESM.zip › Figure 6/6G/Ctl/Slice2_2_1-3/AVG_C2-ctl1_exp14aug_rb399- slice2_2-1-3.tif512-2.tif]

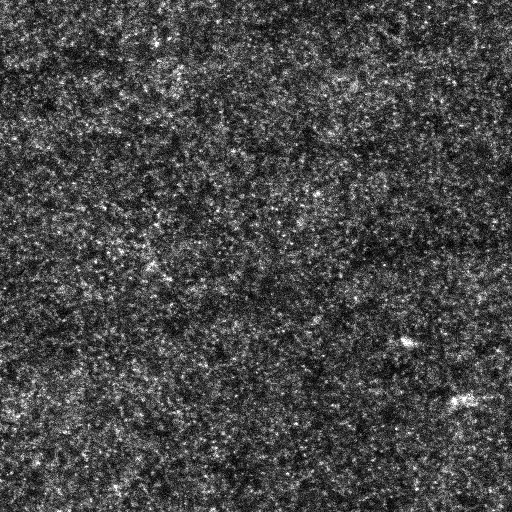

Supplement: Supplementary file 15 — Source data Fig. 6 [file 44319_2025_644_MOESM15_ESM.zip › Figure 6/6G/Ctl/Slice2_2_1-3/AVG_C2-ctl1_exp14aug_rb399- slice2_2-1-3.tif512-3.tif]

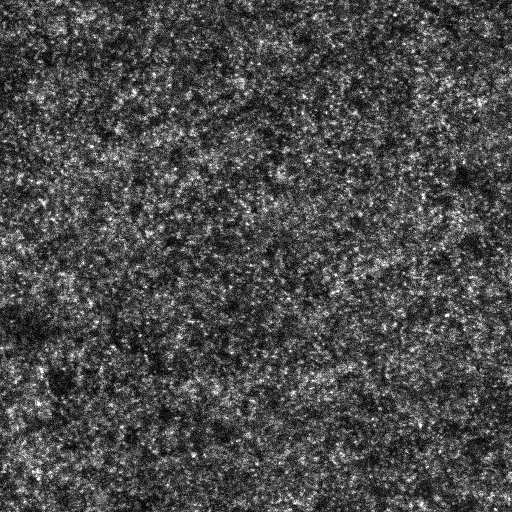

Supplement: Supplementary file 15 — Source data Fig. 6 [file 44319_2025_644_MOESM15_ESM.zip › Figure 6/6G/Ctl/Slice2_2_1-3/AVG_C2-ctl1_exp14aug_rb399- slice2_2-1-3.tif512-4.tif]

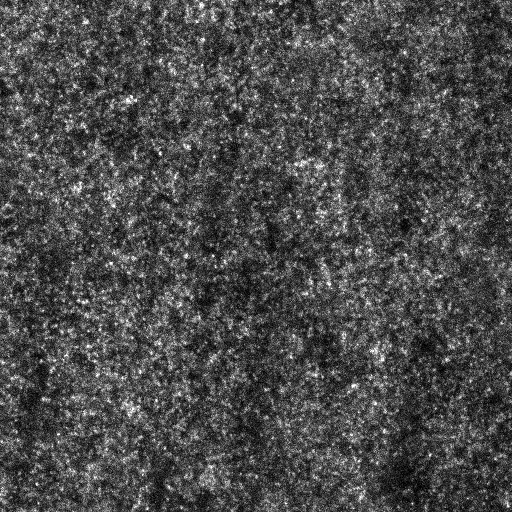

Supplement: Supplementary file 15 — Source data Fig. 6 [file 44319_2025_644_MOESM15_ESM.zip › Figure 6/6G/PFR/slice1_1-1-3/AVG_C3-pfr_exp14aug_rb399- slice1_1-1-3 512-1.tif]

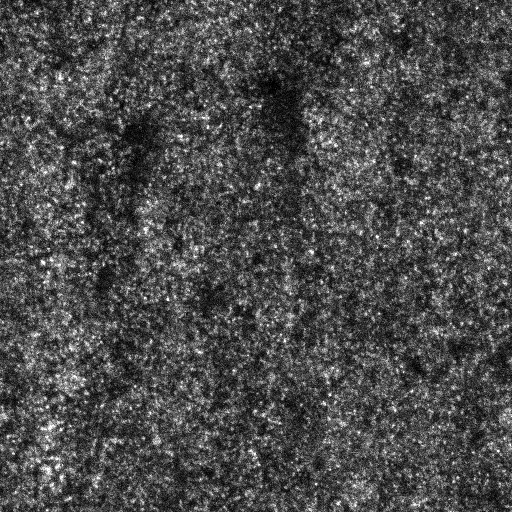

Supplement: Supplementary file 15 — Source data Fig. 6 [file 44319_2025_644_MOESM15_ESM.zip › Figure 6/6G/PFR/slice1_1-1-3/AVG_C3-pfr_exp14aug_rb399- slice1_1-1-3 512-2.tif]

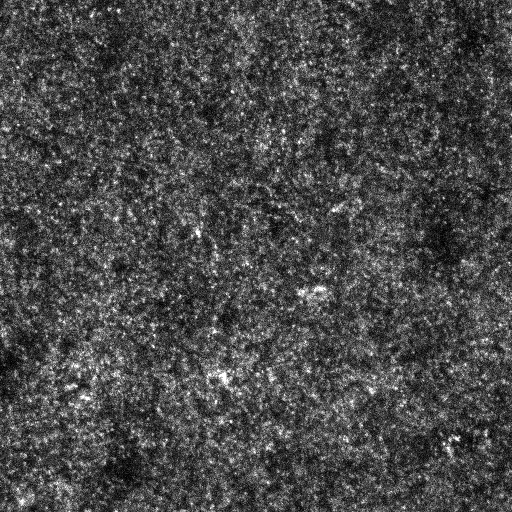

Supplement: Supplementary file 15 — Source data Fig. 6 [file 44319_2025_644_MOESM15_ESM.zip › Figure 6/6G/PFR/slice1_1-1-3/AVG_C3-pfr_exp14aug_rb399- slice1_1-1-3 512-3.tif]

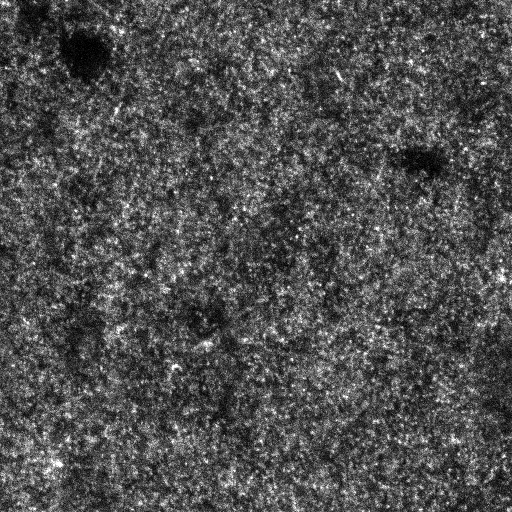

Supplement: Supplementary file 15 — Source data Fig. 6 [file 44319_2025_644_MOESM15_ESM.zip › Figure 6/6G/PFR/slice1_1-1-3/AVG_C3-pfr_exp14aug_rb399- slice1_1-1-3 512-4.tif]

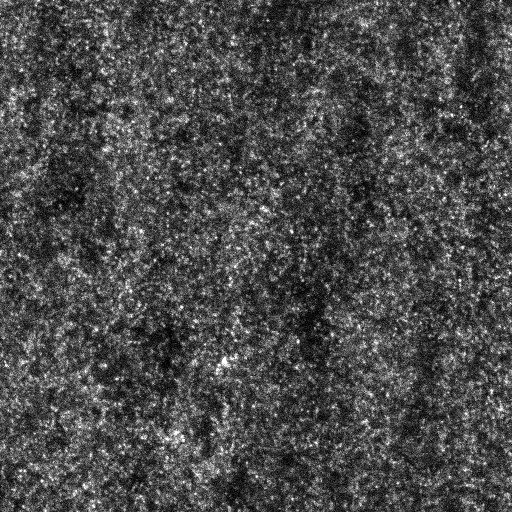

Supplement: Supplementary file 15 — Source data Fig. 6 [file 44319_2025_644_MOESM15_ESM.zip › Figure 6/6G/PFR/slice1_1-6-9/AVG_C3-pfr_exp14aug_rb399- slice1_1-6-9 512-1.tif]

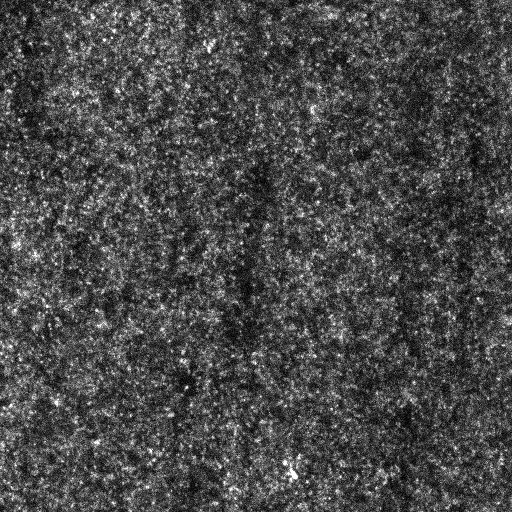

Supplement: Supplementary file 15 — Source data Fig. 6 [file 44319_2025_644_MOESM15_ESM.zip › Figure 6/6G/PFR/slice1_1-6-9/AVG_C3-pfr_exp14aug_rb399- slice1_1-6-9 512-2.tif]

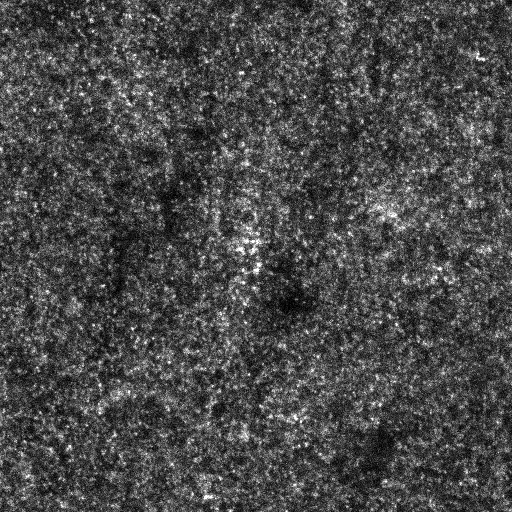

Supplement: Supplementary file 15 — Source data Fig. 6 [file 44319_2025_644_MOESM15_ESM.zip › Figure 6/6G/PFR/slice1_1-6-9/AVG_C3-pfr_exp14aug_rb399- slice1_1-6-9 512-3.tif]

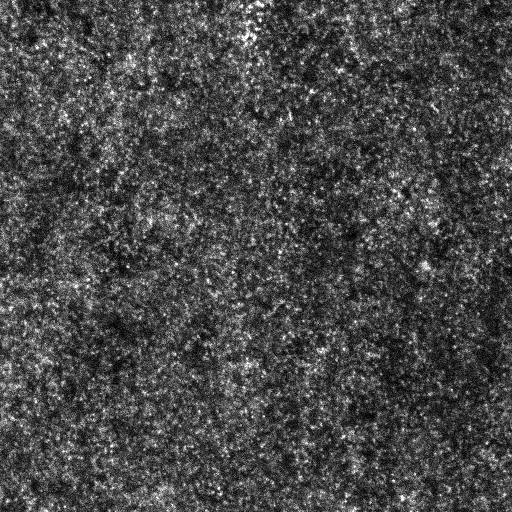

Supplement: Supplementary file 15 — Source data Fig. 6 [file 44319_2025_644_MOESM15_ESM.zip › Figure 6/6G/PFR/slice1_1-6-9/AVG_C3-pfr_exp14aug_rb399- slice1_1-6-9 512-4.tif]

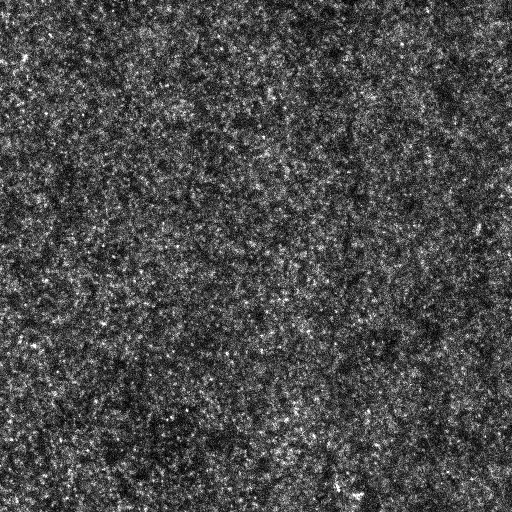

Supplement: Supplementary file 15 — Source data Fig. 6 [file 44319_2025_644_MOESM15_ESM.zip › Figure 6/6G/PFR/slice1_2-1-3/AVG_C3-pfr_exp14aug_rb399 - slice1_2-1-3 512-1.tif]

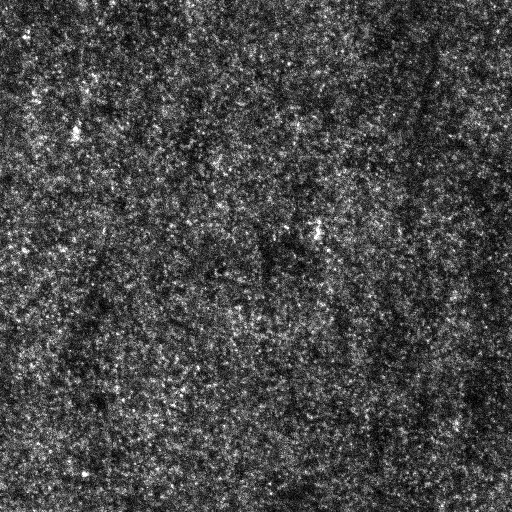

Supplement: Supplementary file 15 — Source data Fig. 6 [file 44319_2025_644_MOESM15_ESM.zip › Figure 6/6G/PFR/slice1_2-1-3/AVG_C3-pfr_exp14aug_rb399 - slice1_2-1-3 512-2.tif]

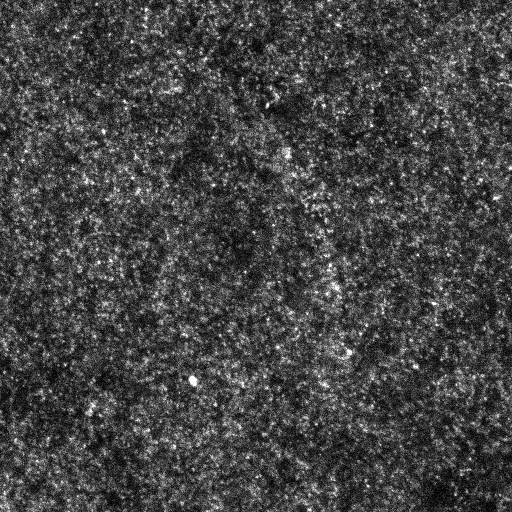

Supplement: Supplementary file 15 — Source data Fig. 6 [file 44319_2025_644_MOESM15_ESM.zip › Figure 6/6G/PFR/slice1_2-1-3/AVG_C3-pfr_exp14aug_rb399 - slice1_2-1-3 512-3.tif]

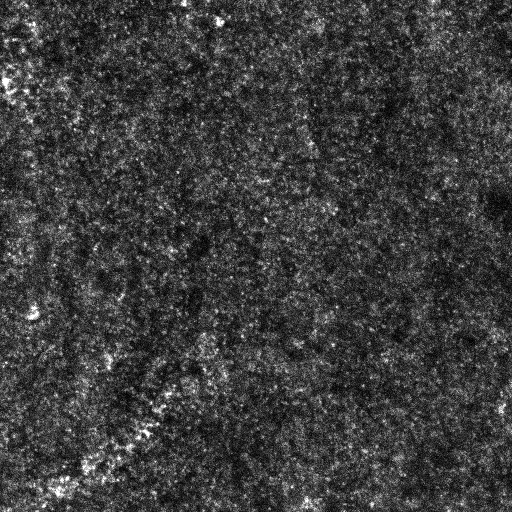

Supplement: Supplementary file 15 — Source data Fig. 6 [file 44319_2025_644_MOESM15_ESM.zip › Figure 6/6G/PFR/slice1_2-1-3/AVG_C3-pfr_exp14aug_rb399 - slice1_2-1-3 512-4.tif]

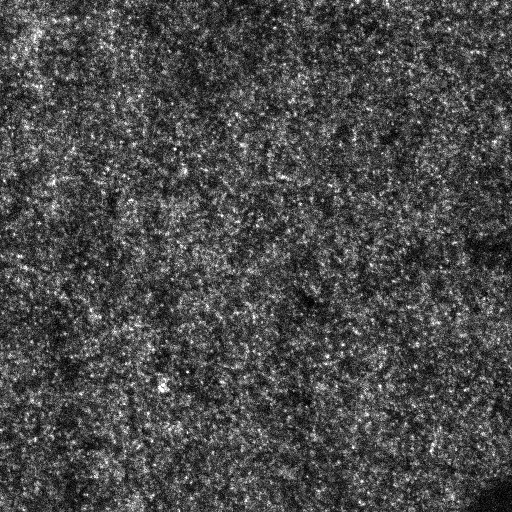

Supplement: Supplementary file 15 — Source data Fig. 6 [file 44319_2025_644_MOESM15_ESM.zip › Figure 6/6G/PFR/slice1_2-7-10/AVG_C3-pfr_exp14aug_rb399- slice1_2-7-10 512-1.tif]

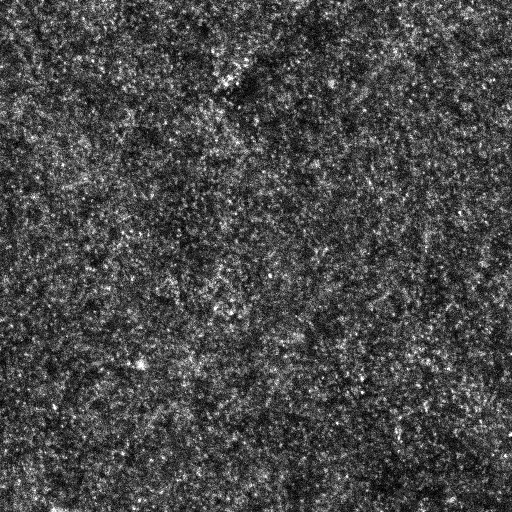

Supplement: Supplementary file 15 — Source data Fig. 6 [file 44319_2025_644_MOESM15_ESM.zip › Figure 6/6G/PFR/slice1_2-7-10/AVG_C3-pfr_exp14aug_rb399- slice1_2-7-10 512-2.tif]

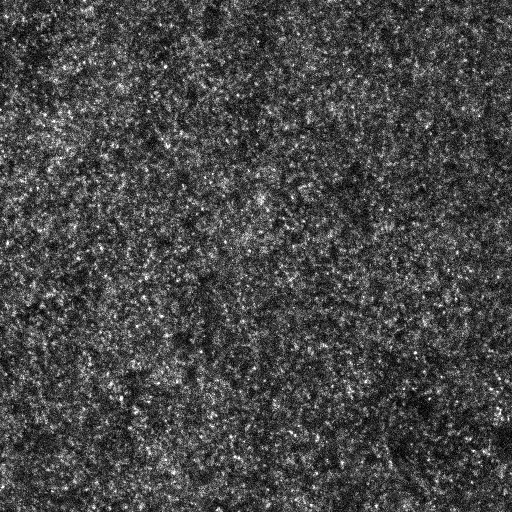

Supplement: Supplementary file 15 — Source data Fig. 6 [file 44319_2025_644_MOESM15_ESM.zip › Figure 6/6G/PFR/slice1_2-7-10/AVG_C3-pfr_exp14aug_rb399- slice1_2-7-10 512-3.tif]

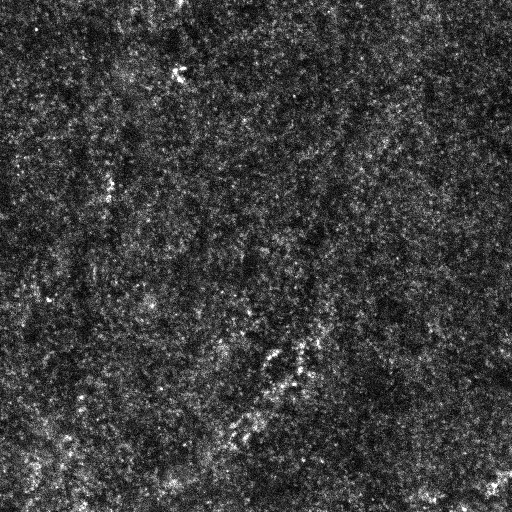

Supplement: Supplementary file 15 — Source data Fig. 6 [file 44319_2025_644_MOESM15_ESM.zip › Figure 6/6G/PFR/slice1_2-7-10/AVG_C3-pfr_exp14aug_rb399- slice1_2-7-10 512-4.tif]

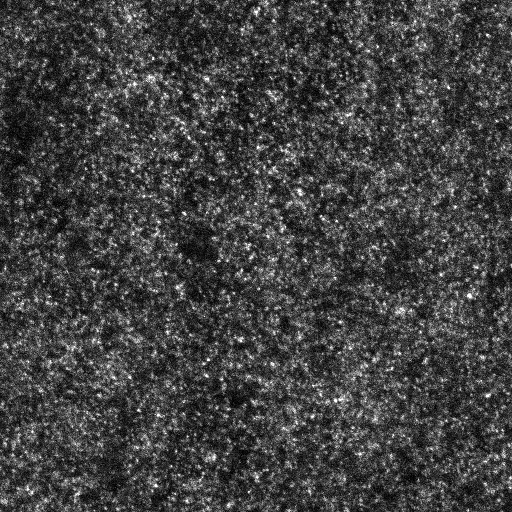

Supplement: Supplementary file 15 — Source data Fig. 6 [file 44319_2025_644_MOESM15_ESM.zip › Figure 6/6G/PFR/slice2_1-1-3/AVG_C3-pfr_exp14aug_rb399- slice2_1-1-3 512-1.tif]

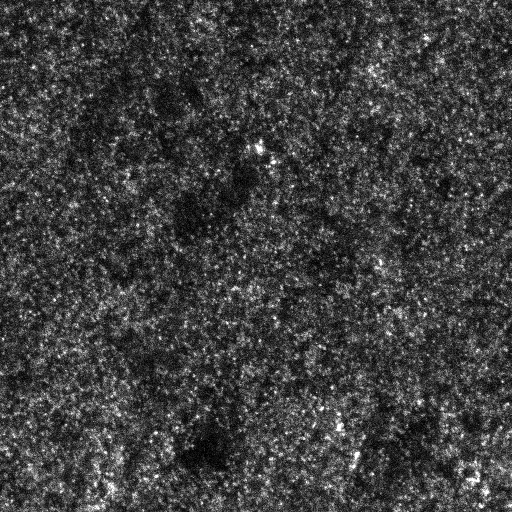

Supplement: Supplementary file 15 — Source data Fig. 6 [file 44319_2025_644_MOESM15_ESM.zip › Figure 6/6G/PFR/slice2_1-1-3/AVG_C3-pfr_exp14aug_rb399- slice2_1-1-3 512-2.tif]

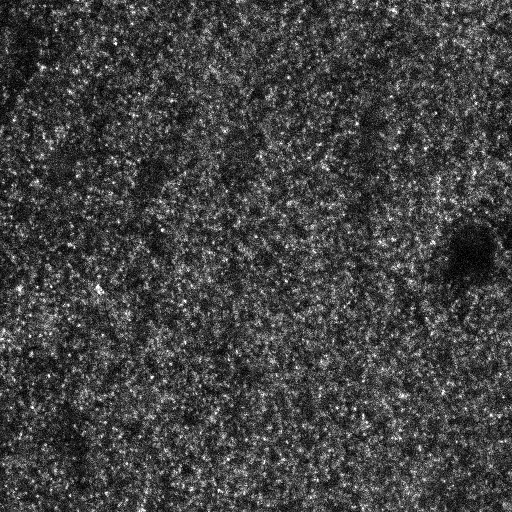

Supplement: Supplementary file 15 — Source data Fig. 6 [file 44319_2025_644_MOESM15_ESM.zip › Figure 6/6G/PFR/slice2_1-1-3/AVG_C3-pfr_exp14aug_rb399- slice2_1-1-3 512-3.tif]

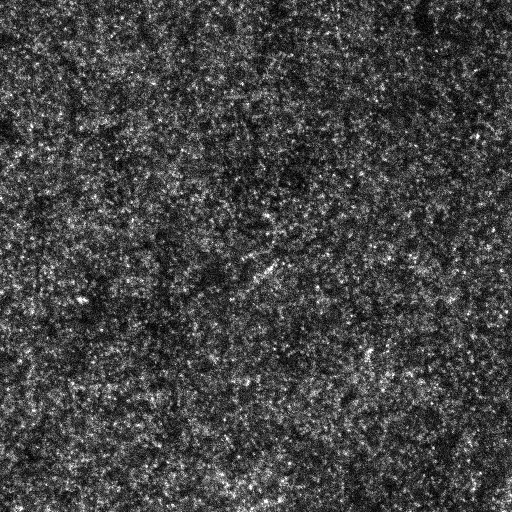

Supplement: Supplementary file 15 — Source data Fig. 6 [file 44319_2025_644_MOESM15_ESM.zip › Figure 6/6G/PFR/slice2_1-1-3/AVG_C3-pfr_exp14aug_rb399- slice2_1-1-3 512-4.tif]

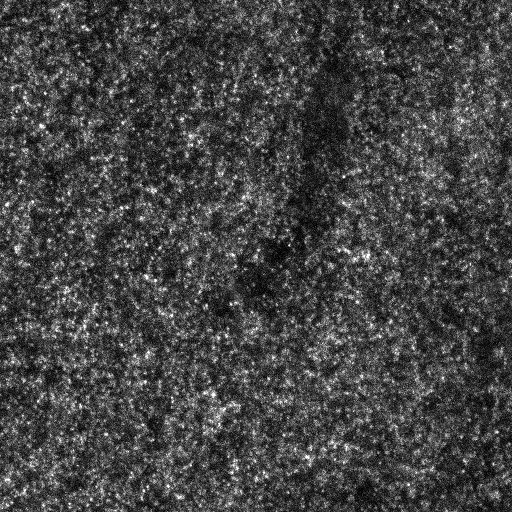

Supplement: Supplementary file 15 — Source data Fig. 6 [file 44319_2025_644_MOESM15_ESM.zip › Figure 6/6G/PFR/slice2_1-6-9/AVG_C3-pfr_exp14aug_rb399- slice2_1-6-9 512-1.tif]

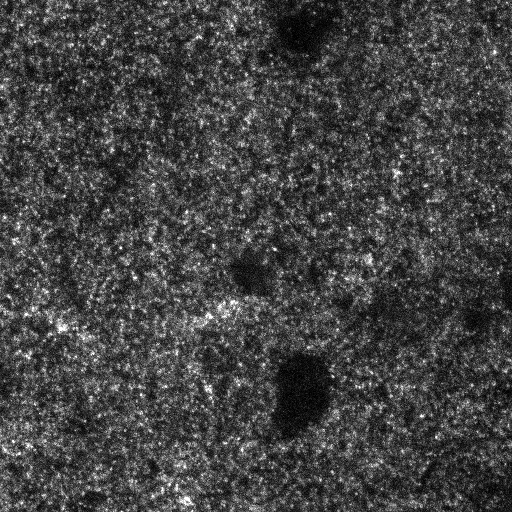

Supplement: Supplementary file 15 — Source data Fig. 6 [file 44319_2025_644_MOESM15_ESM.zip › Figure 6/6G/PFR/slice2_1-6-9/AVG_C3-pfr_exp14aug_rb399- slice2_1-6-9 512-2.tif]

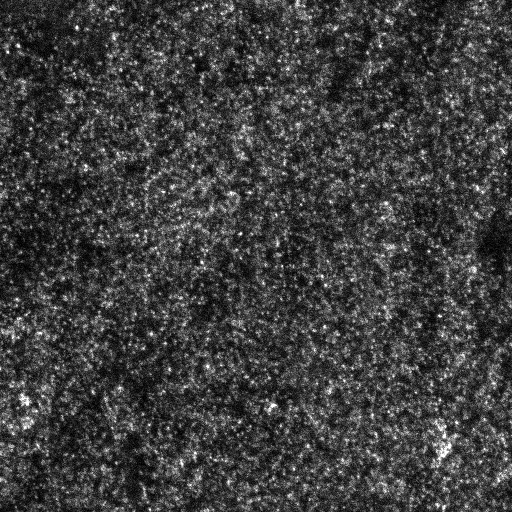

Supplement: Supplementary file 15 — Source data Fig. 6 [file 44319_2025_644_MOESM15_ESM.zip › Figure 6/6G/PFR/slice2_1-6-9/AVG_C3-pfr_exp14aug_rb399- slice2_1-6-9 512-3.tif]

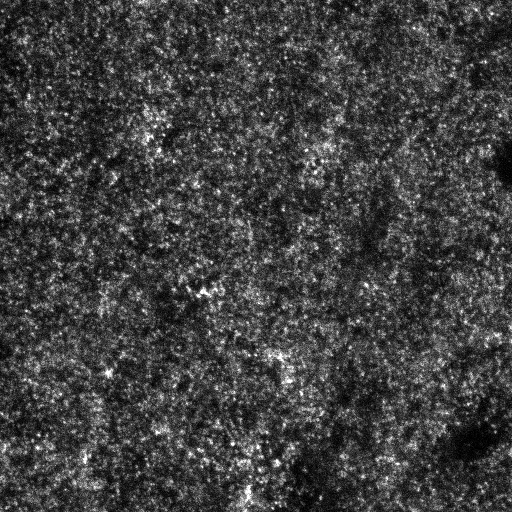

Supplement: Supplementary file 15 — Source data Fig. 6 [file 44319_2025_644_MOESM15_ESM.zip › Figure 6/6G/PFR/slice2_1-6-9/AVG_C3-pfr_exp14aug_rb399- slice2_1-6-9 512-4.tif]

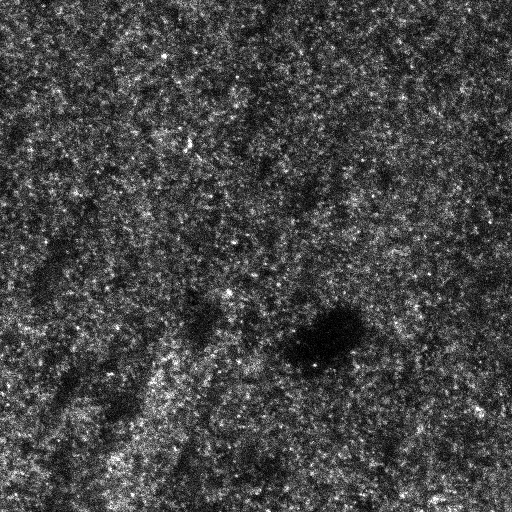

Supplement: Supplementary file 15 — Source data Fig. 6 [file 44319_2025_644_MOESM15_ESM.zip › Figure 6/6G/PFR/slice3_1-1-1/AVG_C3-pfr1_exp14augrb399- slice3_1-1-1.tif512-2]

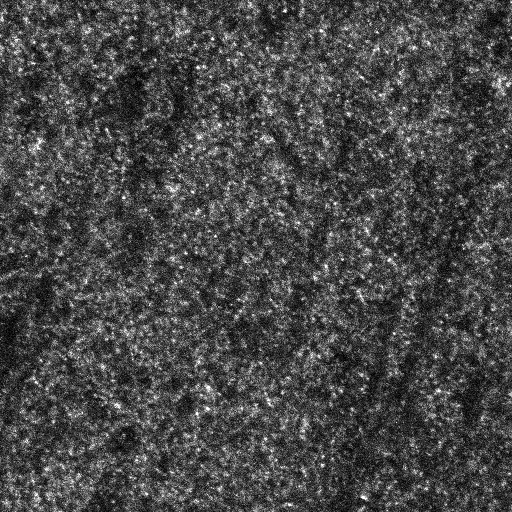

Supplement: Supplementary file 15 — Source data Fig. 6 [file 44319_2025_644_MOESM15_ESM.zip › Figure 6/6G/PFR/slice3_1-1-1/AVG_C3-pfr1_exp14aug_rb399 slice3_1-1-1.tif512-3]

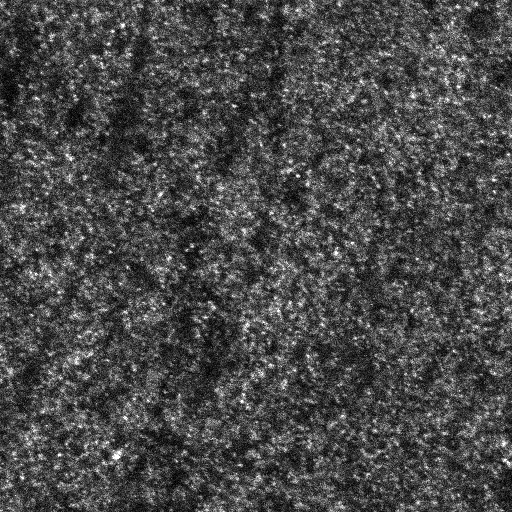

Supplement: Supplementary file 15 — Source data Fig. 6 [file 44319_2025_644_MOESM15_ESM.zip › Figure 6/6G/PFR/slice3_1-1-1/AVG_C3-pfr1_exp14aug_rb399- slice3_1-1-1.tif-4]

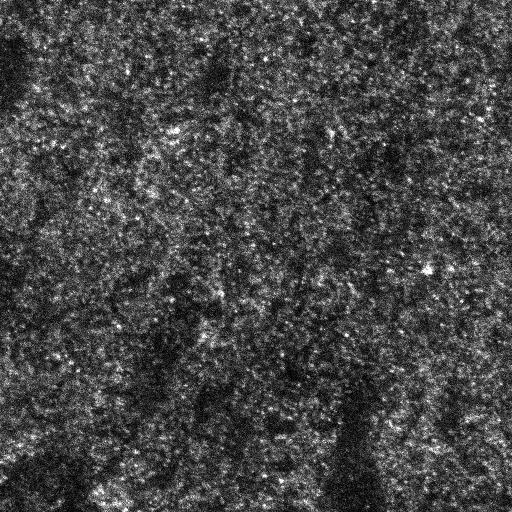

Supplement: Supplementary file 15 — Source data Fig. 6 [file 44319_2025_644_MOESM15_ESM.zip › Figure 6/6G/PFR/slice3_1-1-1/AVG_C3-pfr1_exp14aug_rb399- slice3_1-1-1.tif-512-1]
