# Supplementary material for: Activity-dependent extracellular proteolytic cascade cleaves the ECM component brevican to promote structural plasticity
Source: EMBO Rep. 2025 Nov 19;27(1):163–85. doi: 10.1038/s44319-025-00644-w (PMC12796228; doi:10.1038/s44319-025-00644-w)
Supplement: Supplementary file 11 — Source data Fig. 2 [file 44319_2025_644_MOESM11_ESM.zip › Figure 2/figure 2A neo.pdf]

neo anti BC

1 Ctl
2 PFR
3 PFR & Furini II

53 kDa

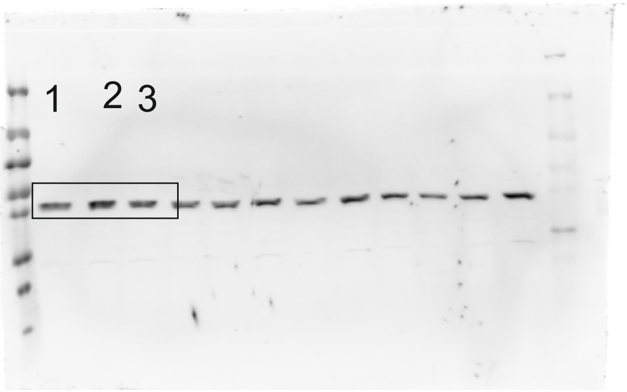

neo anti BC

neo anti BC

1 Ctl
2 PFR
3 PFR & Furini I

1 2 3

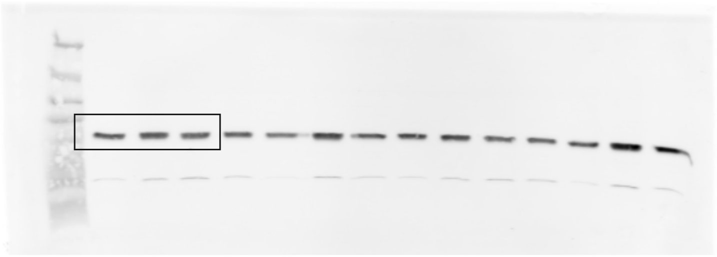

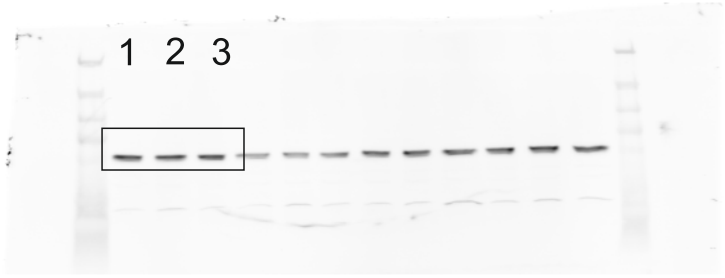

neo anti BC

1 Ctl
2 PFR
3 PFR & PCI
