# Supplementary figures and images for: Activity-dependent extracellular proteolytic cascade cleaves the ECM component brevican to promote structural plasticity (part 2 of 3)
Source: EMBO Rep. 2025 Nov 19;27(1):163–85. doi: 10.1038/s44319-025-00644-w (PMC12796228; doi:10.1038/s44319-025-00644-w)

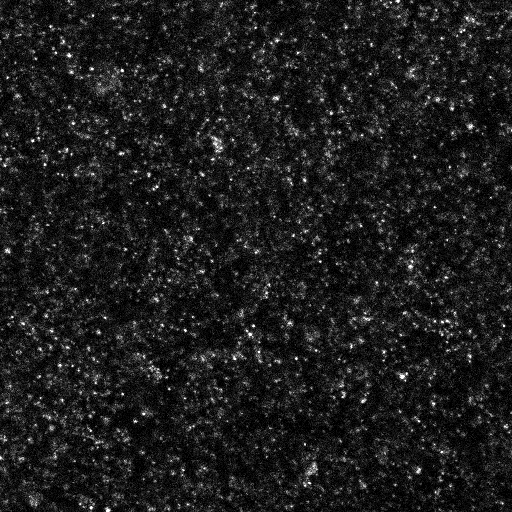

Supplement: Supplementary file 15 — Source data Fig. 6 [file 44319_2025_644_MOESM15_ESM.zip › Figure 6/6I/Ctl/Ctl3_2-1-1-3/AVG_C2-ctl1_exp14aug_homer- ctl3_2-1-1-3.tif512-1.tif]

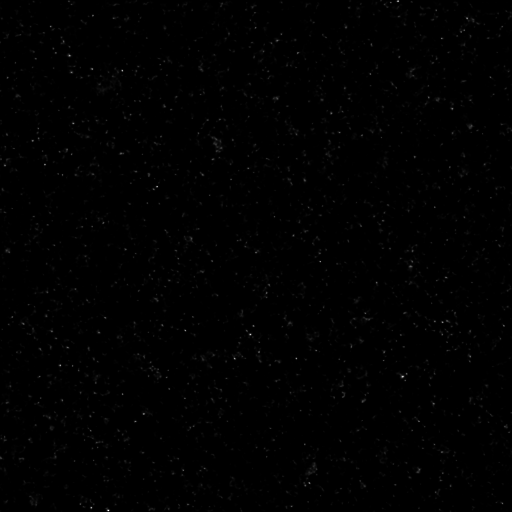

Supplement: Supplementary file 15 — Source data Fig. 6 [file 44319_2025_644_MOESM15_ESM.zip › Figure 6/6I/Ctl/Ctl3_2-1-1-3/AVG_C2-ctl1_exp14aug_homer- ctl3_2-1-1-3.tif512-1_deconv.tif]

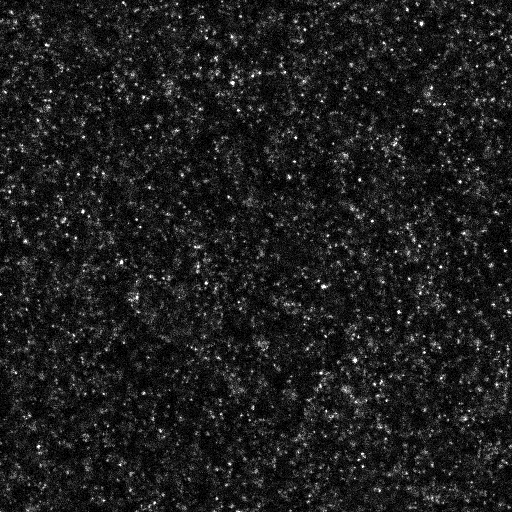

Supplement: Supplementary file 15 — Source data Fig. 6 [file 44319_2025_644_MOESM15_ESM.zip › Figure 6/6I/Ctl/Ctl3_2-1-1-3/AVG_C2-ctl1_exp14aug_homer- ctl3_2-1-1-3.tif512-2.tif]

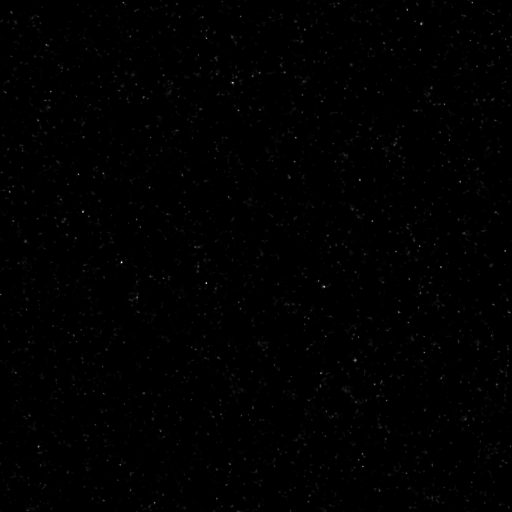

Supplement: Supplementary file 15 — Source data Fig. 6 [file 44319_2025_644_MOESM15_ESM.zip › Figure 6/6I/Ctl/Ctl3_2-1-1-3/AVG_C2-ctl1_exp14aug_homer- ctl3_2-1-1-3.tif512-2_deconv.tif]

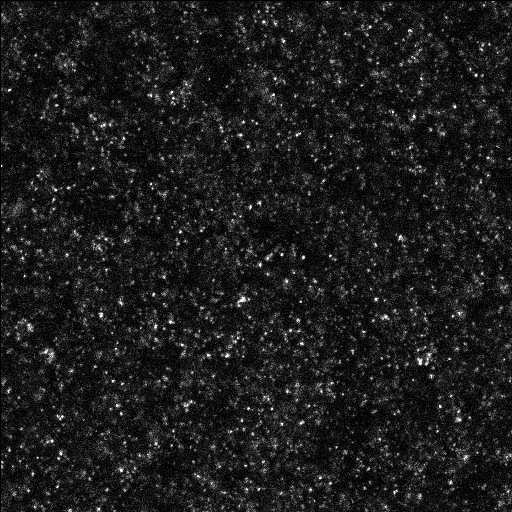

Supplement: Supplementary file 15 — Source data Fig. 6 [file 44319_2025_644_MOESM15_ESM.zip › Figure 6/6I/Ctl/Ctl3_2-1-1-3/AVG_C2-ctl1_exp14aug_homer- ctl3_2-1-1-3.tif512-3.tif]

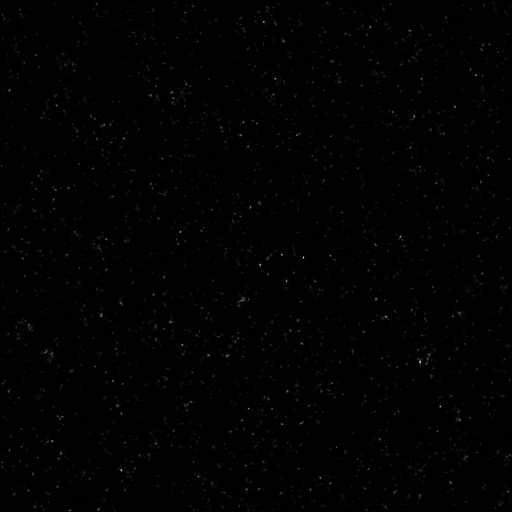

Supplement: Supplementary file 15 — Source data Fig. 6 [file 44319_2025_644_MOESM15_ESM.zip › Figure 6/6I/Ctl/Ctl3_2-1-1-3/AVG_C2-ctl1_exp14aug_homer- ctl3_2-1-1-3.tif512-3_deconv.tif]

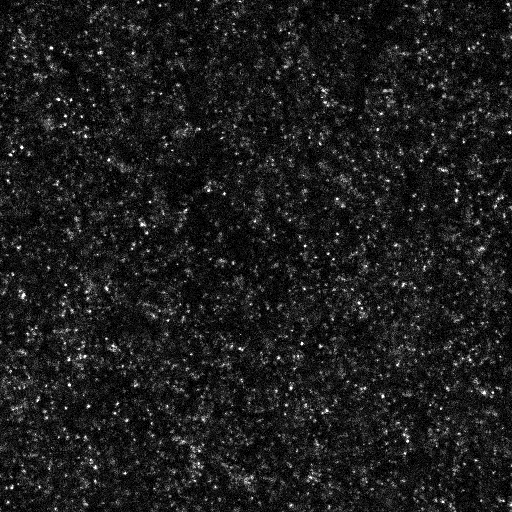

Supplement: Supplementary file 15 — Source data Fig. 6 [file 44319_2025_644_MOESM15_ESM.zip › Figure 6/6I/Ctl/Ctl3_2-1-1-3/AVG_C2-ctl1_exp14aug_homer- ctl3_2-1-1-3.tif512-4.tif]

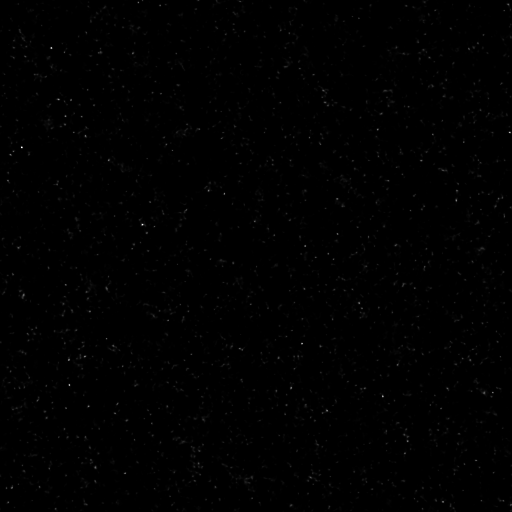

Supplement: Supplementary file 15 — Source data Fig. 6 [file 44319_2025_644_MOESM15_ESM.zip › Figure 6/6I/Ctl/Ctl3_2-1-1-3/AVG_C2-ctl1_exp14aug_homer- ctl3_2-1-1-3.tif512-4_deconv.tif]

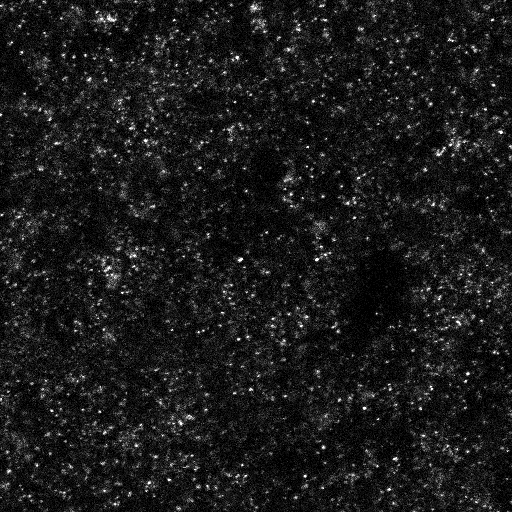

Supplement: Supplementary file 15 — Source data Fig. 6 [file 44319_2025_644_MOESM15_ESM.zip › Figure 6/6I/Ctl/Slice1_3-6-1/AVG_C2-ctl_exp14aug_homer-slice1_3-6-1.tif512-1.tif]

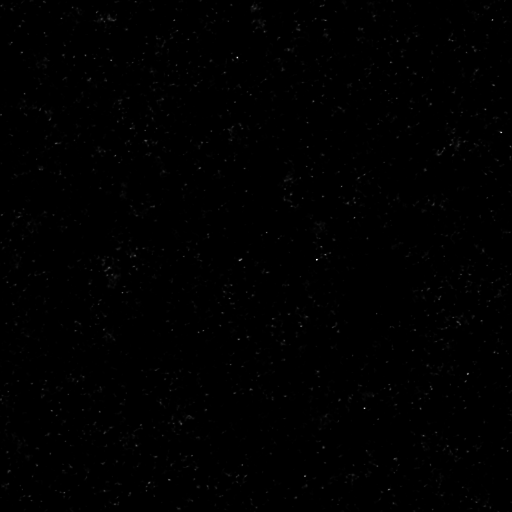

Supplement: Supplementary file 15 — Source data Fig. 6 [file 44319_2025_644_MOESM15_ESM.zip › Figure 6/6I/Ctl/Slice1_3-6-1/AVG_C2-ctl_exp14aug_homer-slice1_3-6-1.tif512-1_deconv.tif]

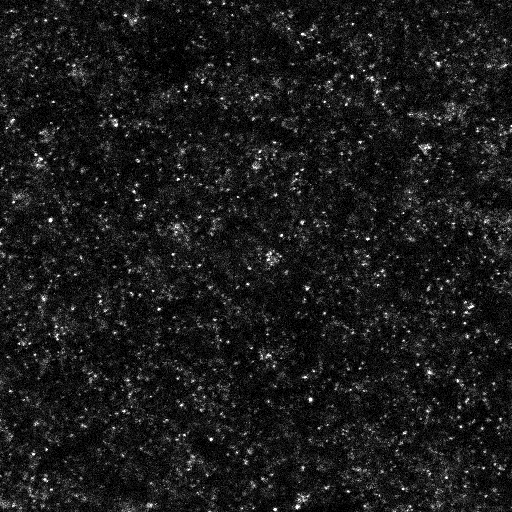

Supplement: Supplementary file 15 — Source data Fig. 6 [file 44319_2025_644_MOESM15_ESM.zip › Figure 6/6I/Ctl/Slice1_3-6-1/AVG_C2-ctl_exp14aug_homer-slice1_3-6-1.tif512-2.tif]

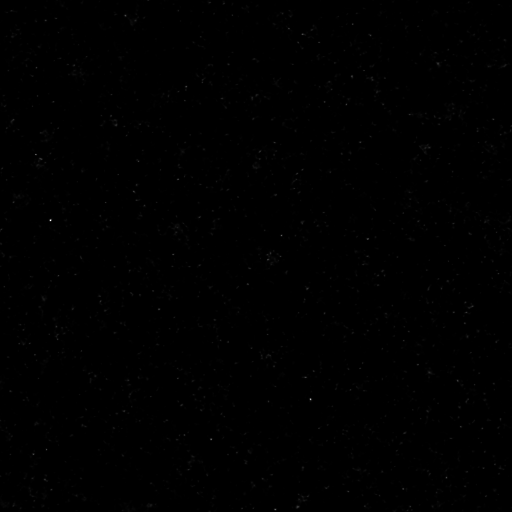

Supplement: Supplementary file 15 — Source data Fig. 6 [file 44319_2025_644_MOESM15_ESM.zip › Figure 6/6I/Ctl/Slice1_3-6-1/AVG_C2-ctl_exp14aug_homer-slice1_3-6-1.tif512-2_deconv.tif]

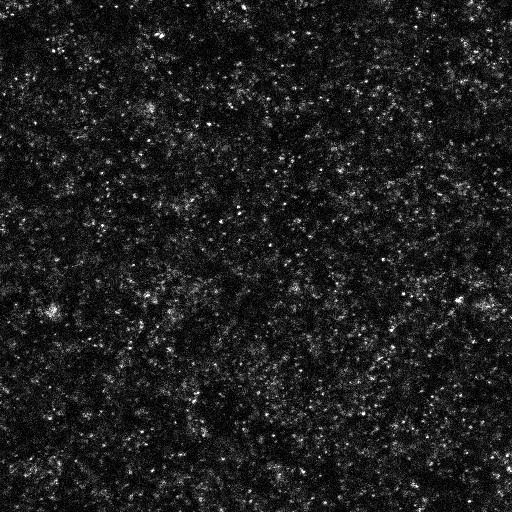

Supplement: Supplementary file 15 — Source data Fig. 6 [file 44319_2025_644_MOESM15_ESM.zip › Figure 6/6I/Ctl/Slice1_3-6-1/AVG_C2-ctl_exp14aug_homer-slice1_3-6-1.tif512-3.tif]

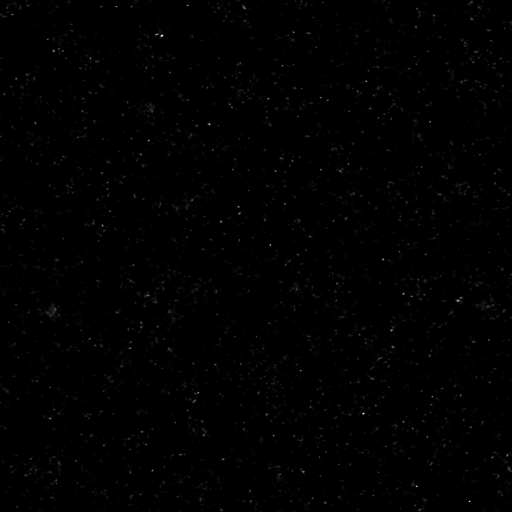

Supplement: Supplementary file 15 — Source data Fig. 6 [file 44319_2025_644_MOESM15_ESM.zip › Figure 6/6I/Ctl/Slice1_3-6-1/AVG_C2-ctl_exp14aug_homer-slice1_3-6-1.tif512-3_deconv.tif]

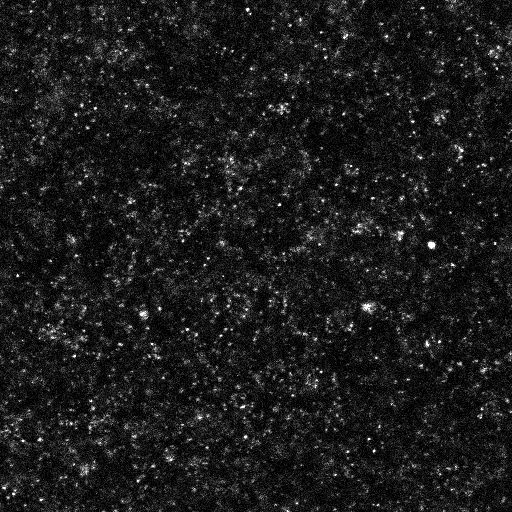

Supplement: Supplementary file 15 — Source data Fig. 6 [file 44319_2025_644_MOESM15_ESM.zip › Figure 6/6I/Ctl/Slice1_3-6-1/AVG_C2-ctl_exp14aug_homer-slice1_3-6-1.tif512-4.tif]

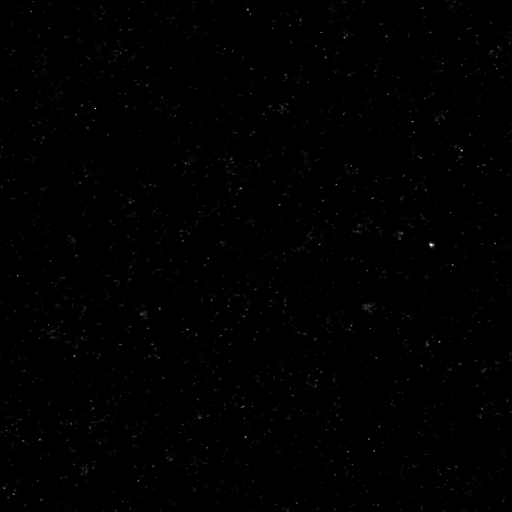

Supplement: Supplementary file 15 — Source data Fig. 6 [file 44319_2025_644_MOESM15_ESM.zip › Figure 6/6I/Ctl/Slice1_3-6-1/AVG_C2-ctl_exp14aug_homer-slice1_3-6-1.tif512-4_deconv.tif]

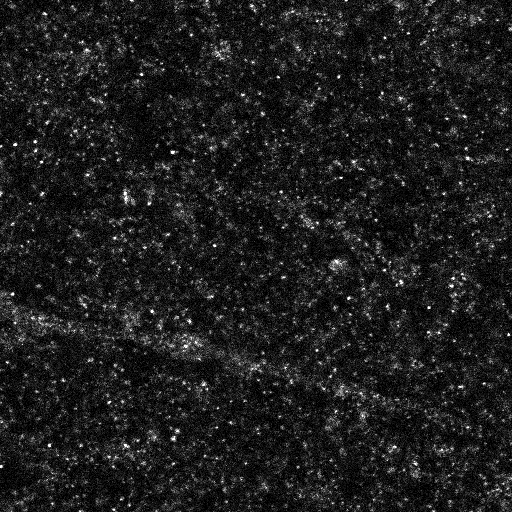

Supplement: Supplementary file 15 — Source data Fig. 6 [file 44319_2025_644_MOESM15_ESM.zip › Figure 6/6I/Ctl/Slice1_3-6-8/AVG_C2-ctl_exp14aug_homer-slice1_3-6-8.tif512-1.tif]

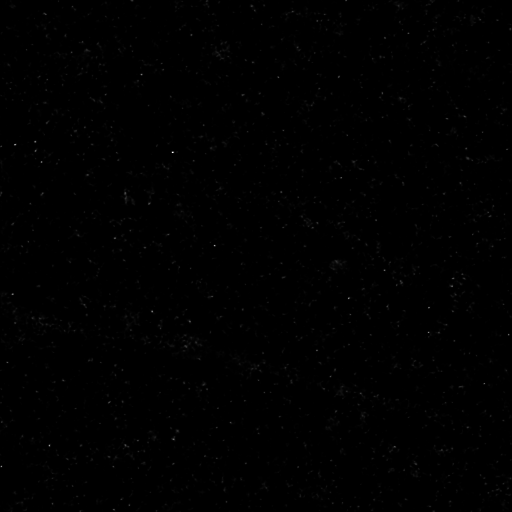

Supplement: Supplementary file 15 — Source data Fig. 6 [file 44319_2025_644_MOESM15_ESM.zip › Figure 6/6I/Ctl/Slice1_3-6-8/AVG_C2-ctl_exp14aug_homer-slice1_3-6-8.tif512-1_deconv.tif]

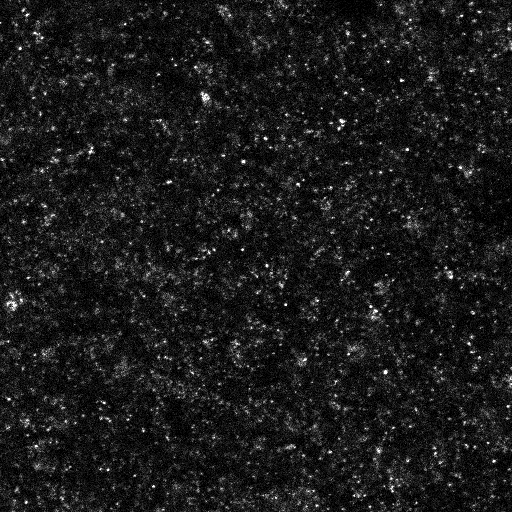

Supplement: Supplementary file 15 — Source data Fig. 6 [file 44319_2025_644_MOESM15_ESM.zip › Figure 6/6I/Ctl/Slice1_3-6-8/AVG_C2-ctl_exp14aug_homer-slice1_3-6-8.tif512-2.tif]

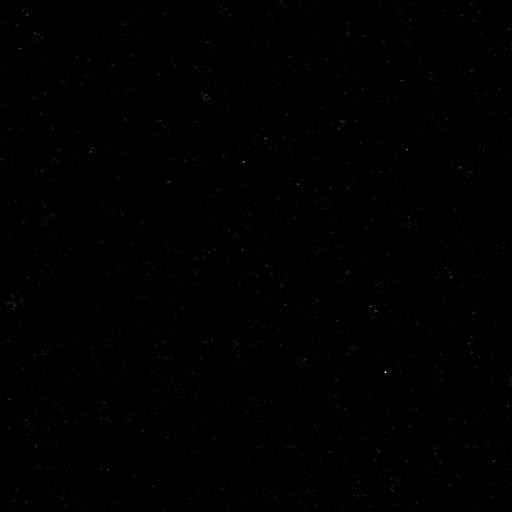

Supplement: Supplementary file 15 — Source data Fig. 6 [file 44319_2025_644_MOESM15_ESM.zip › Figure 6/6I/Ctl/Slice1_3-6-8/AVG_C2-ctl_exp14aug_homer-slice1_3-6-8.tif512-2_deconv.tif]

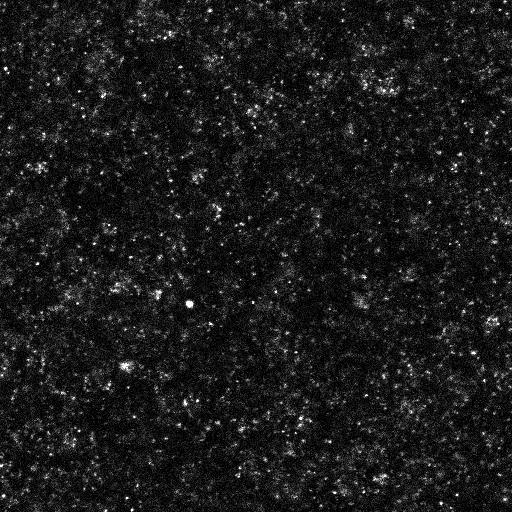

Supplement: Supplementary file 15 — Source data Fig. 6 [file 44319_2025_644_MOESM15_ESM.zip › Figure 6/6I/Ctl/Slice1_3-6-8/AVG_C2-ctl_exp14aug_homer-slice1_3-6-8.tif512-3.tif]

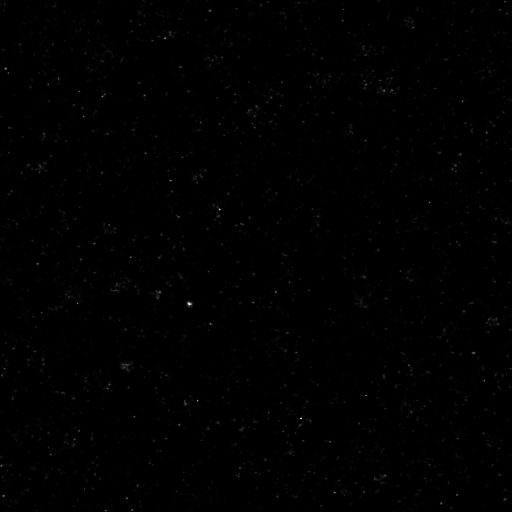

Supplement: Supplementary file 15 — Source data Fig. 6 [file 44319_2025_644_MOESM15_ESM.zip › Figure 6/6I/Ctl/Slice1_3-6-8/AVG_C2-ctl_exp14aug_homer-slice1_3-6-8.tif512-3_deconv.tif]

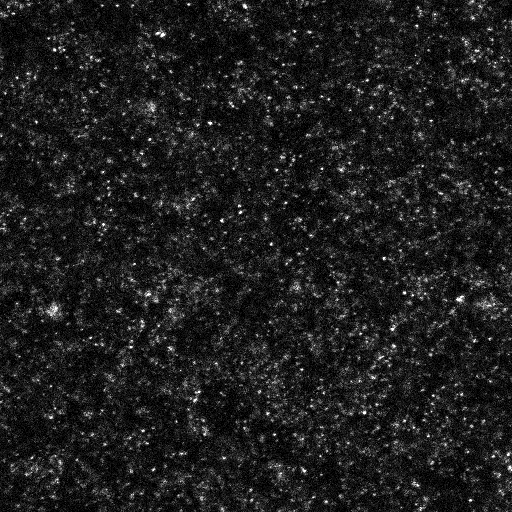

Supplement: Supplementary file 15 — Source data Fig. 6 [file 44319_2025_644_MOESM15_ESM.zip › Figure 6/6I/Ctl/Slice1_3-6-8/AVG_C2-ctl_exp14aug_homer-slice1_3-6-8.tif512-4.tif]

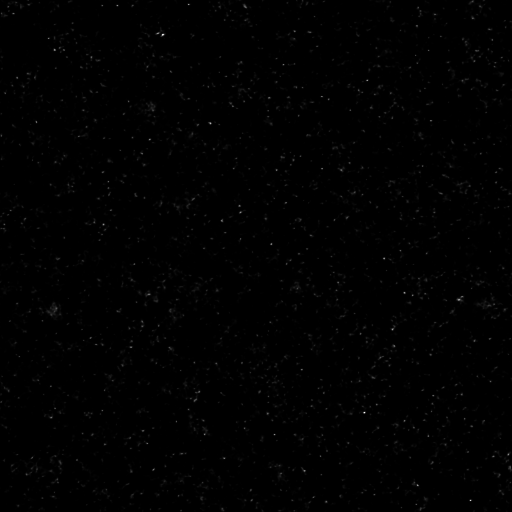

Supplement: Supplementary file 15 — Source data Fig. 6 [file 44319_2025_644_MOESM15_ESM.zip › Figure 6/6I/Ctl/Slice1_3-6-8/AVG_C2-ctl_exp14aug_homer-slice1_3-6-8.tif512-4_deconv.tif]

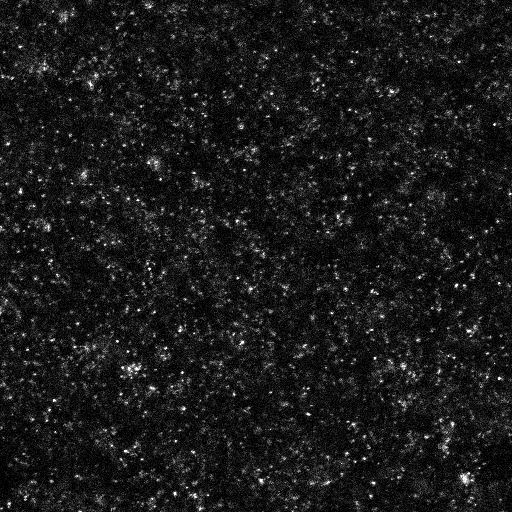

Supplement: Supplementary file 15 — Source data Fig. 6 [file 44319_2025_644_MOESM15_ESM.zip › Figure 6/6I/Ctl/Slice1_3-6-8/AVG_C2-ctl_exp14aug_homer-slice1_3-6-8.tif512-5.tif]

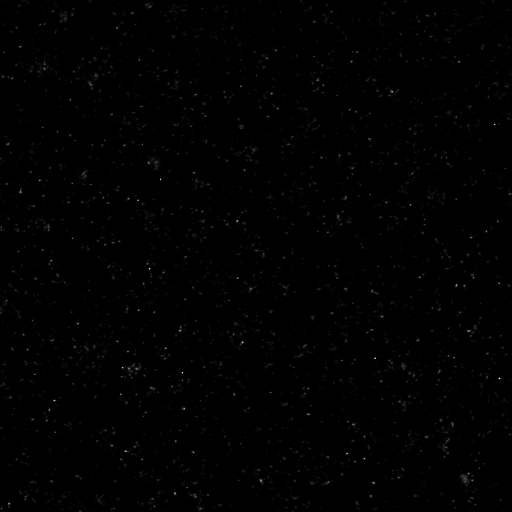

Supplement: Supplementary file 15 — Source data Fig. 6 [file 44319_2025_644_MOESM15_ESM.zip › Figure 6/6I/Ctl/Slice1_3-6-8/AVG_C2-ctl_exp14aug_homer-slice1_3-6-8.tif512-5_deconv.tif]

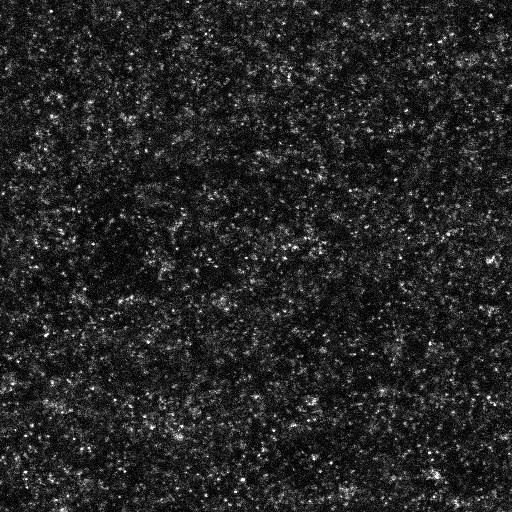

Supplement: Supplementary file 15 — Source data Fig. 6 [file 44319_2025_644_MOESM15_ESM.zip › Figure 6/6I/Ctl/Slice2_1-1-3/AVG_C2-ctl1_exp14aug_homer- slice2_1-1-3.tif512-2.tif]

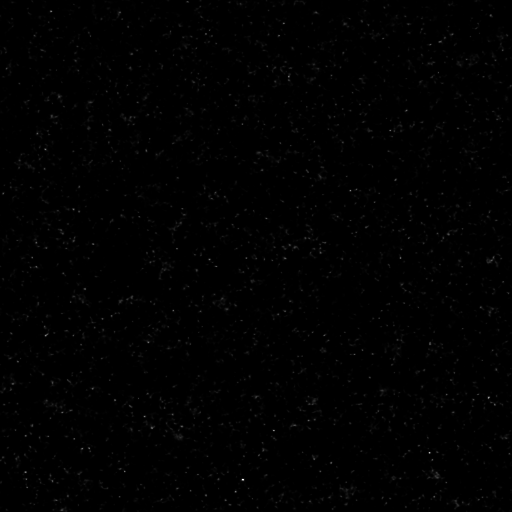

Supplement: Supplementary file 15 — Source data Fig. 6 [file 44319_2025_644_MOESM15_ESM.zip › Figure 6/6I/Ctl/Slice2_1-1-3/AVG_C2-ctl1_exp14aug_homer- slice2_1-1-3.tif512-2_deconv.tif]

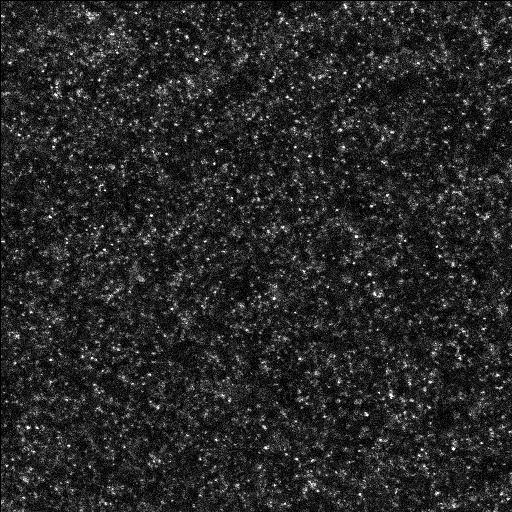

Supplement: Supplementary file 15 — Source data Fig. 6 [file 44319_2025_644_MOESM15_ESM.zip › Figure 6/6I/Ctl/Slice2_1-1-3/AVG_C2-ctl1_exp14aug_homer- slice2_1-1-3.tif512-3.tif]

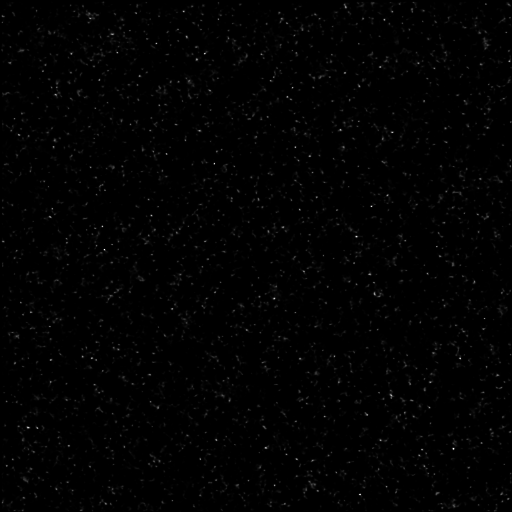

Supplement: Supplementary file 15 — Source data Fig. 6 [file 44319_2025_644_MOESM15_ESM.zip › Figure 6/6I/Ctl/Slice2_1-1-3/AVG_C2-ctl1_exp14aug_homer- slice2_1-1-3.tif512-3_deconv.tif]

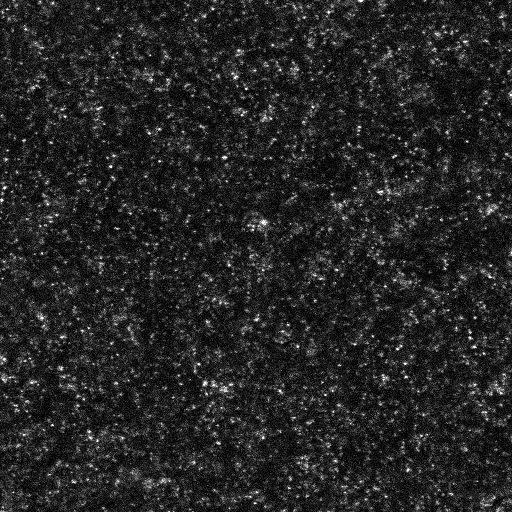

Supplement: Supplementary file 15 — Source data Fig. 6 [file 44319_2025_644_MOESM15_ESM.zip › Figure 6/6I/Ctl/Slice2_1-1-3/AVG_C2-ctl1_exp14aug_homer- slice2_1-1-3.tif512-4.tif]

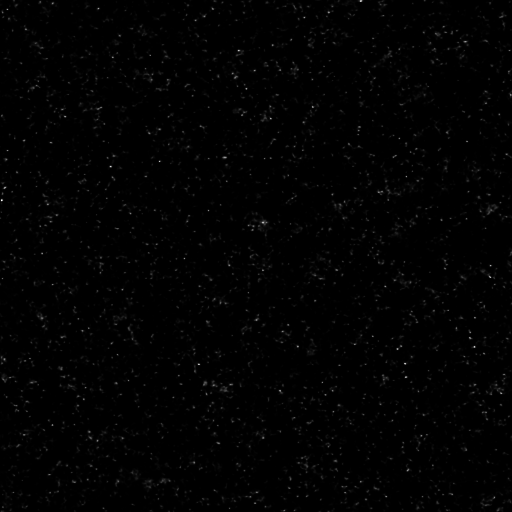

Supplement: Supplementary file 15 — Source data Fig. 6 [file 44319_2025_644_MOESM15_ESM.zip › Figure 6/6I/Ctl/Slice2_1-1-3/AVG_C2-ctl1_exp14aug_homer- slice2_1-1-3.tif512-4_deconv.tif]

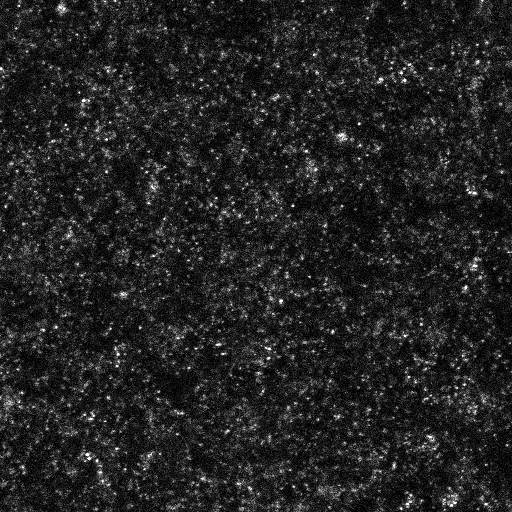

Supplement: Supplementary file 15 — Source data Fig. 6 [file 44319_2025_644_MOESM15_ESM.zip › Figure 6/6I/Ctl/Slice2_1-1-3/AVG_C2-ctl1_exp14aug_homer-slice2_1-1-3.tif512-1.tif]

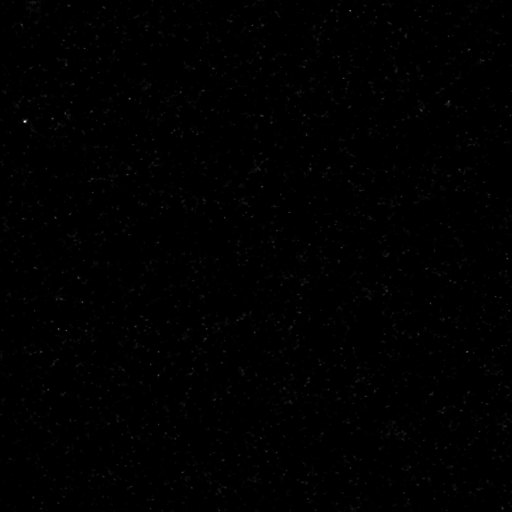

Supplement: Supplementary file 15 — Source data Fig. 6 [file 44319_2025_644_MOESM15_ESM.zip › Figure 6/6I/Ctl/Slice2_1-6-9/AVG_C3-ctl1_exp14aug_homer- slice2_1-6-9.tif512-1_deconv.tif]

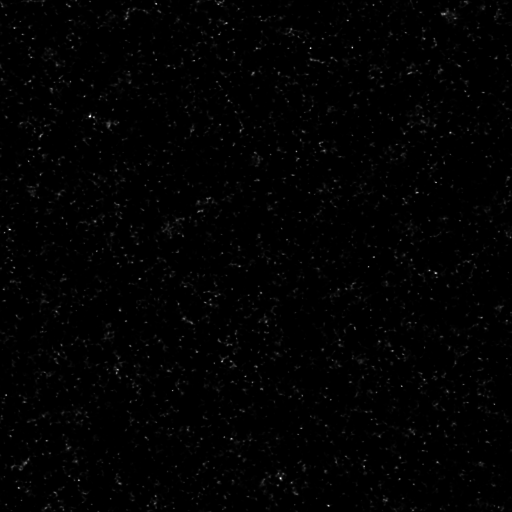

Supplement: Supplementary file 15 — Source data Fig. 6 [file 44319_2025_644_MOESM15_ESM.zip › Figure 6/6I/Ctl/Slice2_1-6-9/AVG_C3-ctl1_exp14aug_homer- slice2_1-6-9.tif512-2_deconv.tif]

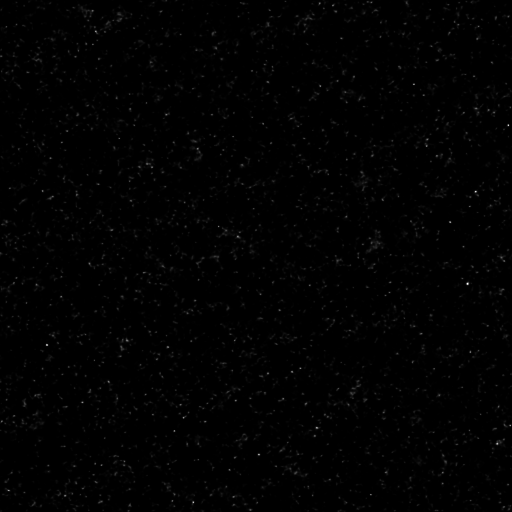

Supplement: Supplementary file 15 — Source data Fig. 6 [file 44319_2025_644_MOESM15_ESM.zip › Figure 6/6I/Ctl/Slice2_1-6-9/AVG_C3-ctl1_exp14aug_homer- slice2_1-6-9.tif512-4_deconv.tif]

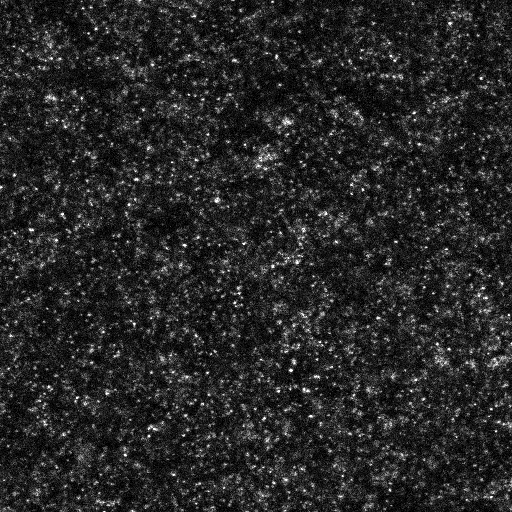

Supplement: Supplementary file 15 — Source data Fig. 6 [file 44319_2025_644_MOESM15_ESM.zip › Figure 6/6I/Ctl/Slice2_2-6/AVG_C2-ctl1_exp14aug_homer- slice2_2-6-8tif512-1.tif]

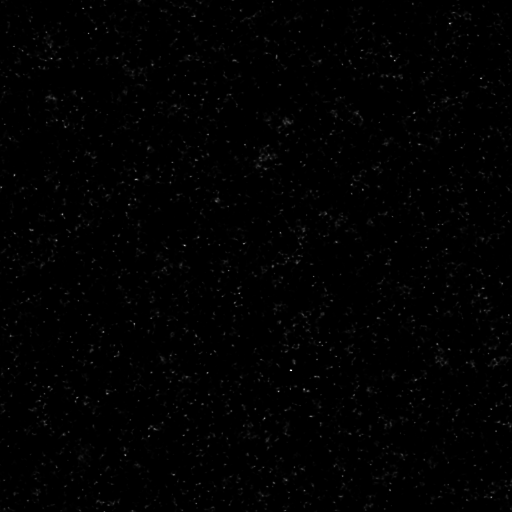

Supplement: Supplementary file 15 — Source data Fig. 6 [file 44319_2025_644_MOESM15_ESM.zip › Figure 6/6I/Ctl/Slice2_2-6/AVG_C2-ctl1_exp14aug_homer- slice2_2-6-8tif512-1_deconv.tif]

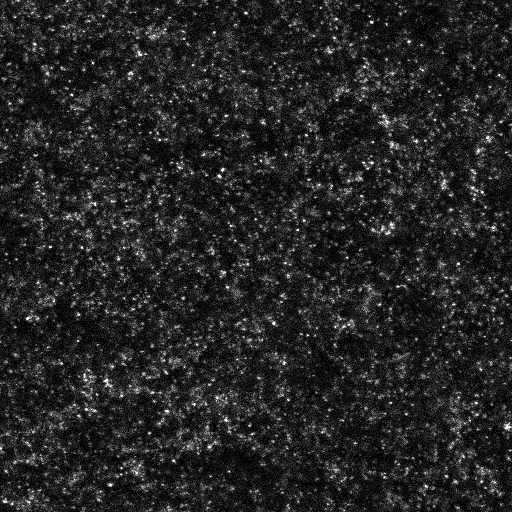

Supplement: Supplementary file 15 — Source data Fig. 6 [file 44319_2025_644_MOESM15_ESM.zip › Figure 6/6I/Ctl/Slice2_2-6/AVG_C2-ctl1_exp14aug_homer- slice2_2-6-8tif512-2.tif]

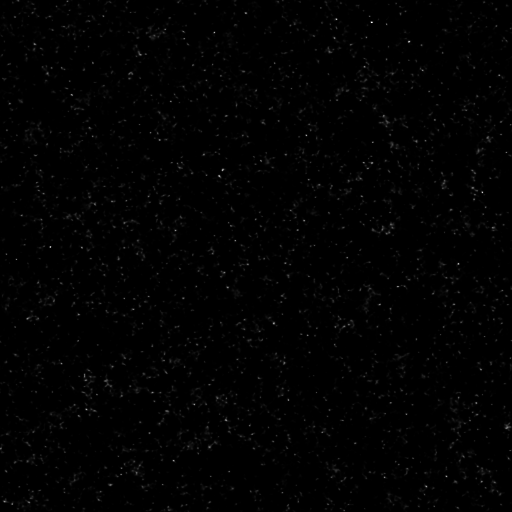

Supplement: Supplementary file 15 — Source data Fig. 6 [file 44319_2025_644_MOESM15_ESM.zip › Figure 6/6I/Ctl/Slice2_2-6/AVG_C2-ctl1_exp14aug_homer- slice2_2-6-8tif512-2_deconv.tif]

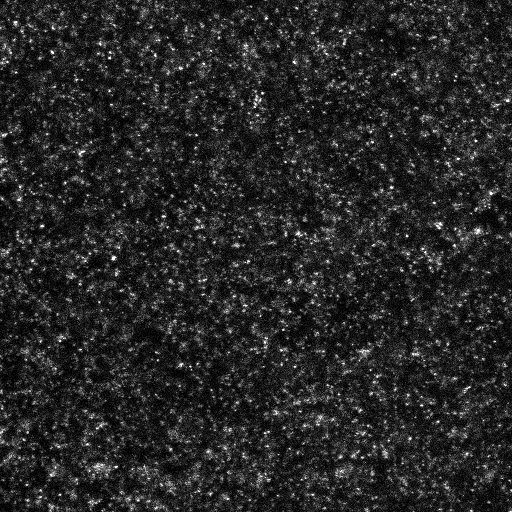

Supplement: Supplementary file 15 — Source data Fig. 6 [file 44319_2025_644_MOESM15_ESM.zip › Figure 6/6I/Ctl/Slice2_2-6/AVG_C2-ctl1_exp14aug_homer- slice2_2-6-8tif512-3.tif]

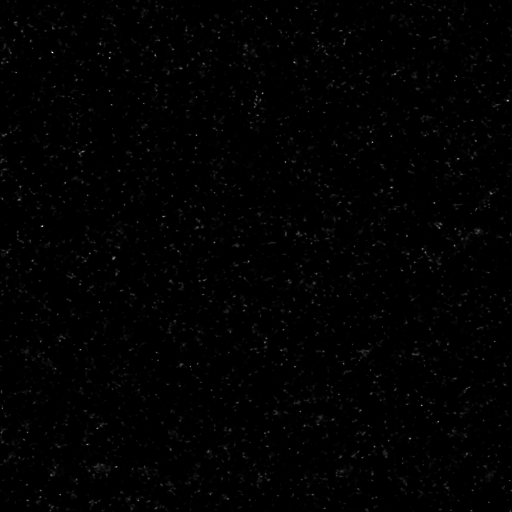

Supplement: Supplementary file 15 — Source data Fig. 6 [file 44319_2025_644_MOESM15_ESM.zip › Figure 6/6I/Ctl/Slice2_2-6/AVG_C2-ctl1_exp14aug_homer- slice2_2-6-8tif512-3_deconv.tif]

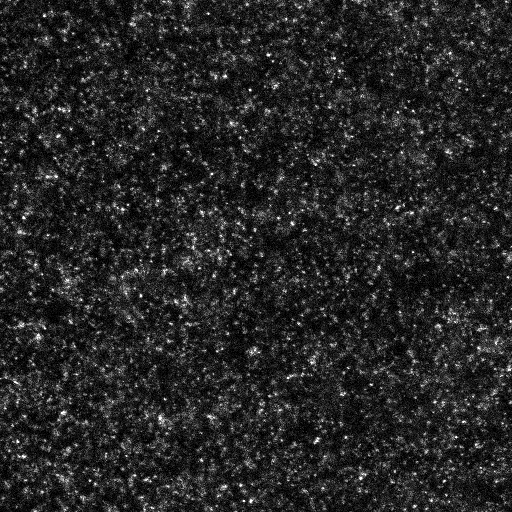

Supplement: Supplementary file 15 — Source data Fig. 6 [file 44319_2025_644_MOESM15_ESM.zip › Figure 6/6I/Ctl/Slice2_2-6/AVG_C2-ctl1_exp14aug_homer- slice2_2-6-8tif512-4.tif]

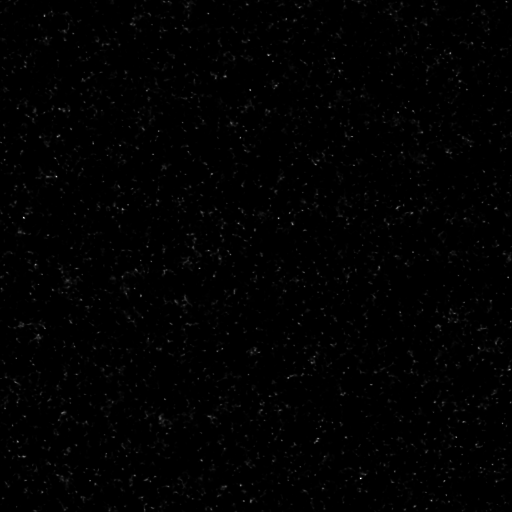

Supplement: Supplementary file 15 — Source data Fig. 6 [file 44319_2025_644_MOESM15_ESM.zip › Figure 6/6I/Ctl/Slice2_2-6/AVG_C2-ctl1_exp14aug_homer- slice2_2-6-8tif512-4_deconv.tif]

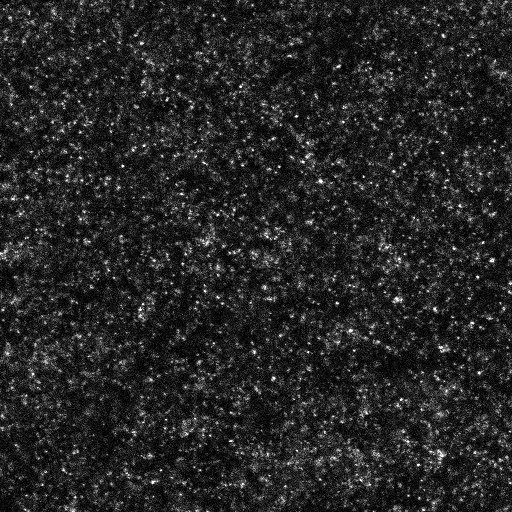

Supplement: Supplementary file 15 — Source data Fig. 6 [file 44319_2025_644_MOESM15_ESM.zip › Figure 6/6I/Ctl/Slice2_2_1-3/AVG_C2-ctl1_exp14aug_homer- slice2_2-1-3.tif512-1.tif]

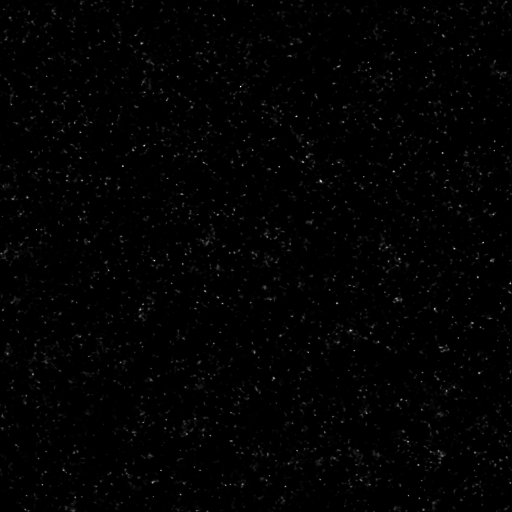

Supplement: Supplementary file 15 — Source data Fig. 6 [file 44319_2025_644_MOESM15_ESM.zip › Figure 6/6I/Ctl/Slice2_2_1-3/AVG_C2-ctl1_exp14aug_homer- slice2_2-1-3.tif512-1_deconv.tif]

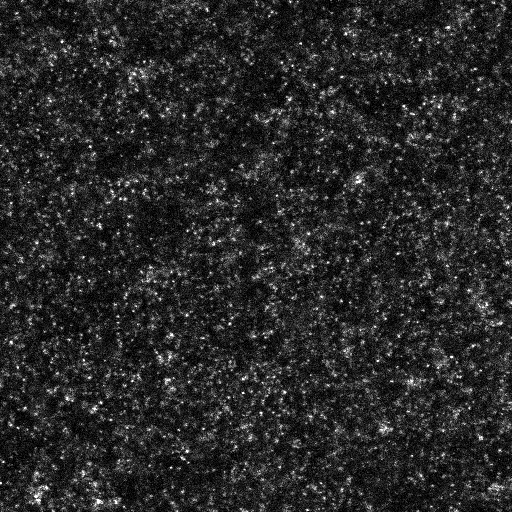

Supplement: Supplementary file 15 — Source data Fig. 6 [file 44319_2025_644_MOESM15_ESM.zip › Figure 6/6I/Ctl/Slice2_2_1-3/AVG_C2-ctl1_exp14aug_homer- slice2_2-1-3.tif512-2.tif]

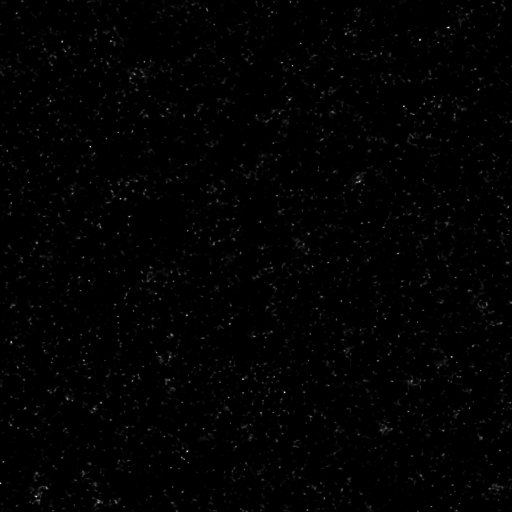

Supplement: Supplementary file 15 — Source data Fig. 6 [file 44319_2025_644_MOESM15_ESM.zip › Figure 6/6I/Ctl/Slice2_2_1-3/AVG_C2-ctl1_exp14aug_homer- slice2_2-1-3.tif512-2_deconv.tif]

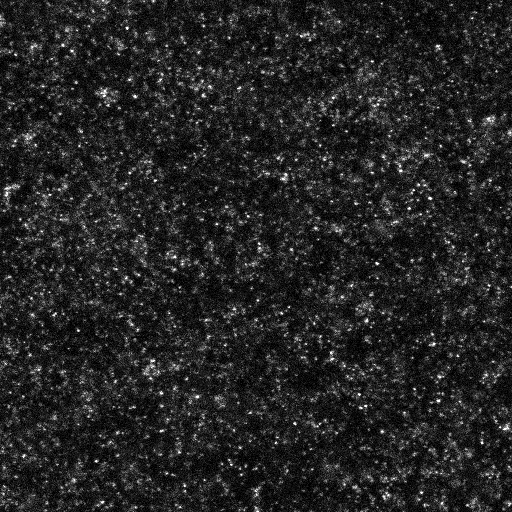

Supplement: Supplementary file 15 — Source data Fig. 6 [file 44319_2025_644_MOESM15_ESM.zip › Figure 6/6I/Ctl/Slice2_2_1-3/AVG_C2-ctl1_exp14aug_homer- slice2_2-1-3.tif512-3.tif]

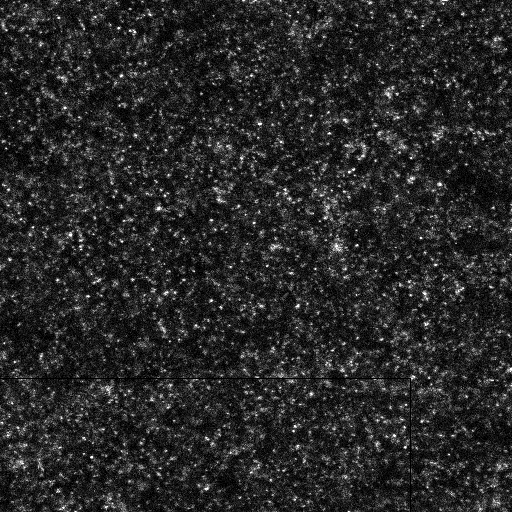

Supplement: Supplementary file 15 — Source data Fig. 6 [file 44319_2025_644_MOESM15_ESM.zip › Figure 6/6I/Ctl/Slice2_2_1-3/AVG_C2-ctl1_exp14aug_homer- slice2_2-1-3.tif512-4.tif]

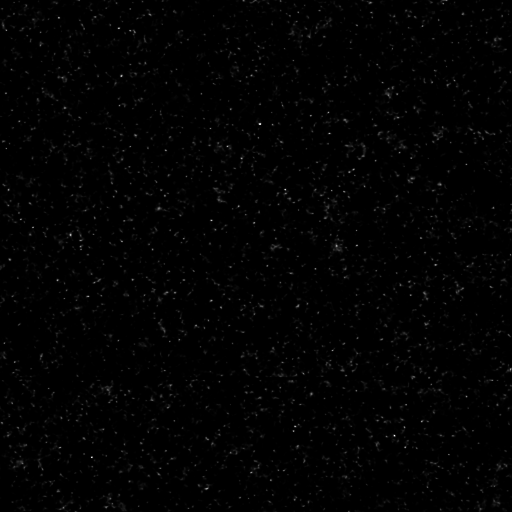

Supplement: Supplementary file 15 — Source data Fig. 6 [file 44319_2025_644_MOESM15_ESM.zip › Figure 6/6I/Ctl/Slice2_2_1-3/AVG_C2-ctl1_exp14aug_homer- slice2_2-1-3.tif512-4_deconv.tif]

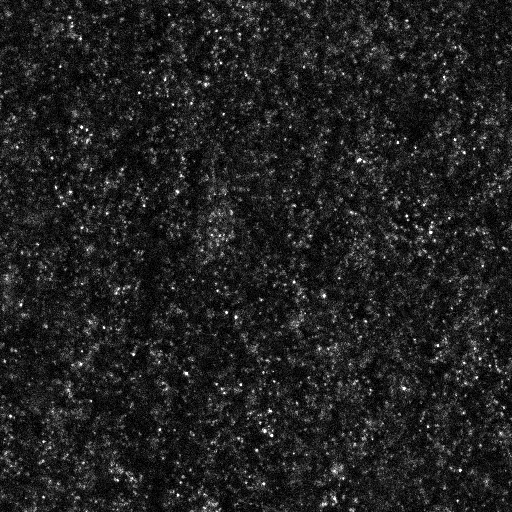

Supplement: Supplementary file 15 — Source data Fig. 6 [file 44319_2025_644_MOESM15_ESM.zip › Figure 6/6I/PFR/slice1_1-1-3/AVG_C2-pfr_exp14aug_homer- slice1_1-1-3 512-1.tif]

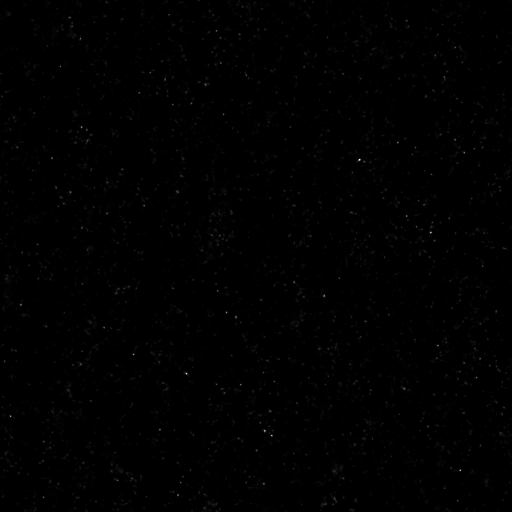

Supplement: Supplementary file 15 — Source data Fig. 6 [file 44319_2025_644_MOESM15_ESM.zip › Figure 6/6I/PFR/slice1_1-1-3/AVG_C2-pfr_exp14aug_homer- slice1_1-1-3 512-1_deconv.tif]

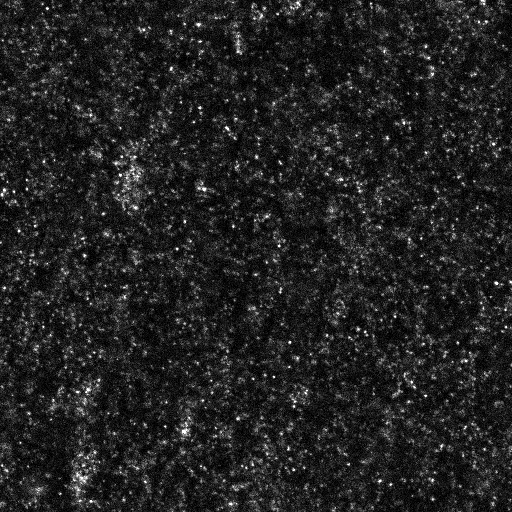

Supplement: Supplementary file 15 — Source data Fig. 6 [file 44319_2025_644_MOESM15_ESM.zip › Figure 6/6I/PFR/slice1_1-1-3/AVG_C2-pfr_exp14aug_homer- slice1_1-1-3 512-2.tif]

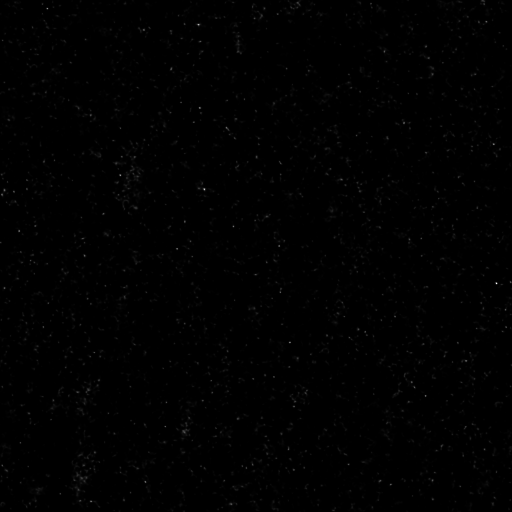

Supplement: Supplementary file 15 — Source data Fig. 6 [file 44319_2025_644_MOESM15_ESM.zip › Figure 6/6I/PFR/slice1_1-1-3/AVG_C2-pfr_exp14aug_homer- slice1_1-1-3 512-2_deconv.tif]

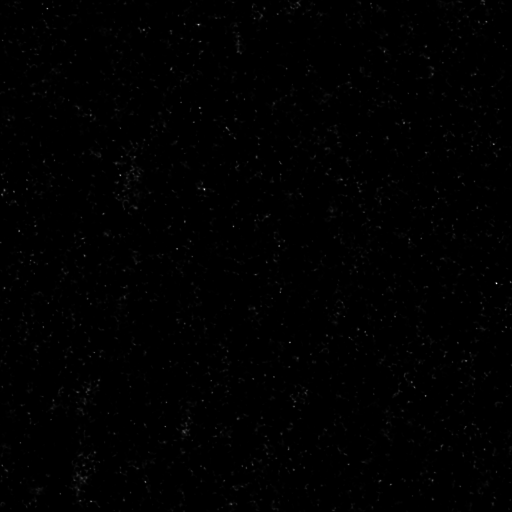

Supplement: Supplementary file 15 — Source data Fig. 6 [file 44319_2025_644_MOESM15_ESM.zip › Figure 6/6I/PFR/slice1_1-1-3/AVG_C2-pfr_exp14aug_homer- slice1_1-1-3 512-3_deconv.tif]

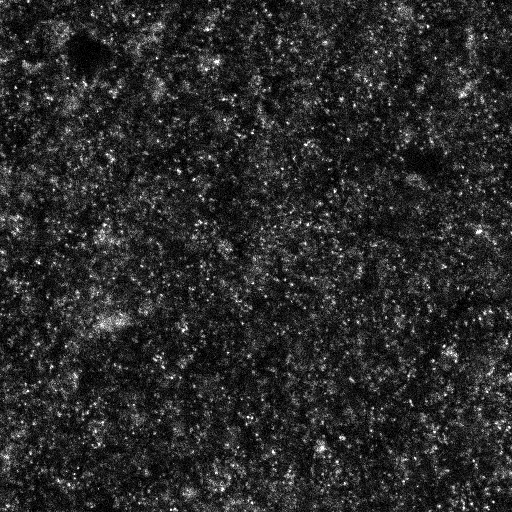

Supplement: Supplementary file 15 — Source data Fig. 6 [file 44319_2025_644_MOESM15_ESM.zip › Figure 6/6I/PFR/slice1_1-1-3/AVG_C2-pfr_exp14aug_homer- slice1_1-1-3 512-4.tif]

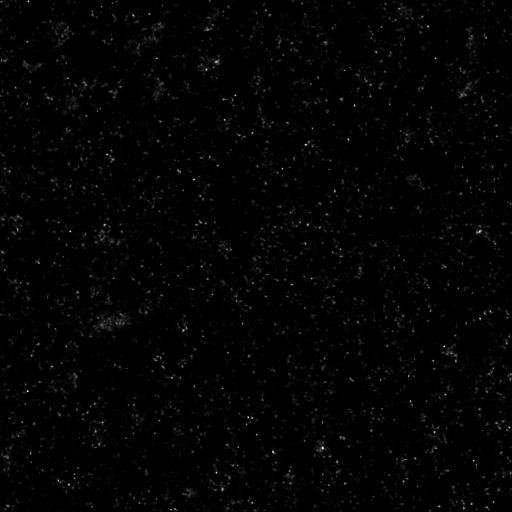

Supplement: Supplementary file 15 — Source data Fig. 6 [file 44319_2025_644_MOESM15_ESM.zip › Figure 6/6I/PFR/slice1_1-1-3/AVG_C2-pfr_exp14aug_homer- slice1_1-1-3 512-4_deconv.tif]

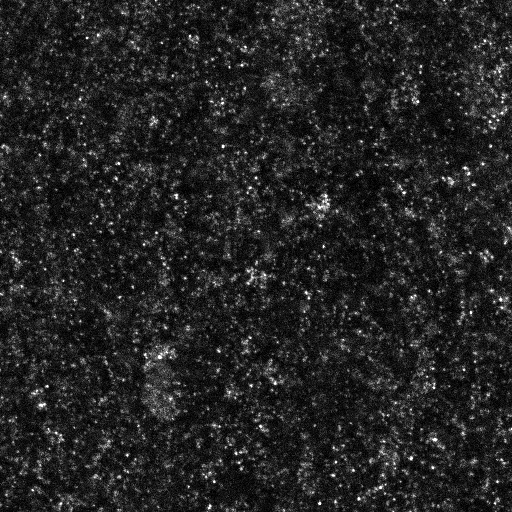

Supplement: Supplementary file 15 — Source data Fig. 6 [file 44319_2025_644_MOESM15_ESM.zip › Figure 6/6I/PFR/slice1_1-6-9/AVG_C2-pfr_exp14aug_homer- slice1_1-6-9 512-1.tif]

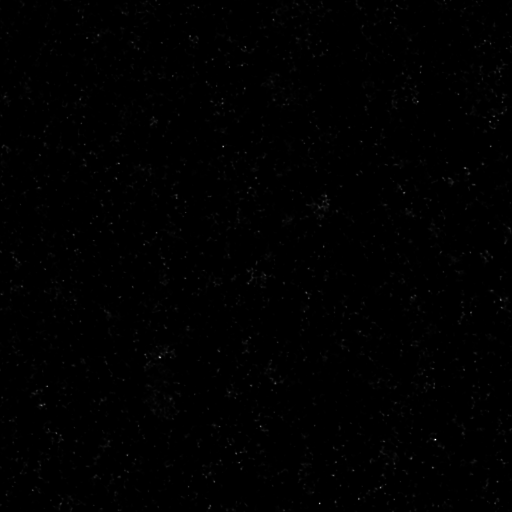

Supplement: Supplementary file 15 — Source data Fig. 6 [file 44319_2025_644_MOESM15_ESM.zip › Figure 6/6I/PFR/slice1_1-6-9/AVG_C2-pfr_exp14aug_homer- slice1_1-6-9 512-1_deconv.tif]

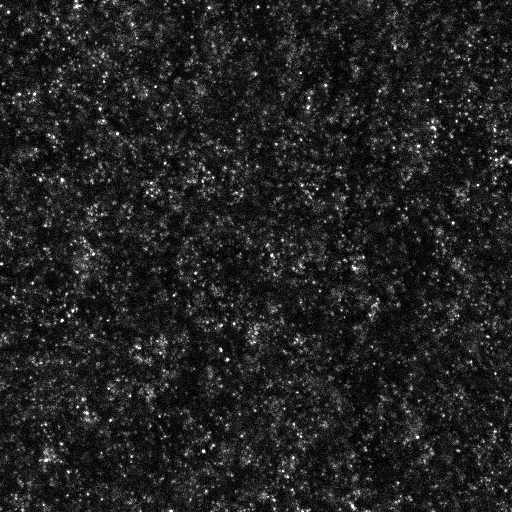

Supplement: Supplementary file 15 — Source data Fig. 6 [file 44319_2025_644_MOESM15_ESM.zip › Figure 6/6I/PFR/slice1_1-6-9/AVG_C2-pfr_exp14aug_homer- slice1_1-6-9 512-2.tif]

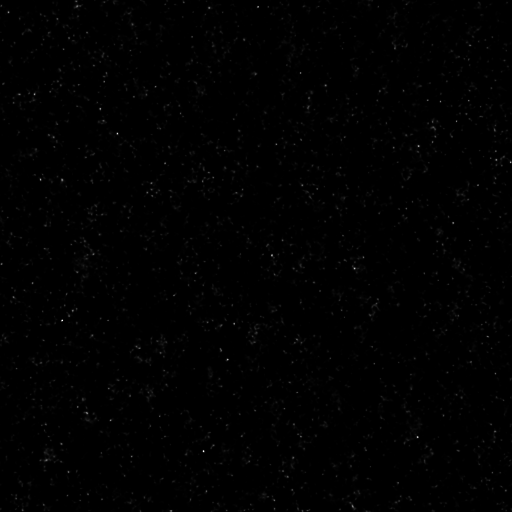

Supplement: Supplementary file 15 — Source data Fig. 6 [file 44319_2025_644_MOESM15_ESM.zip › Figure 6/6I/PFR/slice1_1-6-9/AVG_C2-pfr_exp14aug_homer- slice1_1-6-9 512-2_deconv.tif]

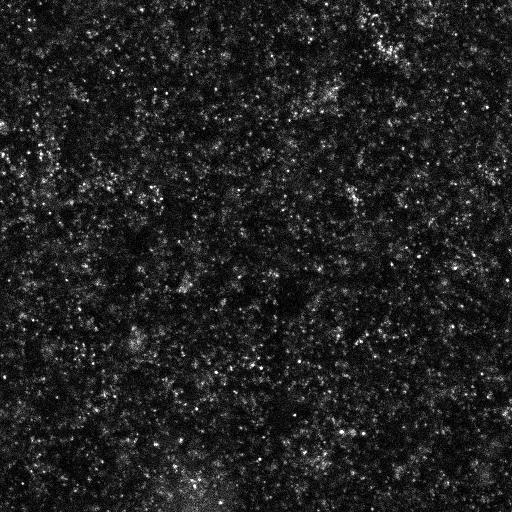

Supplement: Supplementary file 15 — Source data Fig. 6 [file 44319_2025_644_MOESM15_ESM.zip › Figure 6/6I/PFR/slice1_1-6-9/AVG_C2-pfr_exp14aug_homer- slice1_1-6-9 512-3.tif]

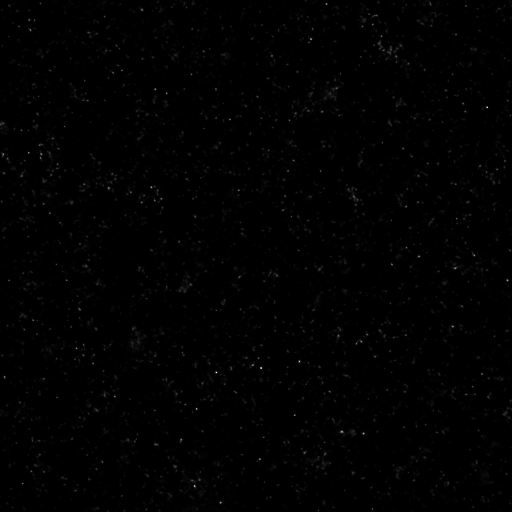

Supplement: Supplementary file 15 — Source data Fig. 6 [file 44319_2025_644_MOESM15_ESM.zip › Figure 6/6I/PFR/slice1_1-6-9/AVG_C2-pfr_exp14aug_homer- slice1_1-6-9 512-3_deconv.tif]

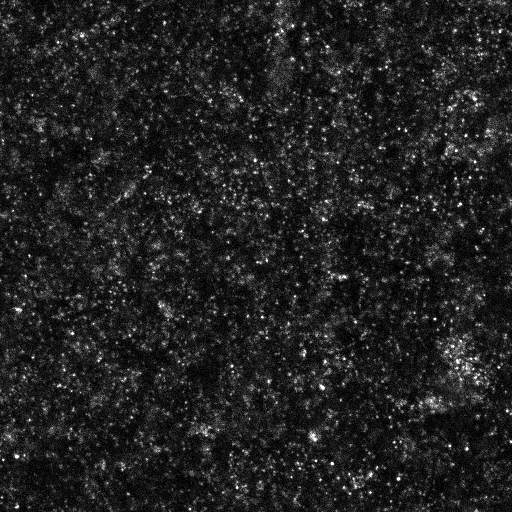

Supplement: Supplementary file 15 — Source data Fig. 6 [file 44319_2025_644_MOESM15_ESM.zip › Figure 6/6I/PFR/slice1_1-6-9/AVG_C2-pfr_exp14aug_homer- slice1_1-6-9 512-4.tif]

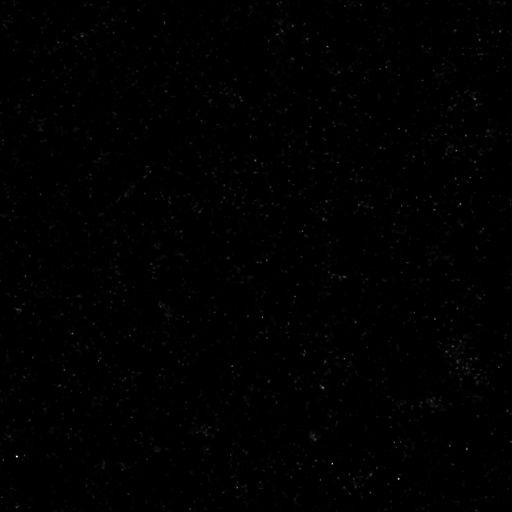

Supplement: Supplementary file 15 — Source data Fig. 6 [file 44319_2025_644_MOESM15_ESM.zip › Figure 6/6I/PFR/slice1_1-6-9/AVG_C2-pfr_exp14aug_homer- slice1_1-6-9 512-4_deconv.tif]

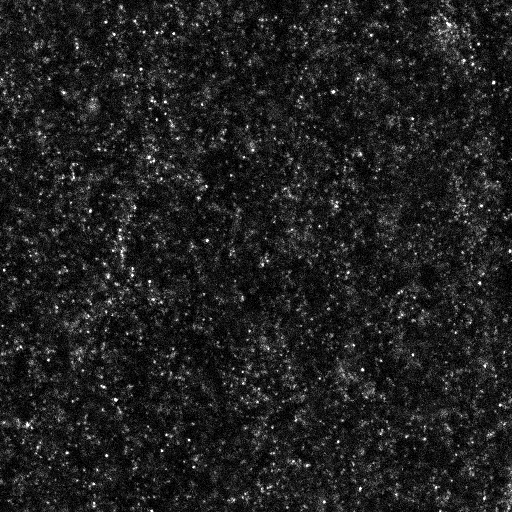

Supplement: Supplementary file 15 — Source data Fig. 6 [file 44319_2025_644_MOESM15_ESM.zip › Figure 6/6I/PFR/slice1_2-1-3/AVG_C2-pfr_exp14aug_homer- slice1_2-1-3 512-1.tif]

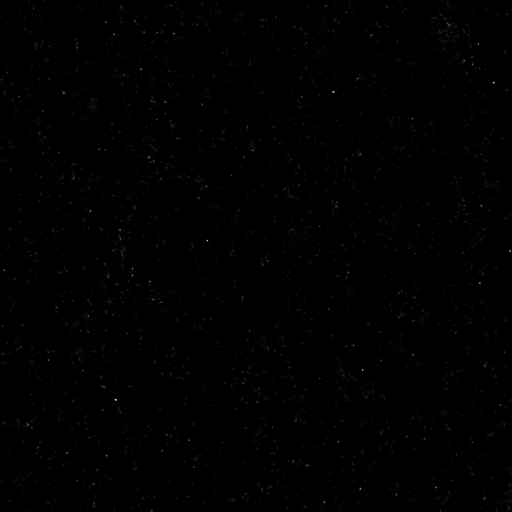

Supplement: Supplementary file 15 — Source data Fig. 6 [file 44319_2025_644_MOESM15_ESM.zip › Figure 6/6I/PFR/slice1_2-1-3/AVG_C2-pfr_exp14aug_homer- slice1_2-1-3 512-1_deconv.tif]

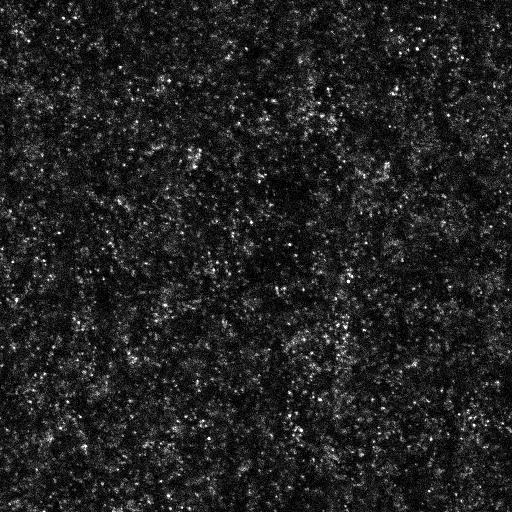

Supplement: Supplementary file 15 — Source data Fig. 6 [file 44319_2025_644_MOESM15_ESM.zip › Figure 6/6I/PFR/slice1_2-1-3/AVG_C2-pfr_exp14aug_homer- slice1_2-1-3 512-2.tif]

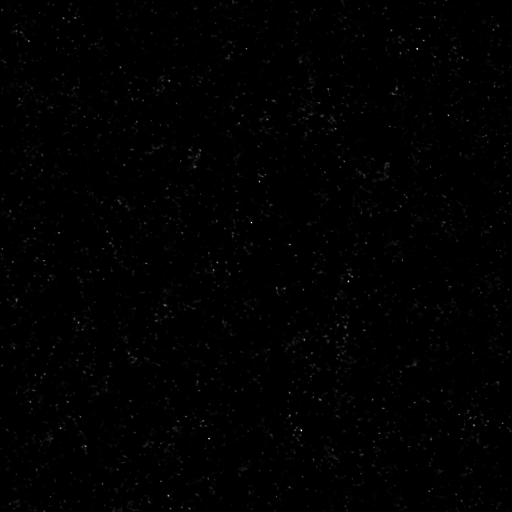

Supplement: Supplementary file 15 — Source data Fig. 6 [file 44319_2025_644_MOESM15_ESM.zip › Figure 6/6I/PFR/slice1_2-1-3/AVG_C2-pfr_exp14aug_homer- slice1_2-1-3 512-2_deconv.tif]

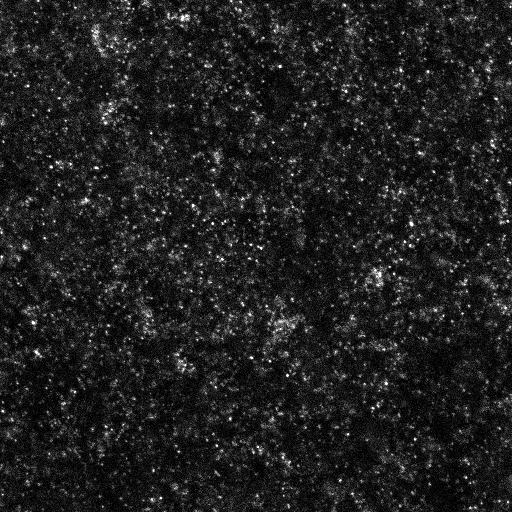

Supplement: Supplementary file 15 — Source data Fig. 6 [file 44319_2025_644_MOESM15_ESM.zip › Figure 6/6I/PFR/slice1_2-1-3/AVG_C2-pfr_exp14aug_homer- slice1_2-1-3 512-3.tif]

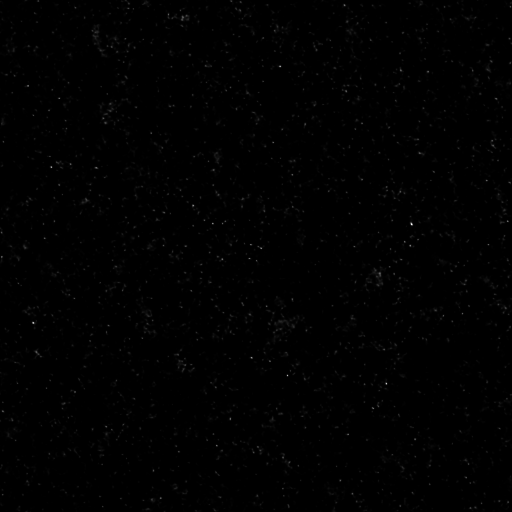

Supplement: Supplementary file 15 — Source data Fig. 6 [file 44319_2025_644_MOESM15_ESM.zip › Figure 6/6I/PFR/slice1_2-1-3/AVG_C2-pfr_exp14aug_homer- slice1_2-1-3 512-3_deconv.tif]

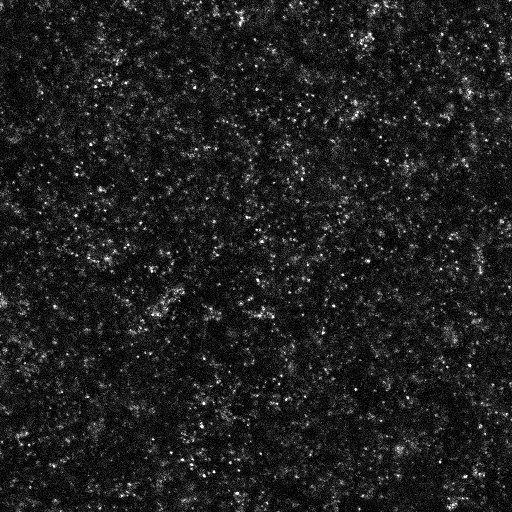

Supplement: Supplementary file 15 — Source data Fig. 6 [file 44319_2025_644_MOESM15_ESM.zip › Figure 6/6I/PFR/slice1_2-1-3/AVG_C2-pfr_exp14aug_homer- slice1_2-1-3 512-4.tif]

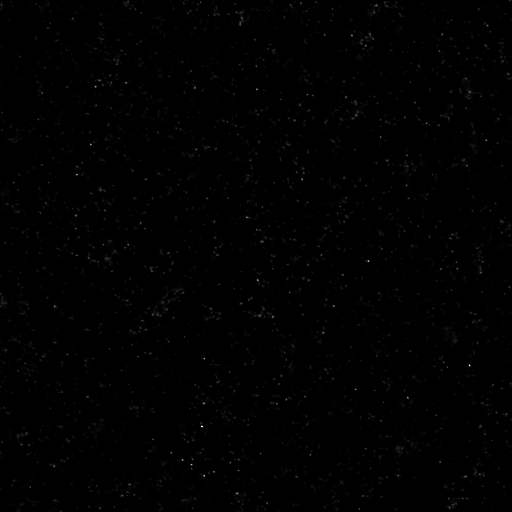

Supplement: Supplementary file 15 — Source data Fig. 6 [file 44319_2025_644_MOESM15_ESM.zip › Figure 6/6I/PFR/slice1_2-1-3/AVG_C2-pfr_exp14aug_homer- slice1_2-1-3 512-4_deconv.tif]

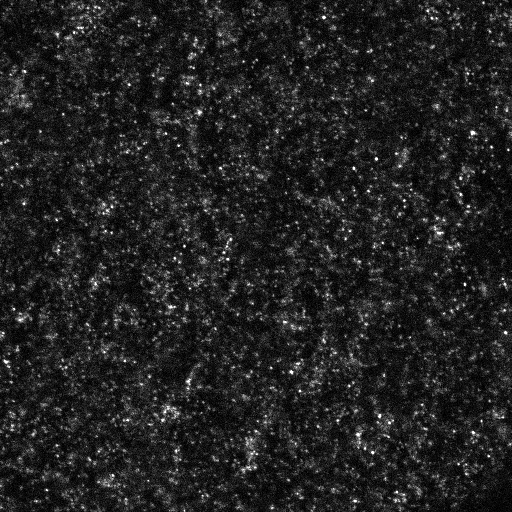

Supplement: Supplementary file 15 — Source data Fig. 6 [file 44319_2025_644_MOESM15_ESM.zip › Figure 6/6I/PFR/slice1_2-7-10/AVG_C2-pfr_exp14aug_homer- slice1_2-7-10 512-1.tif]

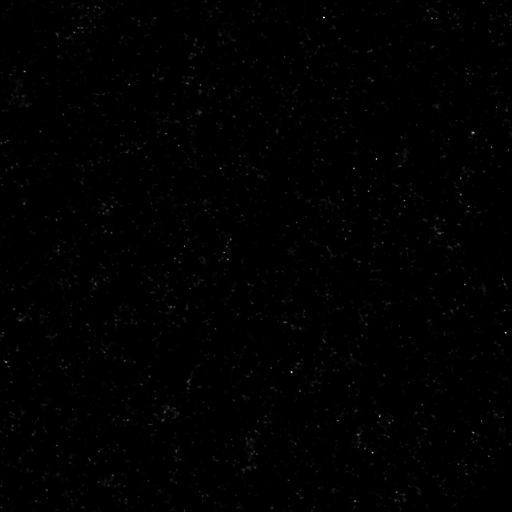

Supplement: Supplementary file 15 — Source data Fig. 6 [file 44319_2025_644_MOESM15_ESM.zip › Figure 6/6I/PFR/slice1_2-7-10/AVG_C2-pfr_exp14aug_homer- slice1_2-7-10 512-1_deconv.tif]

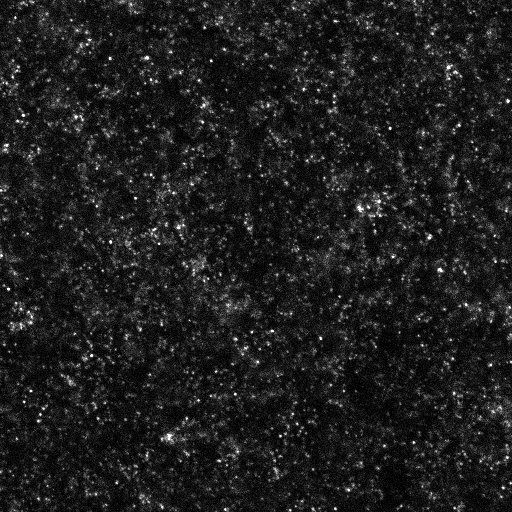

Supplement: Supplementary file 15 — Source data Fig. 6 [file 44319_2025_644_MOESM15_ESM.zip › Figure 6/6I/PFR/slice1_2-7-10/AVG_C2-pfr_exp14aug_homer- slice1_2-7-10 512-2.tif]

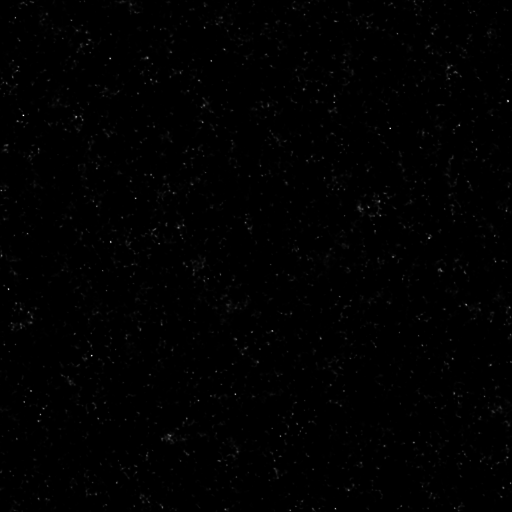

Supplement: Supplementary file 15 — Source data Fig. 6 [file 44319_2025_644_MOESM15_ESM.zip › Figure 6/6I/PFR/slice1_2-7-10/AVG_C2-pfr_exp14aug_homer- slice1_2-7-10 512-2_deconv.tif]

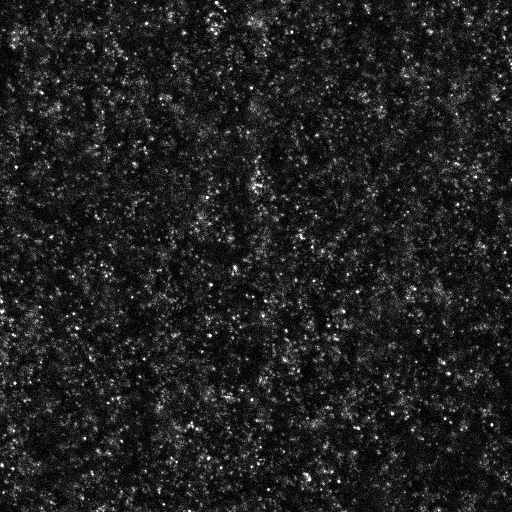

Supplement: Supplementary file 15 — Source data Fig. 6 [file 44319_2025_644_MOESM15_ESM.zip › Figure 6/6I/PFR/slice1_2-7-10/AVG_C2-pfr_exp14aug_homer- slice1_2-7-10 512-3.tif]

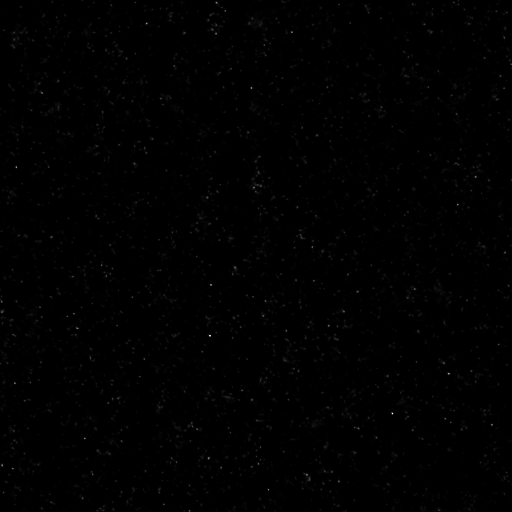

Supplement: Supplementary file 15 — Source data Fig. 6 [file 44319_2025_644_MOESM15_ESM.zip › Figure 6/6I/PFR/slice1_2-7-10/AVG_C2-pfr_exp14aug_homer- slice1_2-7-10 512-3_deconv.tif]

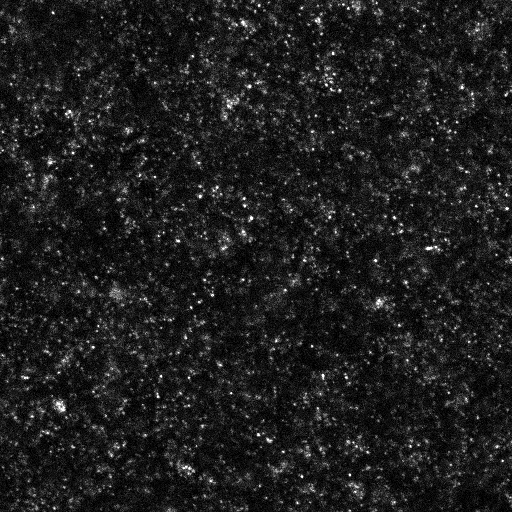

Supplement: Supplementary file 15 — Source data Fig. 6 [file 44319_2025_644_MOESM15_ESM.zip › Figure 6/6I/PFR/slice1_2-7-10/AVG_C2-pfr_exp14aug_homer- slice1_2-7-10 512-4.tif]

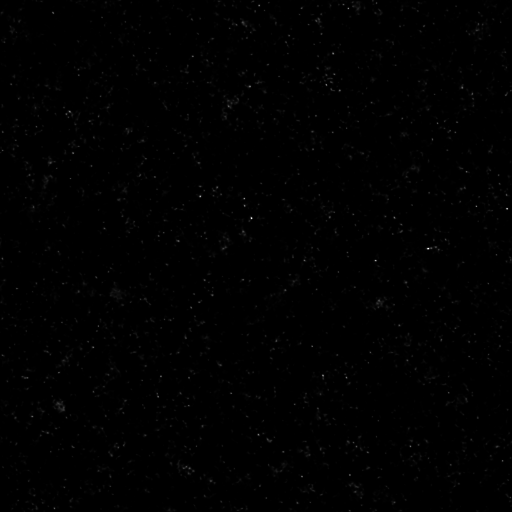

Supplement: Supplementary file 15 — Source data Fig. 6 [file 44319_2025_644_MOESM15_ESM.zip › Figure 6/6I/PFR/slice1_2-7-10/AVG_C2-pfr_exp14aug_homer- slice1_2-7-10 512-4_deconv.tif]

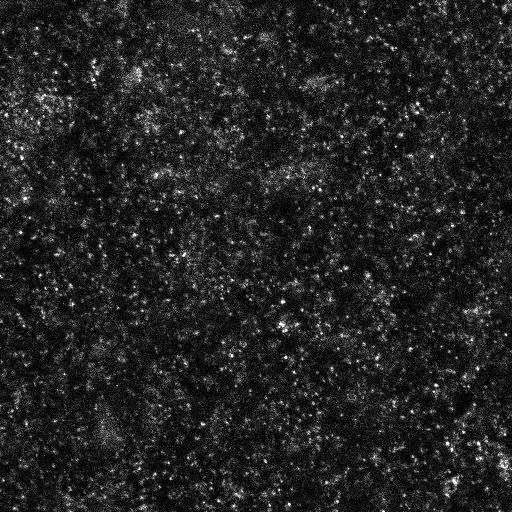

Supplement: Supplementary file 15 — Source data Fig. 6 [file 44319_2025_644_MOESM15_ESM.zip › Figure 6/6I/PFR/slice2_1-1-3/AVG_C2-pfr_exp14aug_homer- slice2_1-1-3 512-1.tif]

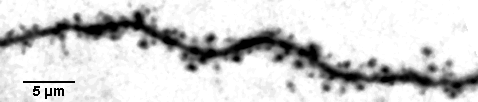

Supplement: Supplementary file 16 — Source data Fig. 7 [file 44319_2025_644_MOESM16_ESM.zip › Figure 7/7D/Ctl/Ctl final.tif]

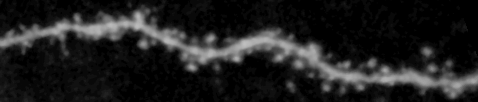

Supplement: Supplementary file 16 — Source data Fig. 7 [file 44319_2025_644_MOESM16_ESM.zip › Figure 7/7D/Ctl/Ctl-058.tif]

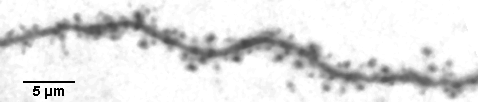

Supplement: Supplementary file 16 — Source data Fig. 7 [file 44319_2025_644_MOESM16_ESM.zip › Figure 7/7D/Ctl/Ctl.tif]

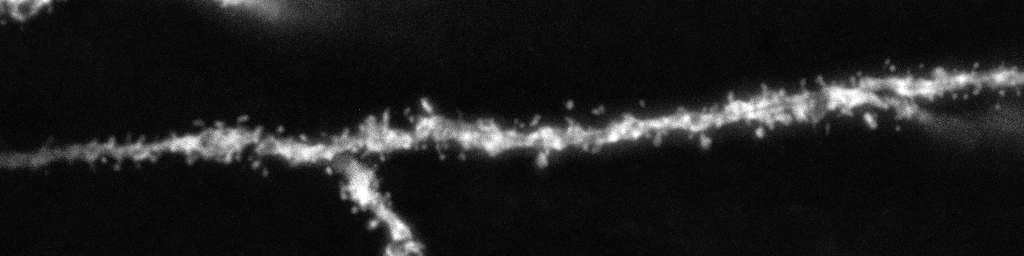

Supplement: Supplementary file 16 — Source data Fig. 7 [file 44319_2025_644_MOESM16_ESM.zip › Figure 7/7D/Ctl/exp080316/MAX_ctl - Series008.tif]

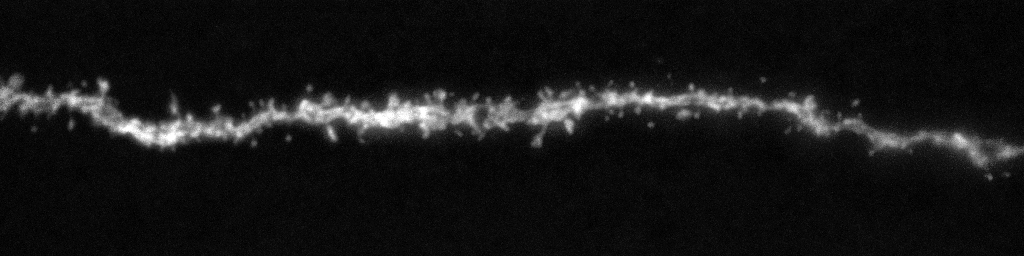

Supplement: Supplementary file 16 — Source data Fig. 7 [file 44319_2025_644_MOESM16_ESM.zip › Figure 7/7D/Ctl/exp080316/MAX_ctl- Series012.tif]

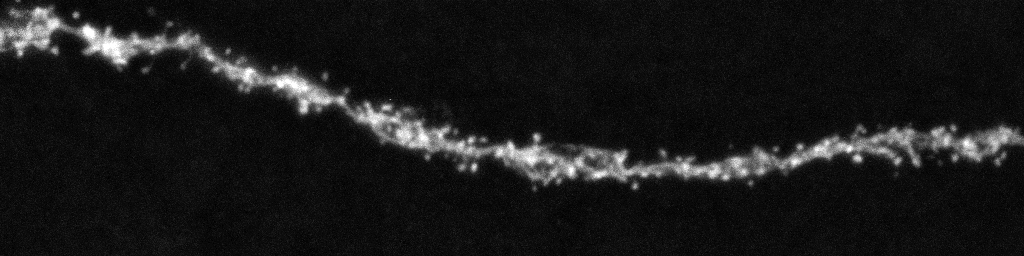

Supplement: Supplementary file 16 — Source data Fig. 7 [file 44319_2025_644_MOESM16_ESM.zip › Figure 7/7D/Ctl/exp080316/MAX_ctl- Series015.tif]

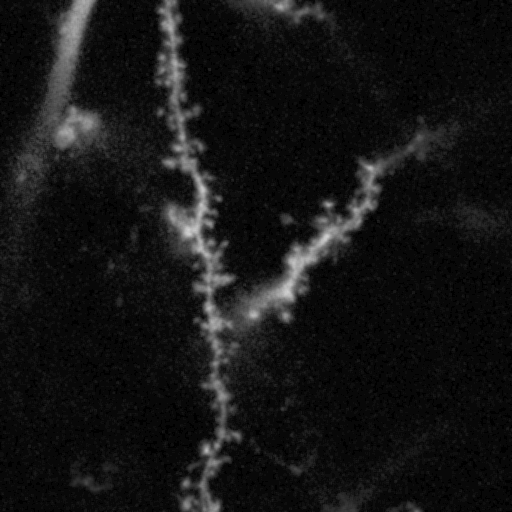

Supplement: Supplementary file 16 — Source data Fig. 7 [file 44319_2025_644_MOESM16_ESM.zip › Figure 7/7D/Ctl/exp230515/MAX_ctl_23_5_13-Series013.tif]

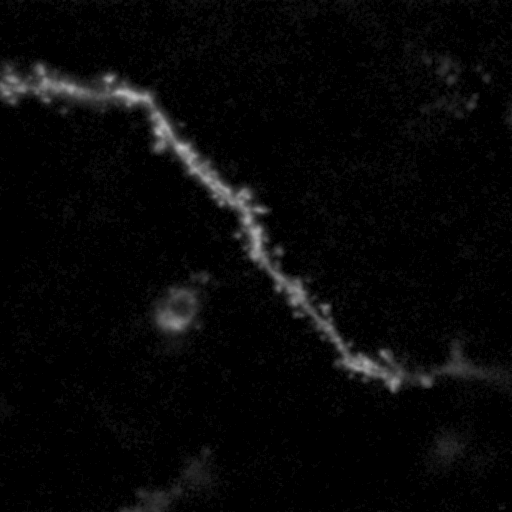

Supplement: Supplementary file 16 — Source data Fig. 7 [file 44319_2025_644_MOESM16_ESM.zip › Figure 7/7D/Ctl/exp230515/MAX_ctl_23_5_13_exp- Series023.tif]

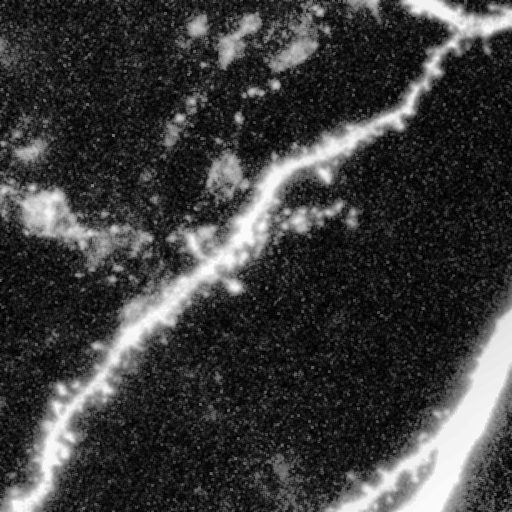

Supplement: Supplementary file 16 — Source data Fig. 7 [file 44319_2025_644_MOESM16_ESM.zip › Figure 7/7D/Ctl/exp230515/MAX_ctl_23_5_13_exp-Series018.tif]

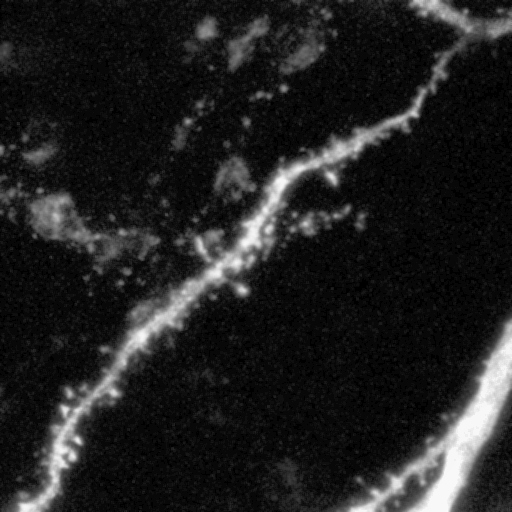

Supplement: Supplementary file 16 — Source data Fig. 7 [file 44319_2025_644_MOESM16_ESM.zip › Figure 7/7D/Ctl/exp230515/MAX_ctl_23_5_13_exp-Series020.tif]

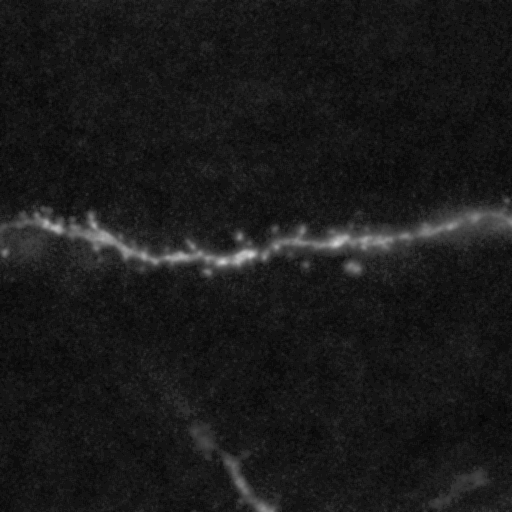

Supplement: Supplementary file 16 — Source data Fig. 7 [file 44319_2025_644_MOESM16_ESM.zip › Figure 7/7D/Ctl/exp230515/MAX_ctl_23_5_13_exp-Series030.tif]

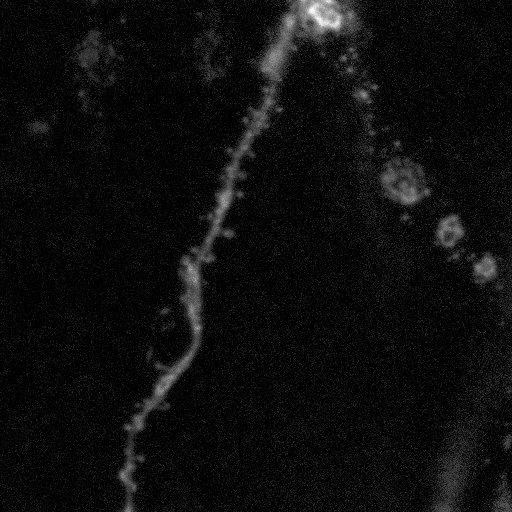

Supplement: Supplementary file 16 — Source data Fig. 7 [file 44319_2025_644_MOESM16_ESM.zip › Figure 7/7D/Ctl/exp230515/MAX_ctl_23_5_13_exp-Series035.tif]

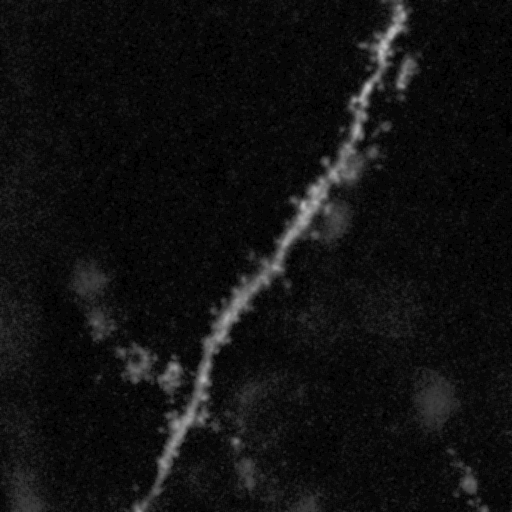

Supplement: Supplementary file 16 — Source data Fig. 7 [file 44319_2025_644_MOESM16_ESM.zip › Figure 7/7D/Ctl/exp230515/MAX_ctl_23_5_13_exp-Series039.tif]

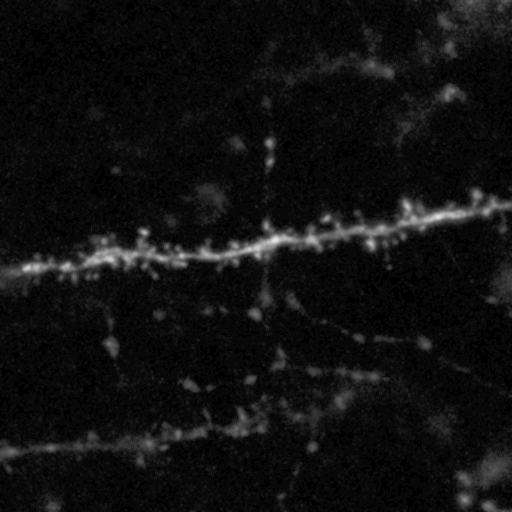

Supplement: Supplementary file 16 — Source data Fig. 7 [file 44319_2025_644_MOESM16_ESM.zip › Figure 7/7D/Ctl/exp230515/MAX_ctl_23_5_13_exp-Series050-1.tif]

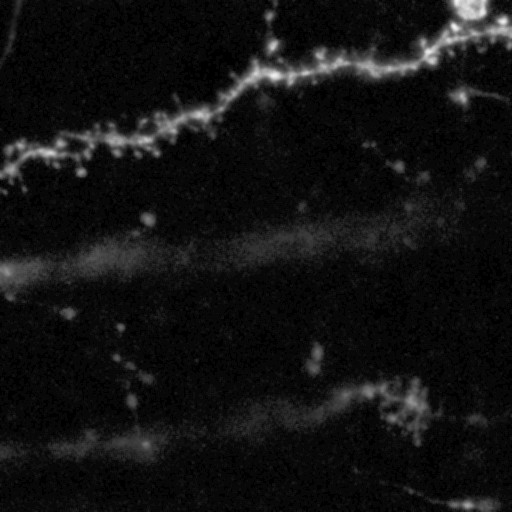

Supplement: Supplementary file 16 — Source data Fig. 7 [file 44319_2025_644_MOESM16_ESM.zip › Figure 7/7D/Ctl/exp230515/MAX_ctl_23_5_13_exp-Series050-2.tif]

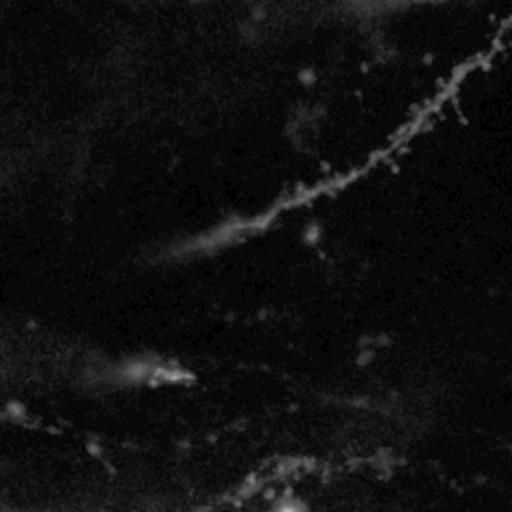

Supplement: Supplementary file 16 — Source data Fig. 7 [file 44319_2025_644_MOESM16_ESM.zip › Figure 7/7D/Ctl/exp230515/MAX_ctl_23_5_13_exp-Series053.tif]

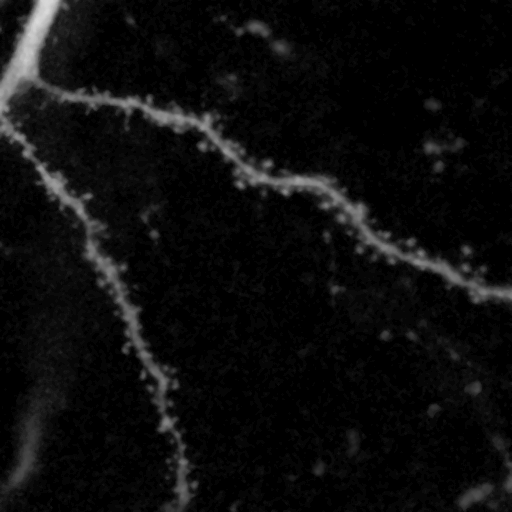

Supplement: Supplementary file 16 — Source data Fig. 7 [file 44319_2025_644_MOESM16_ESM.zip › Figure 7/7D/Ctl/exp230515/MAX_ctl_23_5_13_exp-Series058-BS-200_maxslope-4.tif]
