# Supplementary figures and images for: Activity-dependent extracellular proteolytic cascade cleaves the ECM component brevican to promote structural plasticity (part 3 of 3)
Source: EMBO Rep. 2025 Nov 19;27(1):163–85. doi: 10.1038/s44319-025-00644-w (PMC12796228; doi:10.1038/s44319-025-00644-w)

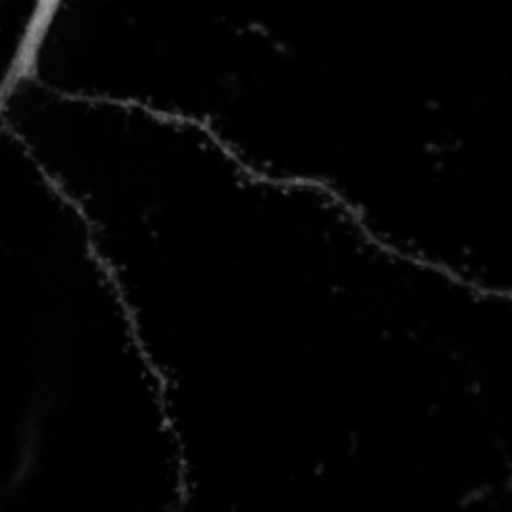

Supplement: Supplementary file 16 — Source data Fig. 7 [file 44319_2025_644_MOESM16_ESM.zip › Figure 7/7D/Ctl/exp230515/MAX_ctl_23_5_13_exp.Series058]

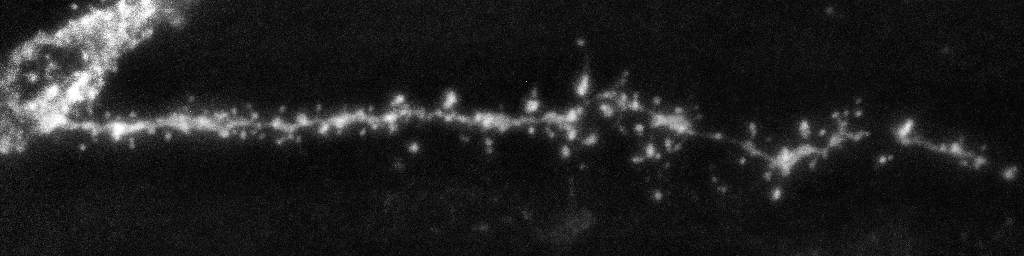

Supplement: Supplementary file 16 — Source data Fig. 7 [file 44319_2025_644_MOESM16_ESM.zip › Figure 7/7D/Ctl/exp30316/MAX_ctl - Series021.tif]

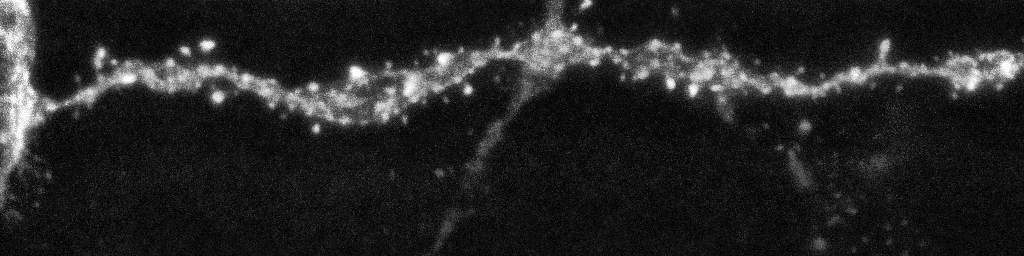

Supplement: Supplementary file 16 — Source data Fig. 7 [file 44319_2025_644_MOESM16_ESM.zip › Figure 7/7D/Ctl/exp30316/MAX_ctl - Series027.tif]

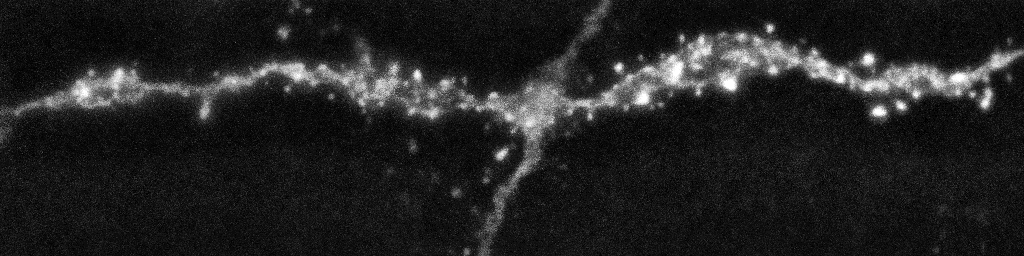

Supplement: Supplementary file 16 — Source data Fig. 7 [file 44319_2025_644_MOESM16_ESM.zip › Figure 7/7D/Ctl/exp30316/MAX_ctl- Series032.tif]

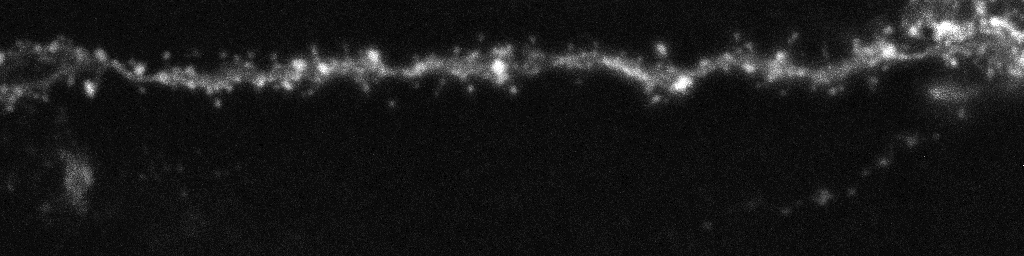

Supplement: Supplementary file 16 — Source data Fig. 7 [file 44319_2025_644_MOESM16_ESM.zip › Figure 7/7D/Ctl/exp30316/MAX_ctl- Series042.tif]

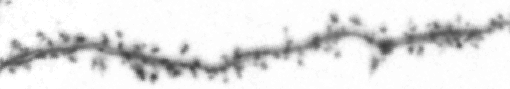

Supplement: Supplementary file 16 — Source data Fig. 7 [file 44319_2025_644_MOESM16_ESM.zip › Figure 7/7D/Ctl/PFR-Series002 cut.tif]

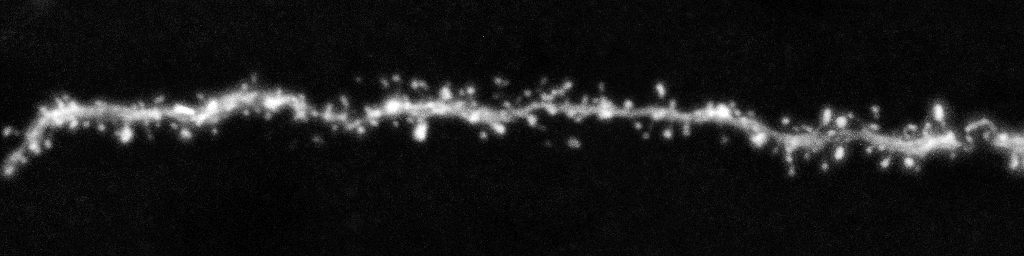

Supplement: Supplementary file 16 — Source data Fig. 7 [file 44319_2025_644_MOESM16_ESM.zip › Figure 7/7D/PFR/030316exp/MAX_PFR - Series018.tif]

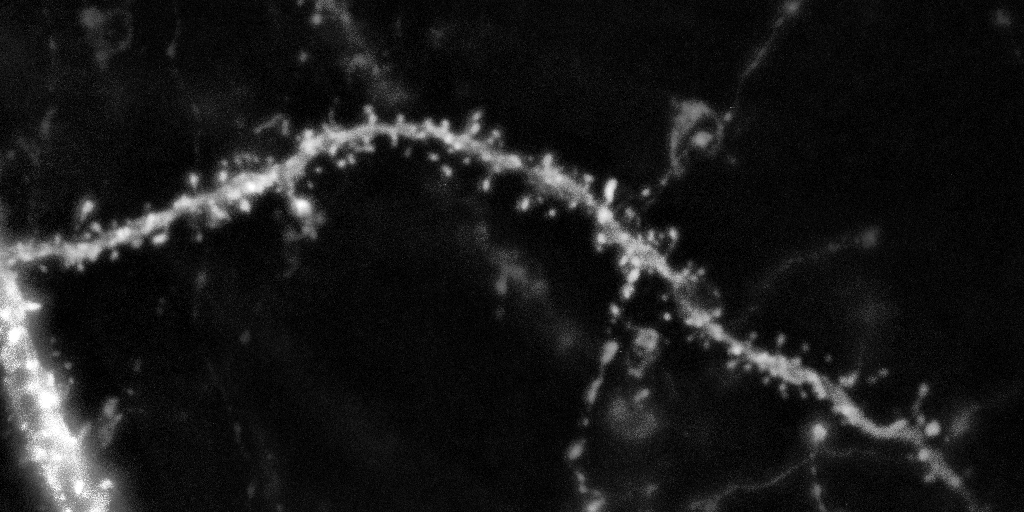

Supplement: Supplementary file 16 — Source data Fig. 7 [file 44319_2025_644_MOESM16_ESM.zip › Figure 7/7D/PFR/030316exp/MAX_PFR- Series007.tif]

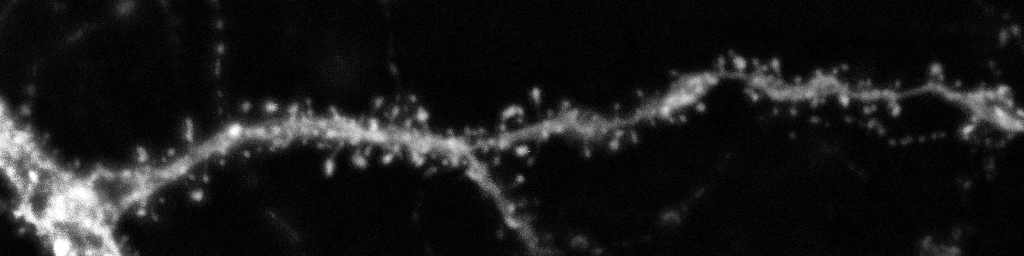

Supplement: Supplementary file 16 — Source data Fig. 7 [file 44319_2025_644_MOESM16_ESM.zip › Figure 7/7D/PFR/030316exp/MAX_PFR- Series010.tif]

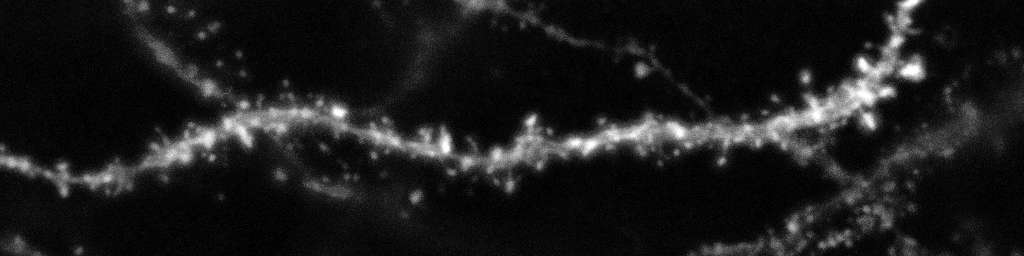

Supplement: Supplementary file 16 — Source data Fig. 7 [file 44319_2025_644_MOESM16_ESM.zip › Figure 7/7D/PFR/030316exp/MAX_PFR- Series021.tif]

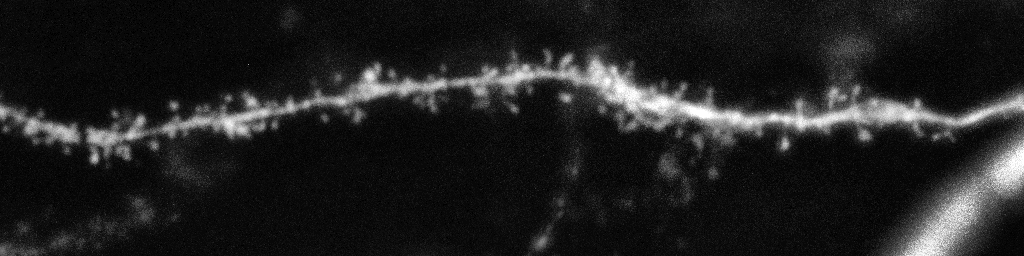

Supplement: Supplementary file 16 — Source data Fig. 7 [file 44319_2025_644_MOESM16_ESM.zip › Figure 7/7D/PFR/030316exp/MAX_PFR- Series047.tif]

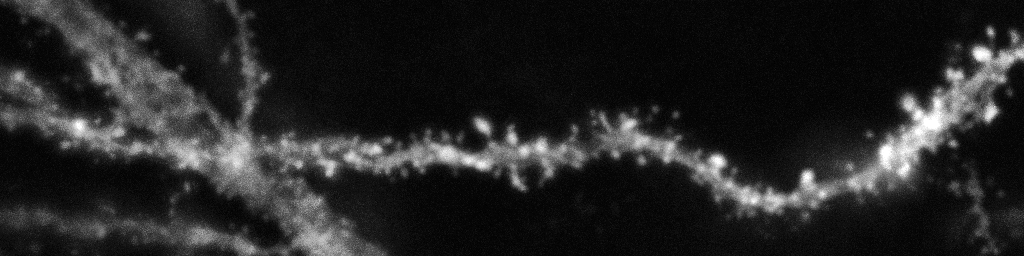

Supplement: Supplementary file 16 — Source data Fig. 7 [file 44319_2025_644_MOESM16_ESM.zip › Figure 7/7D/PFR/030316exp/MAX_PFR- Series052.tif]

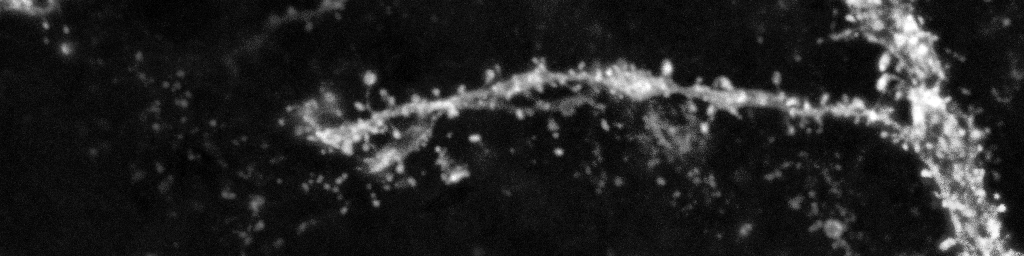

Supplement: Supplementary file 16 — Source data Fig. 7 [file 44319_2025_644_MOESM16_ESM.zip › Figure 7/7D/PFR/exp080316/MAX_PFR - Series018.tif]

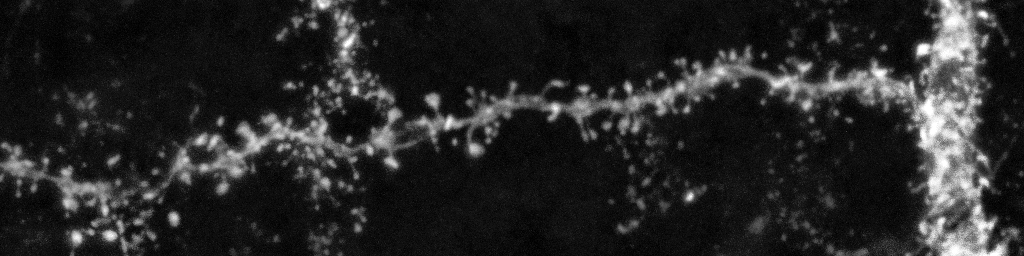

Supplement: Supplementary file 16 — Source data Fig. 7 [file 44319_2025_644_MOESM16_ESM.zip › Figure 7/7D/PFR/exp080316/MAX_PFR- Series003.tif]

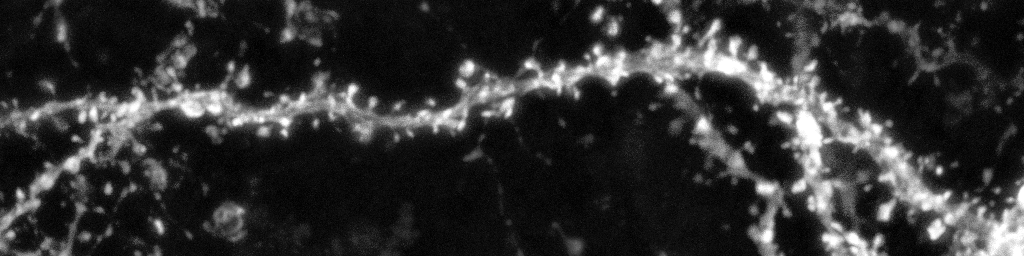

Supplement: Supplementary file 16 — Source data Fig. 7 [file 44319_2025_644_MOESM16_ESM.zip › Figure 7/7D/PFR/exp080316/MAX_PFR- Series007.tif]

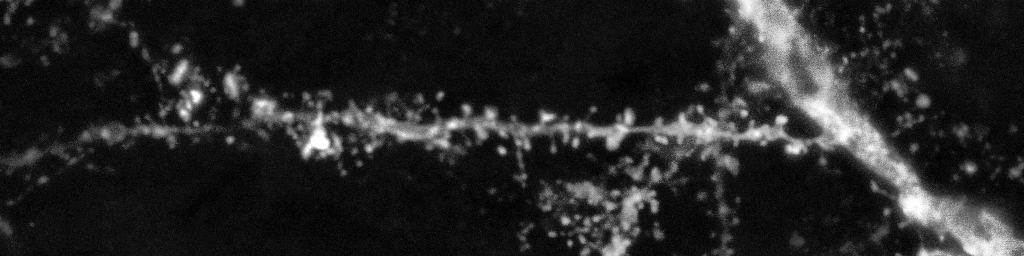

Supplement: Supplementary file 16 — Source data Fig. 7 [file 44319_2025_644_MOESM16_ESM.zip › Figure 7/7D/PFR/exp080316/MAX_PFR- Series015.tif]

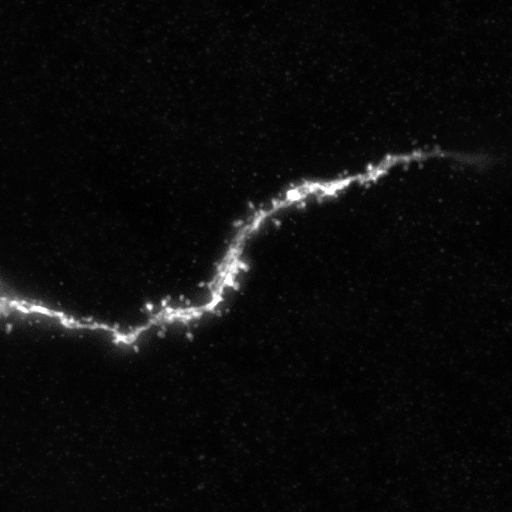

Supplement: Supplementary file 16 — Source data Fig. 7 [file 44319_2025_644_MOESM16_ESM.zip › Figure 7/7D/PFR/exp280715mount310715/MAX_PFR - 7_3.tif]

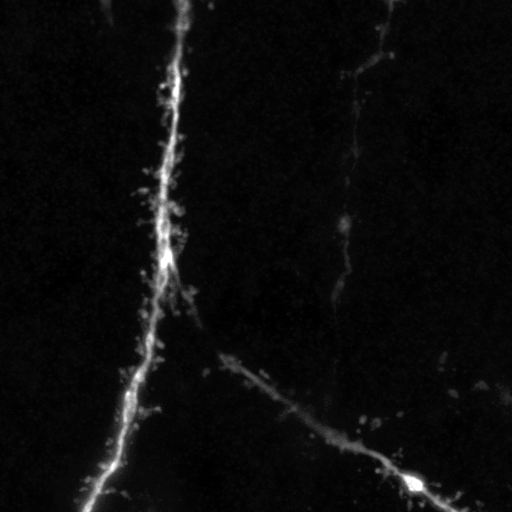

Supplement: Supplementary file 16 — Source data Fig. 7 [file 44319_2025_644_MOESM16_ESM.zip › Figure 7/7D/PFR/exp280715mount310715/MAX_PFR - slice2_4.tif]

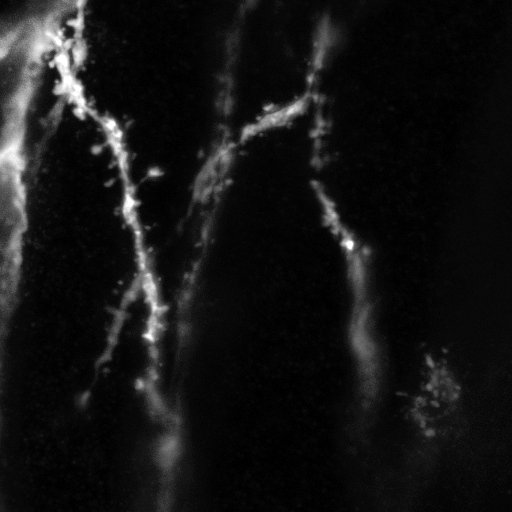

Supplement: Supplementary file 16 — Source data Fig. 7 [file 44319_2025_644_MOESM16_ESM.zip › Figure 7/7D/PFR/exp280715mount310715/MAX_PFR - slice3_4.tif]

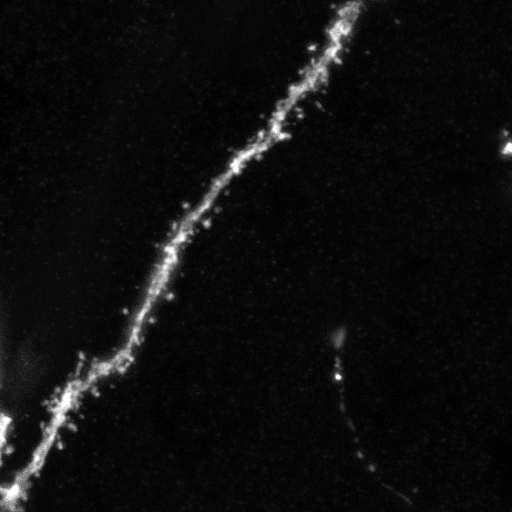

Supplement: Supplementary file 16 — Source data Fig. 7 [file 44319_2025_644_MOESM16_ESM.zip › Figure 7/7D/PFR/exp280715mount310715/MAX_PFR - slice6_1.tif]

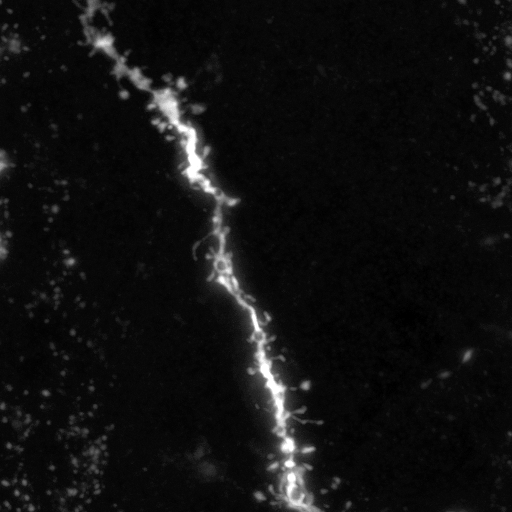

Supplement: Supplementary file 16 — Source data Fig. 7 [file 44319_2025_644_MOESM16_ESM.zip › Figure 7/7D/PFR/exp280715mount310715/MAX_PFR- slice2_3.tif]

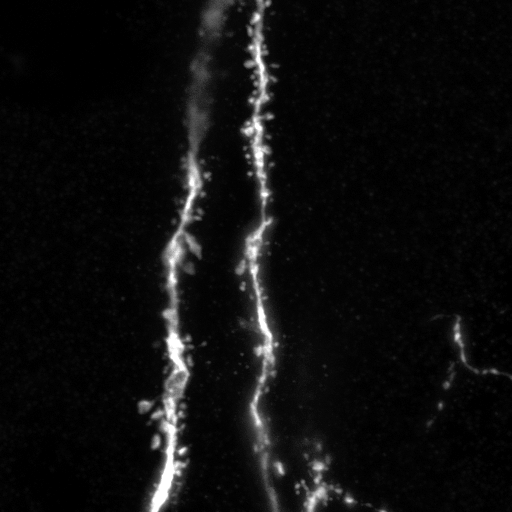

Supplement: Supplementary file 16 — Source data Fig. 7 [file 44319_2025_644_MOESM16_ESM.zip › Figure 7/7D/PFR/exp280715mount310715/MAX_PFR- slice3_1.tif]

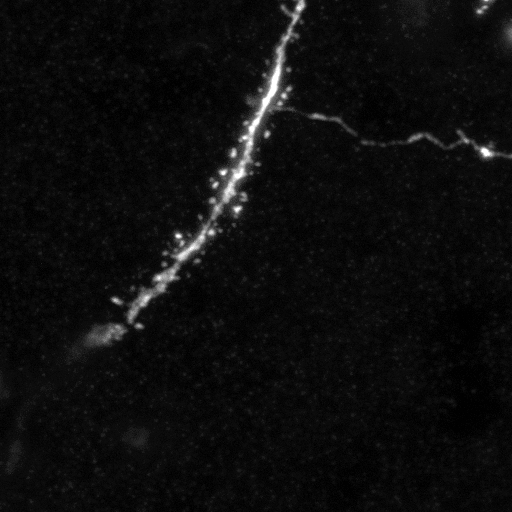

Supplement: Supplementary file 16 — Source data Fig. 7 [file 44319_2025_644_MOESM16_ESM.zip › Figure 7/7D/PFR/exp280715mount310715/MAX_PFR- slice6_2.tif]

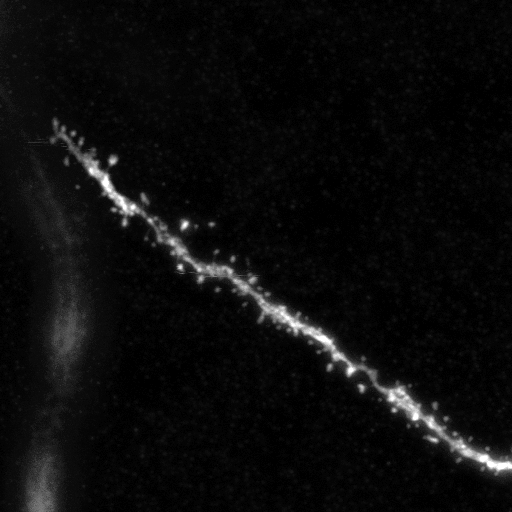

Supplement: Supplementary file 16 — Source data Fig. 7 [file 44319_2025_644_MOESM16_ESM.zip › Figure 7/7D/PFR/exp280715mount310715/MAX_PFR.lif - 7_5.tif]

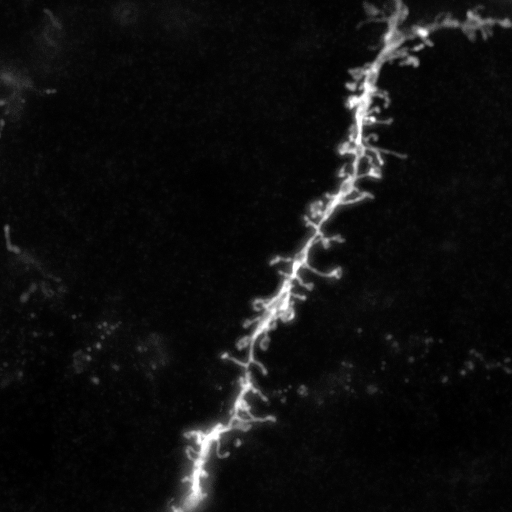

Supplement: Supplementary file 16 — Source data Fig. 7 [file 44319_2025_644_MOESM16_ESM.zip › Figure 7/7D/PFR/exp280715mount310715/PFR- slice2_1.tif]

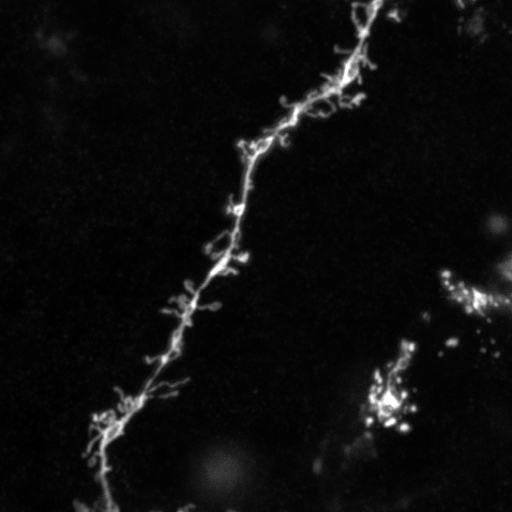

Supplement: Supplementary file 16 — Source data Fig. 7 [file 44319_2025_644_MOESM16_ESM.zip › Figure 7/7D/PFR/exp280715mount310715/PFR- slice2_2.tif]

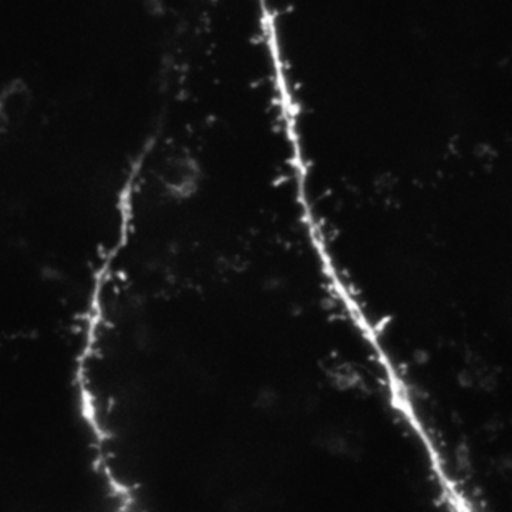

Supplement: Supplementary file 16 — Source data Fig. 7 [file 44319_2025_644_MOESM16_ESM.zip › Figure 7/7D/PFR/New folder (2)/MAX_pfr_23_5_13Series016-1.tif]

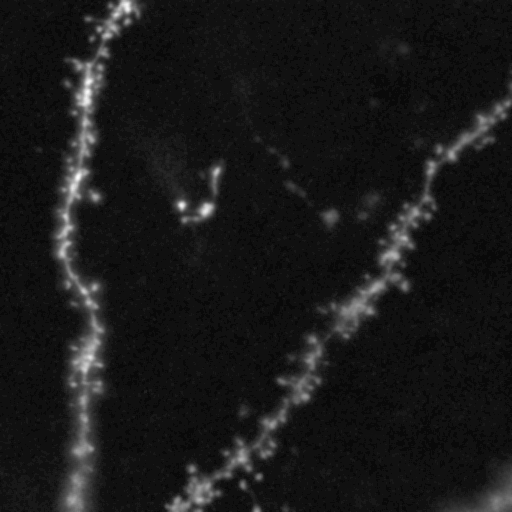

Supplement: Supplementary file 16 — Source data Fig. 7 [file 44319_2025_644_MOESM16_ESM.zip › Figure 7/7D/PFR/New folder (2)/MAX_pfr_23_5_13_ex- Series023.tif]

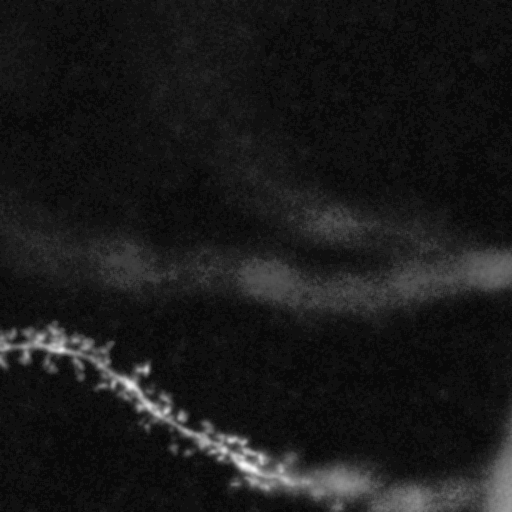

Supplement: Supplementary file 16 — Source data Fig. 7 [file 44319_2025_644_MOESM16_ESM.zip › Figure 7/7D/PFR/New folder (2)/MAX_pfr_23_5_13_exp Series009.tif]

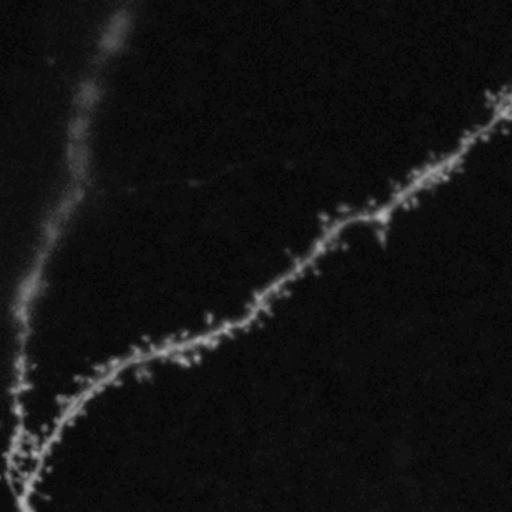

Supplement: Supplementary file 16 — Source data Fig. 7 [file 44319_2025_644_MOESM16_ESM.zip › Figure 7/7D/PFR/New folder (2)/MAX_pfr_23_5_13_exp-Series002.tif]

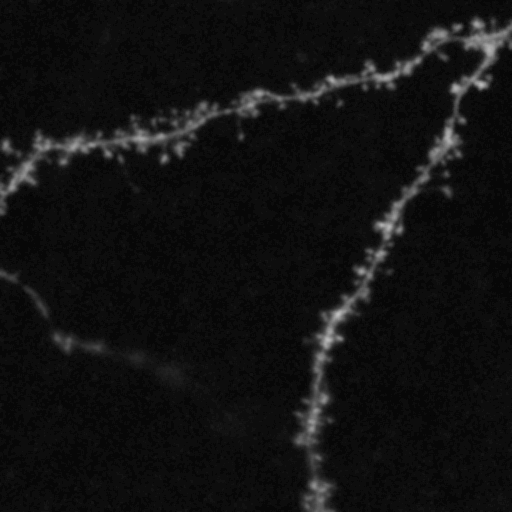

Supplement: Supplementary file 16 — Source data Fig. 7 [file 44319_2025_644_MOESM16_ESM.zip › Figure 7/7D/PFR/New folder (2)/MAX_pfr_23_5_13_exp-Series007.tif]

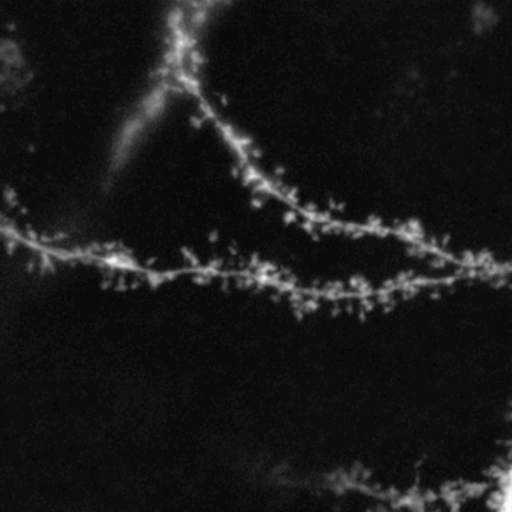

Supplement: Supplementary file 16 — Source data Fig. 7 [file 44319_2025_644_MOESM16_ESM.zip › Figure 7/7D/PFR/New folder (2)/MAX_pfr_23_5_13_exp-Series009-1.tif]

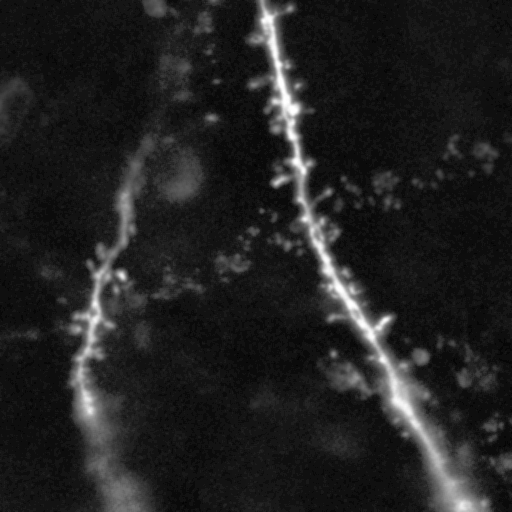

Supplement: Supplementary file 16 — Source data Fig. 7 [file 44319_2025_644_MOESM16_ESM.zip › Figure 7/7D/PFR/New folder (2)/MAX_pfr_23_5_13_exp-Series016.tif]

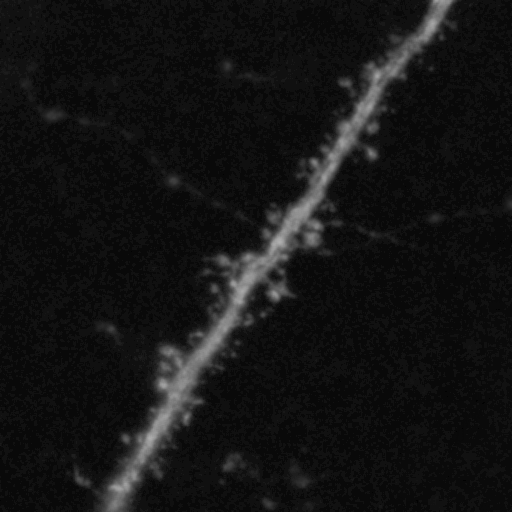

Supplement: Supplementary file 16 — Source data Fig. 7 [file 44319_2025_644_MOESM16_ESM.zip › Figure 7/7D/PFR/New folder (2)/MAX_pfr_23_5_13_exp-Series021.tif]

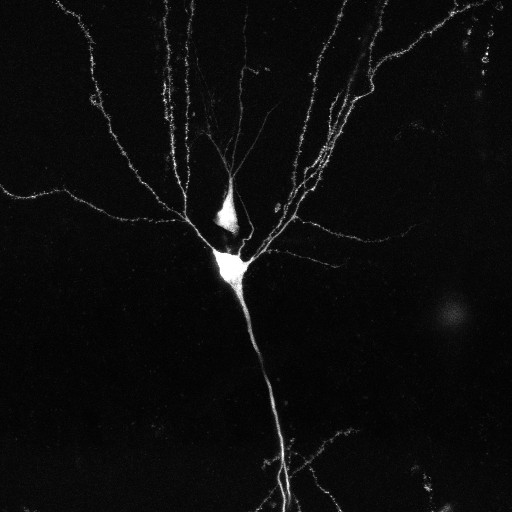

Supplement: Supplementary file 16 — Source data Fig. 7 [file 44319_2025_644_MOESM16_ESM.zip › Figure 7/7D/PFR/New folder (2)/MAX_pfr_23_5_13_exp.lif - Series060.tif]

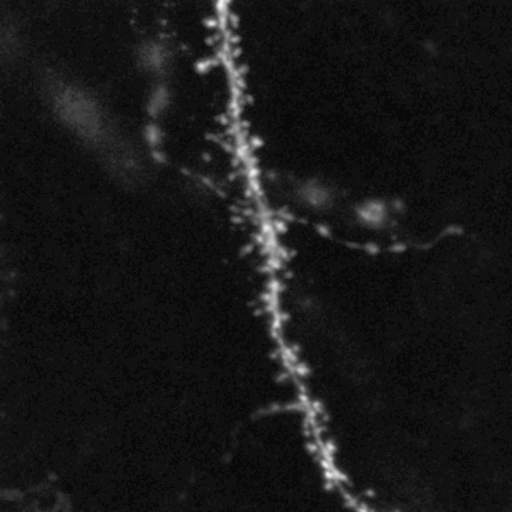

Supplement: Supplementary file 16 — Source data Fig. 7 [file 44319_2025_644_MOESM16_ESM.zip › Figure 7/7D/PFR/New folder (2)/MAX_pfr_23_5_13_exSeries012.tif]

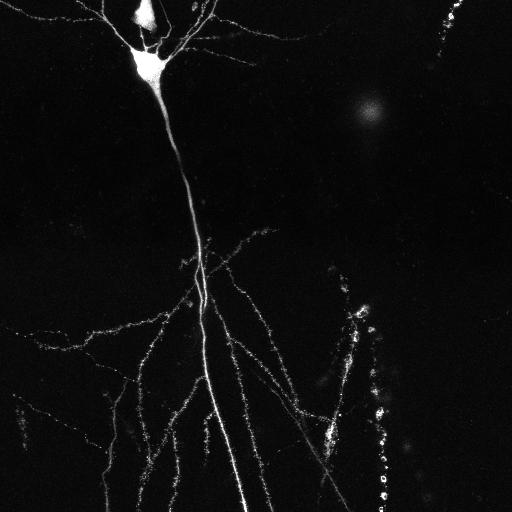

Supplement: Supplementary file 16 — Source data Fig. 7 [file 44319_2025_644_MOESM16_ESM.zip › Figure 7/7D/PFR/New folder (2)/neuron.tif]

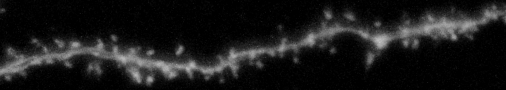

Supplement: Supplementary file 16 — Source data Fig. 7 [file 44319_2025_644_MOESM16_ESM.zip › Figure 7/7D/PFR/New folder (2)/PFR -series 002.tif]

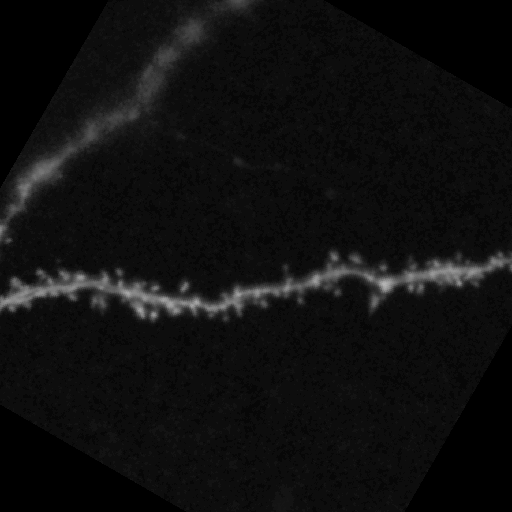

Supplement: Supplementary file 16 — Source data Fig. 7 [file 44319_2025_644_MOESM16_ESM.zip › Figure 7/7D/PFR/New folder (2)/PFR-Series002.tif]

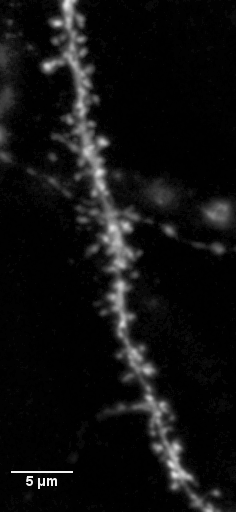

Supplement: Supplementary file 16 — Source data Fig. 7 [file 44319_2025_644_MOESM16_ESM.zip › Figure 7/7D/PFR/New folder (2)/PFR.tif]

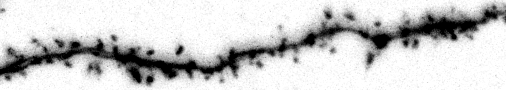

Supplement: Supplementary file 16 — Source data Fig. 7 [file 44319_2025_644_MOESM16_ESM.zip › Figure 7/7D/PFR/PFR -series 002 final.tif]

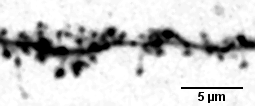

Supplement: Supplementary file 16 — Source data Fig. 7 [file 44319_2025_644_MOESM16_ESM.zip › Figure 7/7D/PFR/PFR final-zoom.tif]

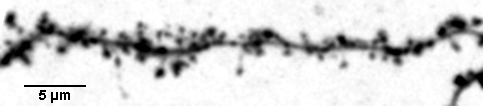

Supplement: Supplementary file 16 — Source data Fig. 7 [file 44319_2025_644_MOESM16_ESM.zip › Figure 7/7D/PFR/PFR final.tif]

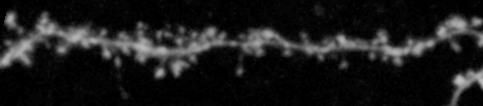

Supplement: Supplementary file 16 — Source data Fig. 7 [file 44319_2025_644_MOESM16_ESM.zip › Figure 7/7D/PFR/PFR-Series007cut.tif]

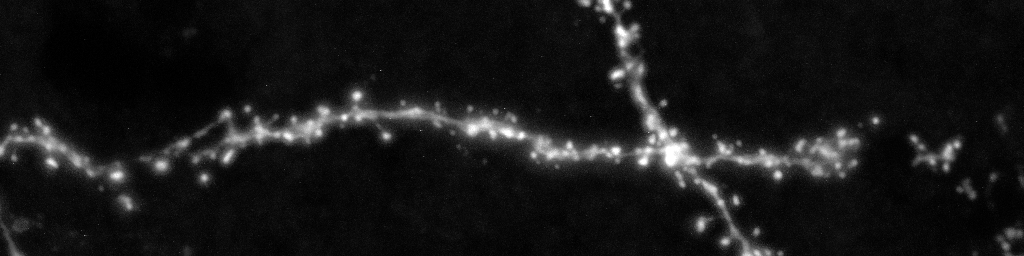

Supplement: Supplementary file 16 — Source data Fig. 7 [file 44319_2025_644_MOESM16_ESM.zip › Figure 7/7D/Timp3+PFR/exp020216/MAX_PFR+Timp3 - Series045.tif]

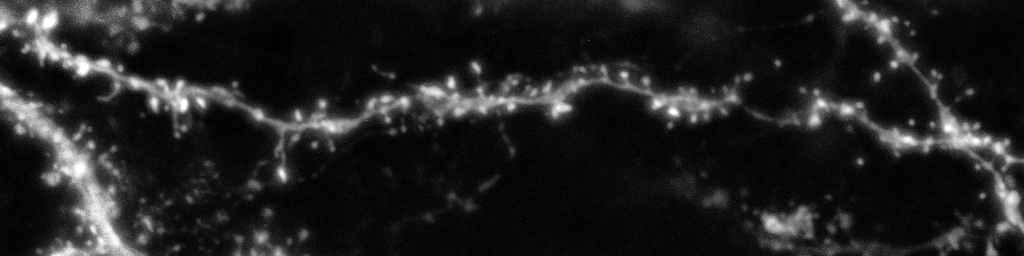

Supplement: Supplementary file 16 — Source data Fig. 7 [file 44319_2025_644_MOESM16_ESM.zip › Figure 7/7D/Timp3+PFR/exp020216/MAX_PFR+Timp3 - Series059.tif]

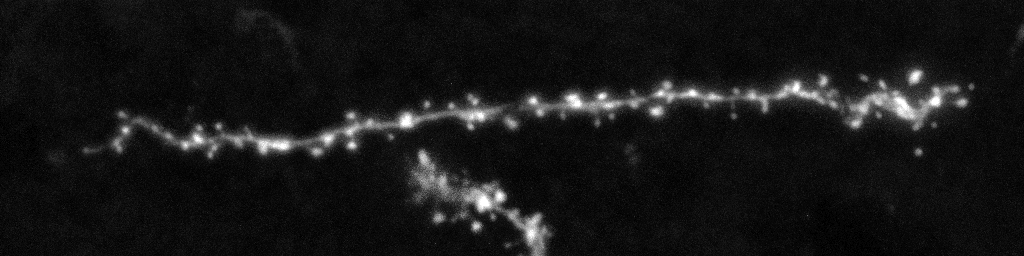

Supplement: Supplementary file 16 — Source data Fig. 7 [file 44319_2025_644_MOESM16_ESM.zip › Figure 7/7D/Timp3+PFR/exp020216/MAX_PFR+Timp3- Series009.tif]

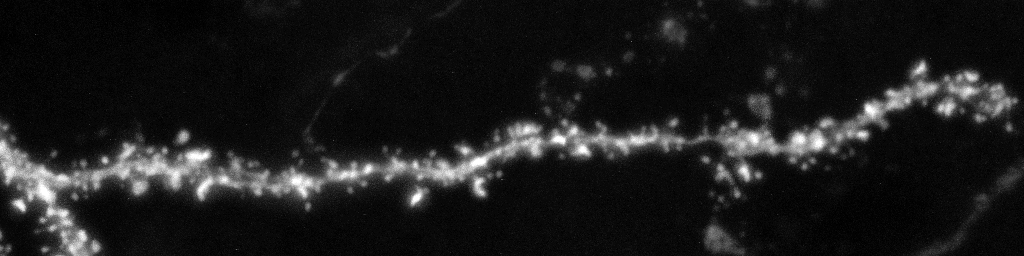

Supplement: Supplementary file 16 — Source data Fig. 7 [file 44319_2025_644_MOESM16_ESM.zip › Figure 7/7D/Timp3+PFR/exp020216/MAX_PFR+Timp3- Series011.tif]

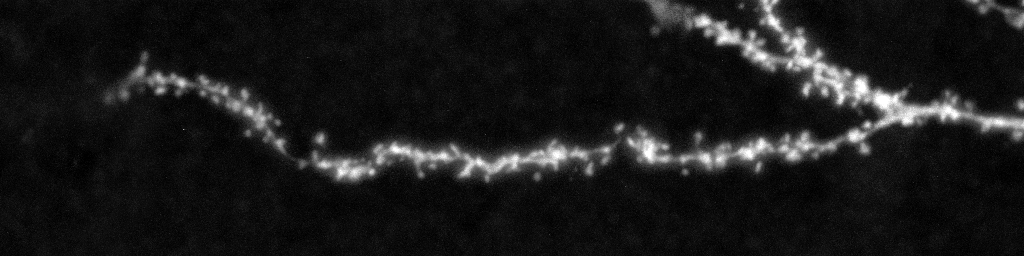

Supplement: Supplementary file 16 — Source data Fig. 7 [file 44319_2025_644_MOESM16_ESM.zip › Figure 7/7D/Timp3+PFR/exp020216/MAX_PFR+Timp3- Series022.tif]

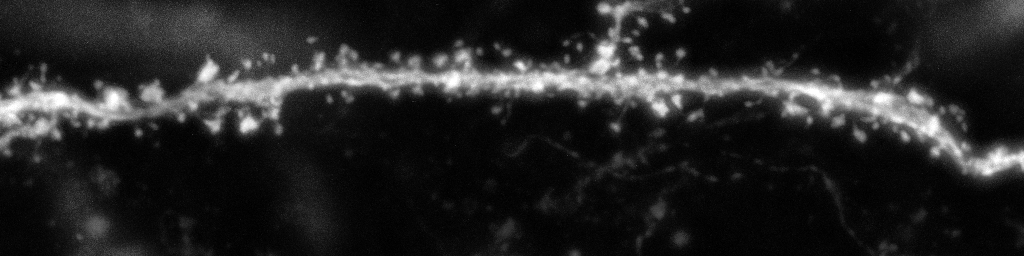

Supplement: Supplementary file 16 — Source data Fig. 7 [file 44319_2025_644_MOESM16_ESM.zip › Figure 7/7D/Timp3+PFR/exp020216/MAX_PFR+Timp3- Series025.tif]

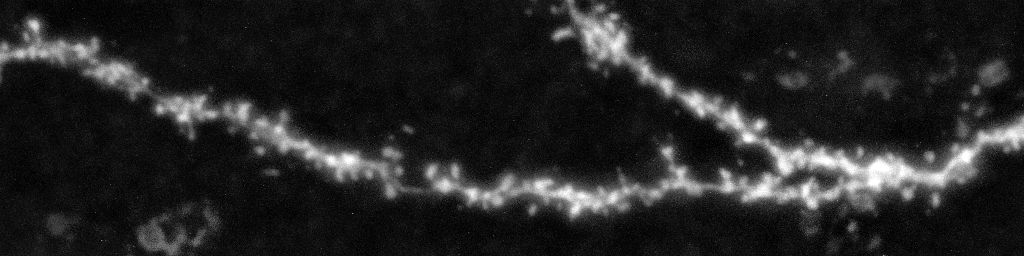

Supplement: Supplementary file 16 — Source data Fig. 7 [file 44319_2025_644_MOESM16_ESM.zip › Figure 7/7D/Timp3+PFR/exp020216/MAX_PFR+Timp3- Series030.tif]

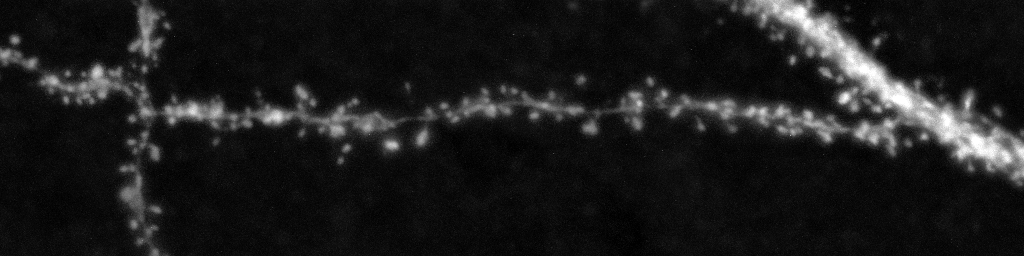

Supplement: Supplementary file 16 — Source data Fig. 7 [file 44319_2025_644_MOESM16_ESM.zip › Figure 7/7D/Timp3+PFR/exp020216/MAX_PFR+Timp3- Series034.tif]

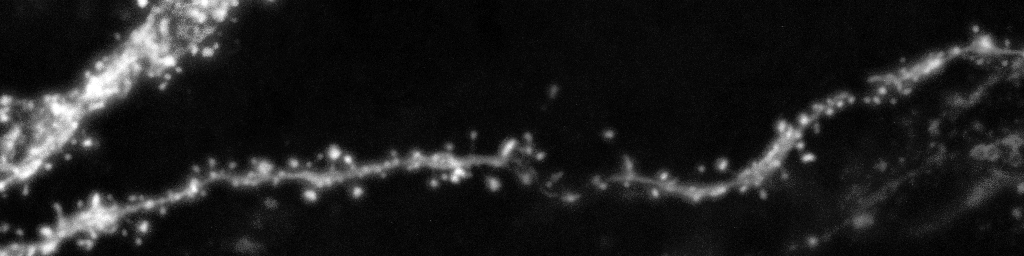

Supplement: Supplementary file 16 — Source data Fig. 7 [file 44319_2025_644_MOESM16_ESM.zip › Figure 7/7D/Timp3+PFR/exp020216/MAX_PFR+Timp3- Series035.tif]

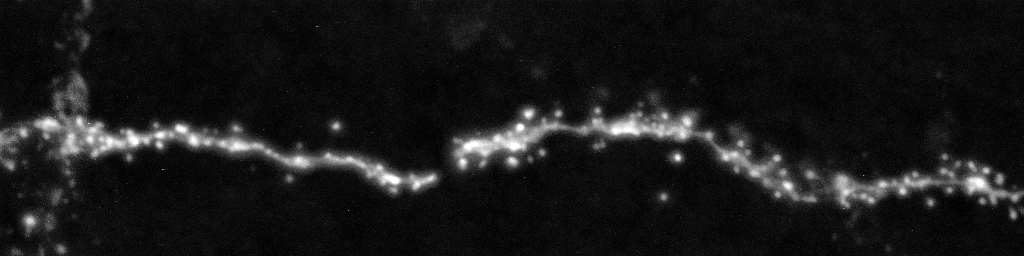

Supplement: Supplementary file 16 — Source data Fig. 7 [file 44319_2025_644_MOESM16_ESM.zip › Figure 7/7D/Timp3+PFR/exp020216/MAX_PFR+Timp3- Series050-1.tif]

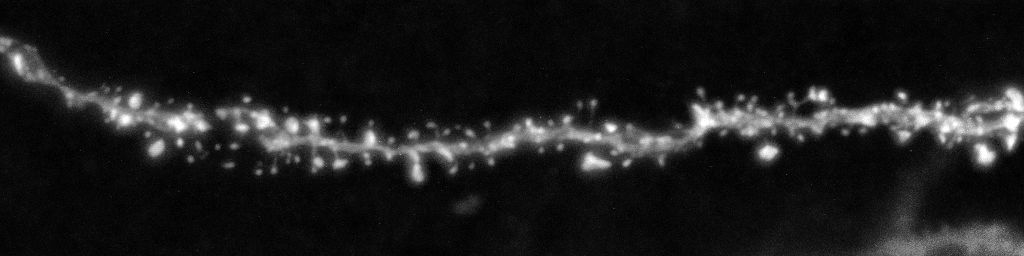

Supplement: Supplementary file 16 — Source data Fig. 7 [file 44319_2025_644_MOESM16_ESM.zip › Figure 7/7D/Timp3+PFR/exp020216/MAX_PFR+Timp3- Series054.tif]

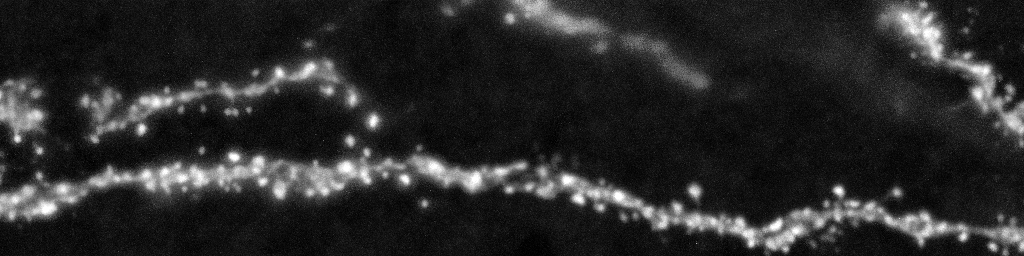

Supplement: Supplementary file 16 — Source data Fig. 7 [file 44319_2025_644_MOESM16_ESM.zip › Figure 7/7D/Timp3+PFR/exp020216/MAX_PFR+Timp3- Series071.tif]

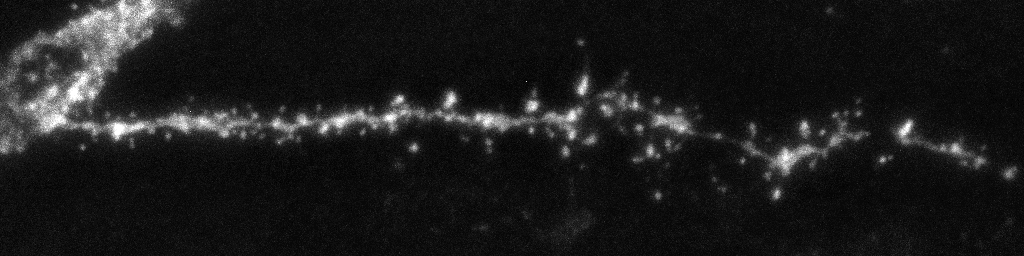

Supplement: Supplementary file 16 — Source data Fig. 7 [file 44319_2025_644_MOESM16_ESM.zip › Figure 7/7D/Timp3+PFR/exp140316/MAX_ctl - Series021.tif]

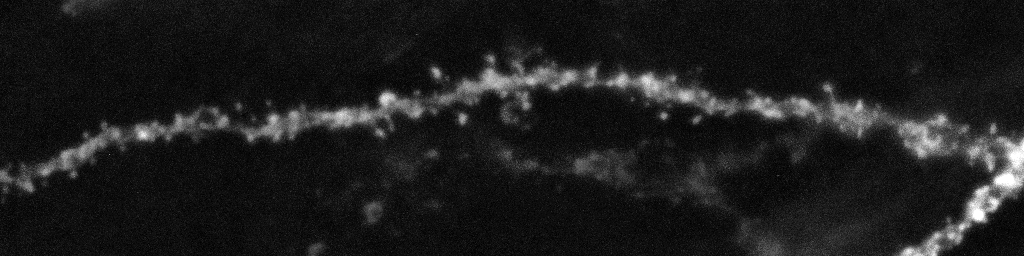

Supplement: Supplementary file 16 — Source data Fig. 7 [file 44319_2025_644_MOESM16_ESM.zip › Figure 7/7D/Timp3+PFR/exp140316/MAX_PFR+TIMP3 - Series007.tif]

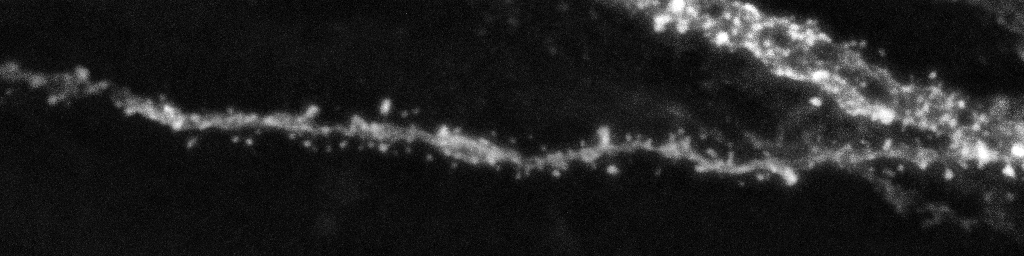

Supplement: Supplementary file 16 — Source data Fig. 7 [file 44319_2025_644_MOESM16_ESM.zip › Figure 7/7D/Timp3+PFR/exp140316/MAX_PFR+TIMP3- Series004.tif]

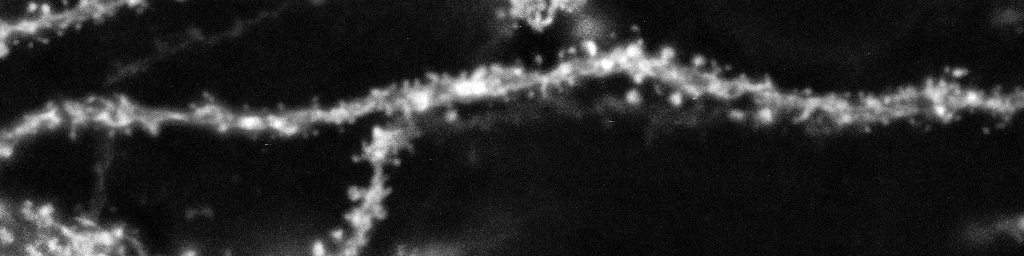

Supplement: Supplementary file 16 — Source data Fig. 7 [file 44319_2025_644_MOESM16_ESM.zip › Figure 7/7D/Timp3+PFR/exp140316/MAX_PFR+TIMP3- Series019.tif]

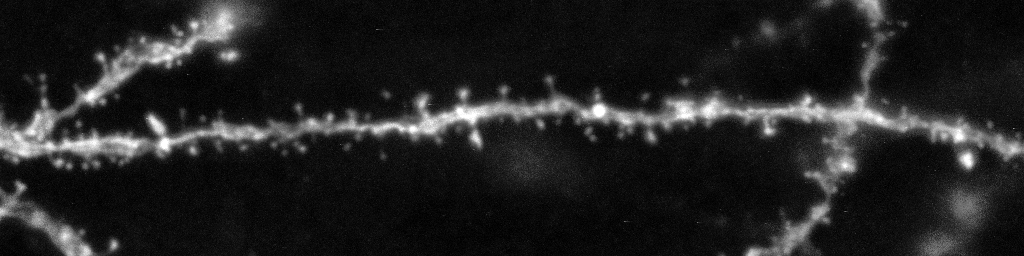

Supplement: Supplementary file 16 — Source data Fig. 7 [file 44319_2025_644_MOESM16_ESM.zip › Figure 7/7D/Timp3+PFR/exp140316/MAX_PFR+TIMP3- Series027.tif]

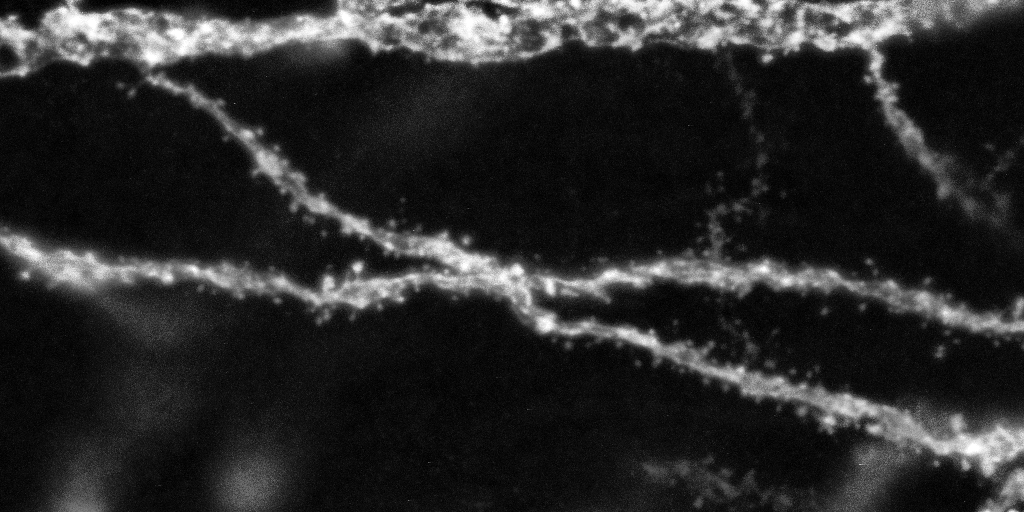

Supplement: Supplementary file 16 — Source data Fig. 7 [file 44319_2025_644_MOESM16_ESM.zip › Figure 7/7D/Timp3+PFR/exp140316/MAX_PFR+TIMP3- Series033.tif]

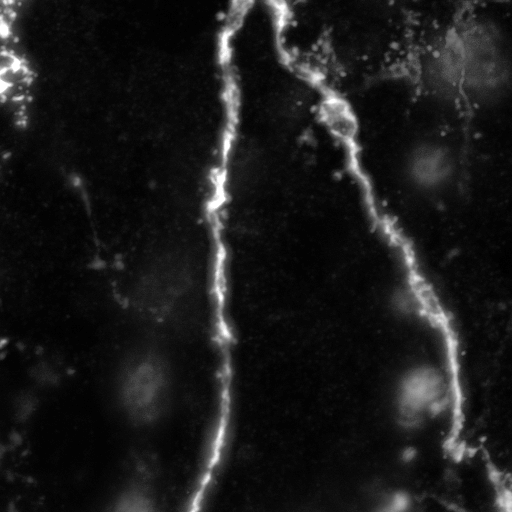

Supplement: Supplementary file 16 — Source data Fig. 7 [file 44319_2025_644_MOESM16_ESM.zip › Figure 7/7D/Timp3+PFR/exp230615/MAX_Timp3 - slice1 n6.tif]

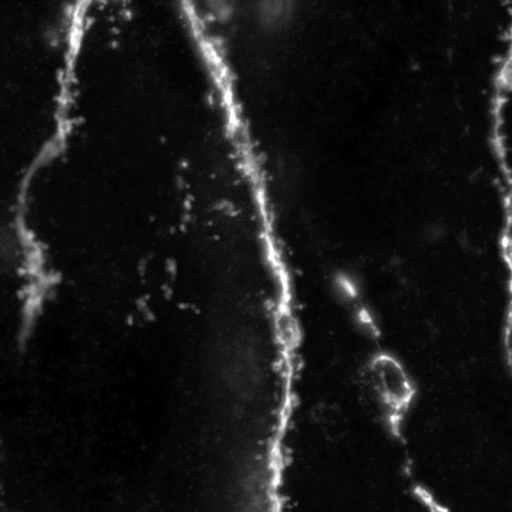

Supplement: Supplementary file 16 — Source data Fig. 7 [file 44319_2025_644_MOESM16_ESM.zip › Figure 7/7D/Timp3+PFR/exp230615/MAX_Timp3- slice1 n2.tif]

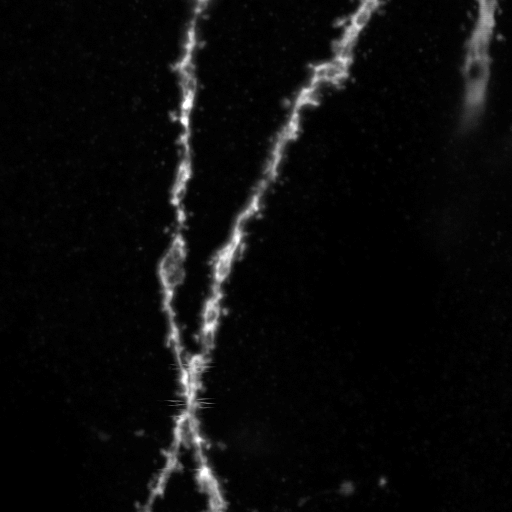

Supplement: Supplementary file 16 — Source data Fig. 7 [file 44319_2025_644_MOESM16_ESM.zip › Figure 7/7D/Timp3+PFR/exp230615/MAX_Timp3i - slice4 n2.tif]

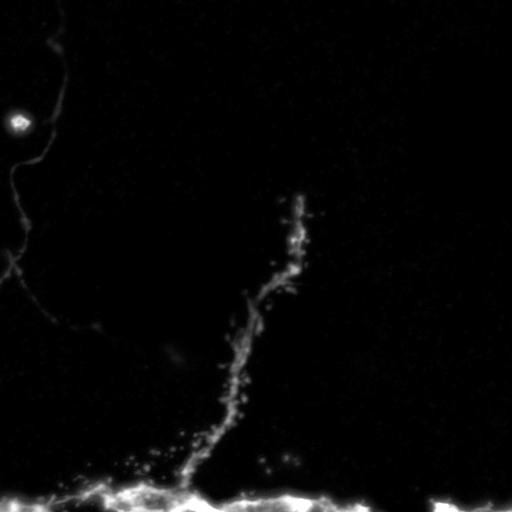

Supplement: Supplementary file 16 — Source data Fig. 7 [file 44319_2025_644_MOESM16_ESM.zip › Figure 7/7D/Timp3+PFR/exp230615/MAX_Timp3i- slice2 n3.tif]

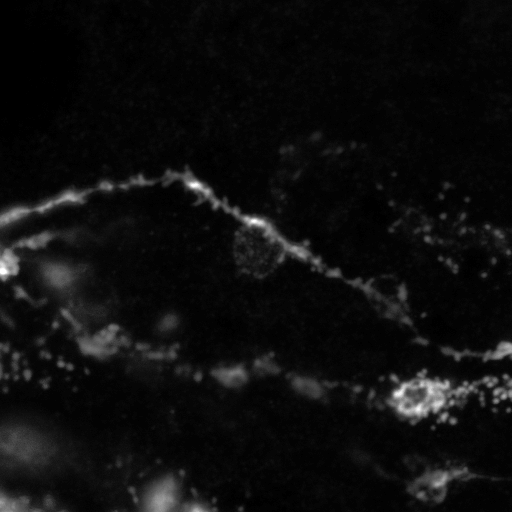

Supplement: Supplementary file 16 — Source data Fig. 7 [file 44319_2025_644_MOESM16_ESM.zip › Figure 7/7D/Timp3+PFR/exp230615/MAX_Timp3i- slice3 n1.tif]

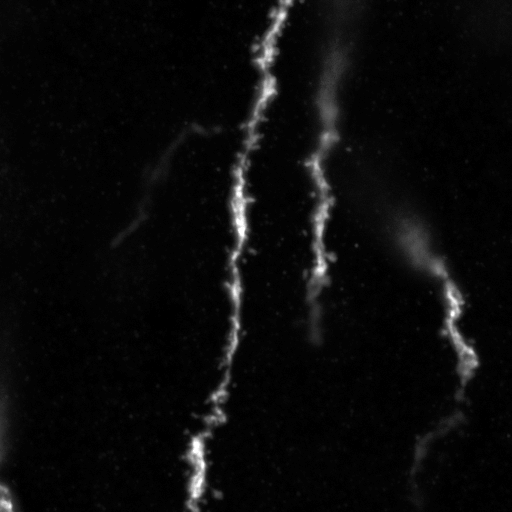

Supplement: Supplementary file 16 — Source data Fig. 7 [file 44319_2025_644_MOESM16_ESM.zip › Figure 7/7D/Timp3+PFR/exp230615/MAX_Timp3i- slice4 n3.tif]

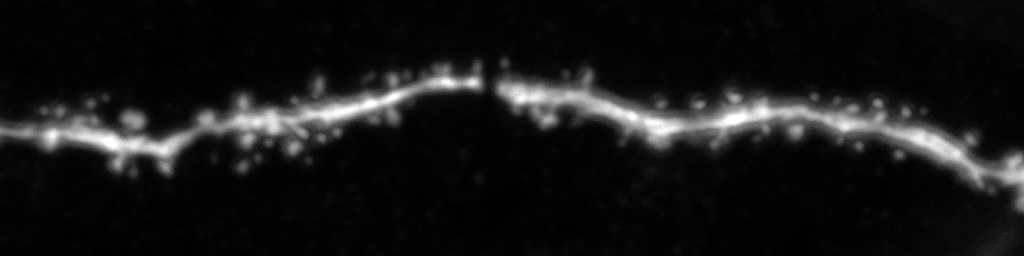

Supplement: Supplementary file 16 — Source data Fig. 7 [file 44319_2025_644_MOESM16_ESM.zip › Figure 7/7D/Timp3+PFR/exp280715mount310715/Timp3 - 2_4.tif]

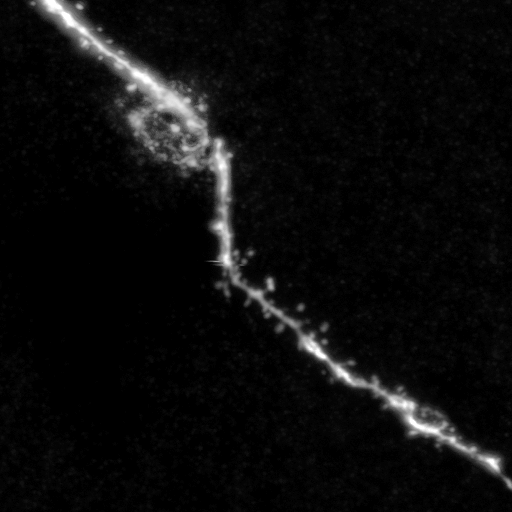

Supplement: Supplementary file 16 — Source data Fig. 7 [file 44319_2025_644_MOESM16_ESM.zip › Figure 7/7D/Timp3+PFR/exp280715mount310715/Timp3 - 3_3.tif]

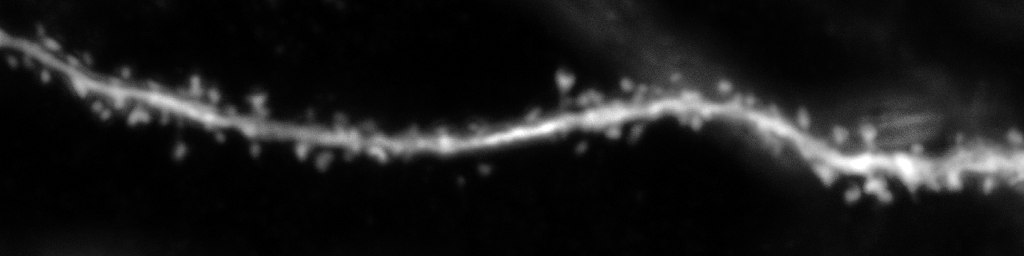

Supplement: Supplementary file 16 — Source data Fig. 7 [file 44319_2025_644_MOESM16_ESM.zip › Figure 7/7D/Timp3+PFR/exp280715mount310715/Timp3- 2_1.tif]

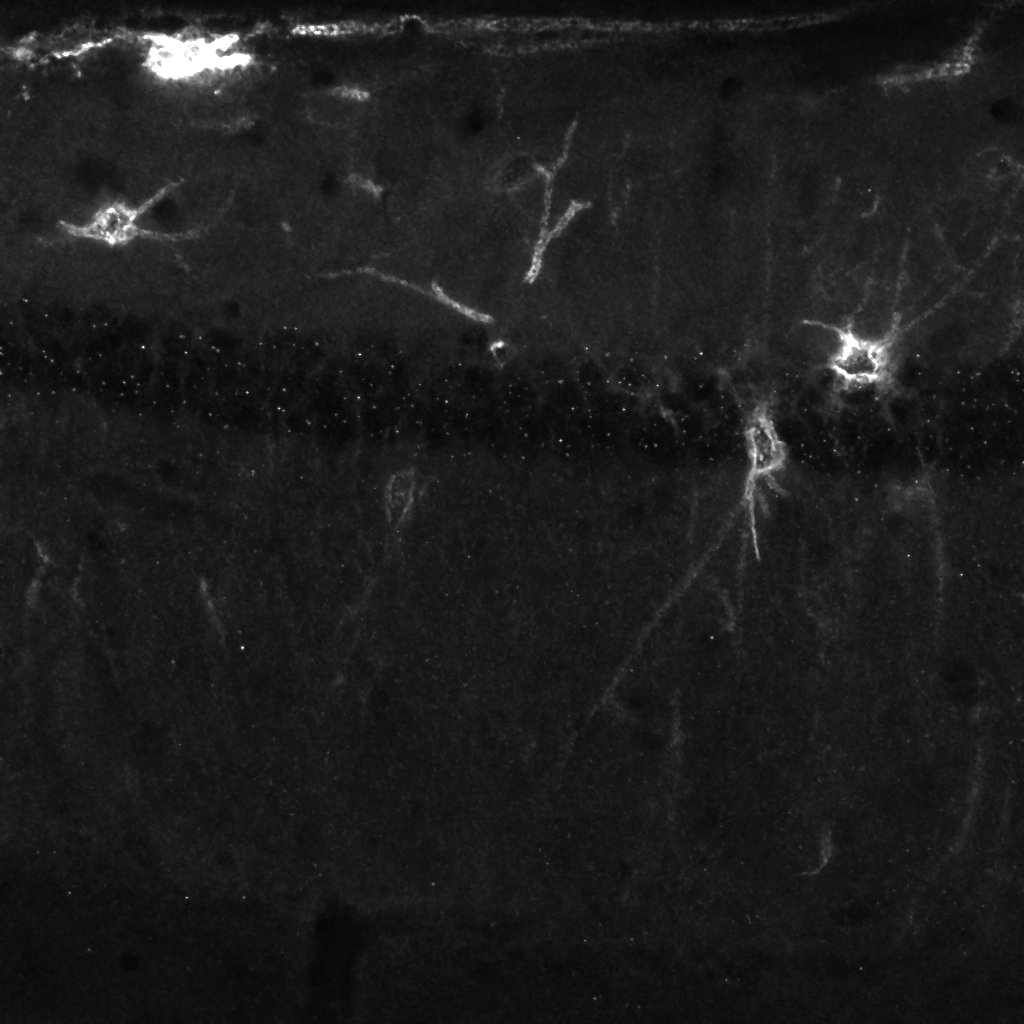

Supplement: Supplementary file 16 — Source data Fig. 7 [file 44319_2025_644_MOESM16_ESM.zip › Figure 7/Figure 7B agg.tif]

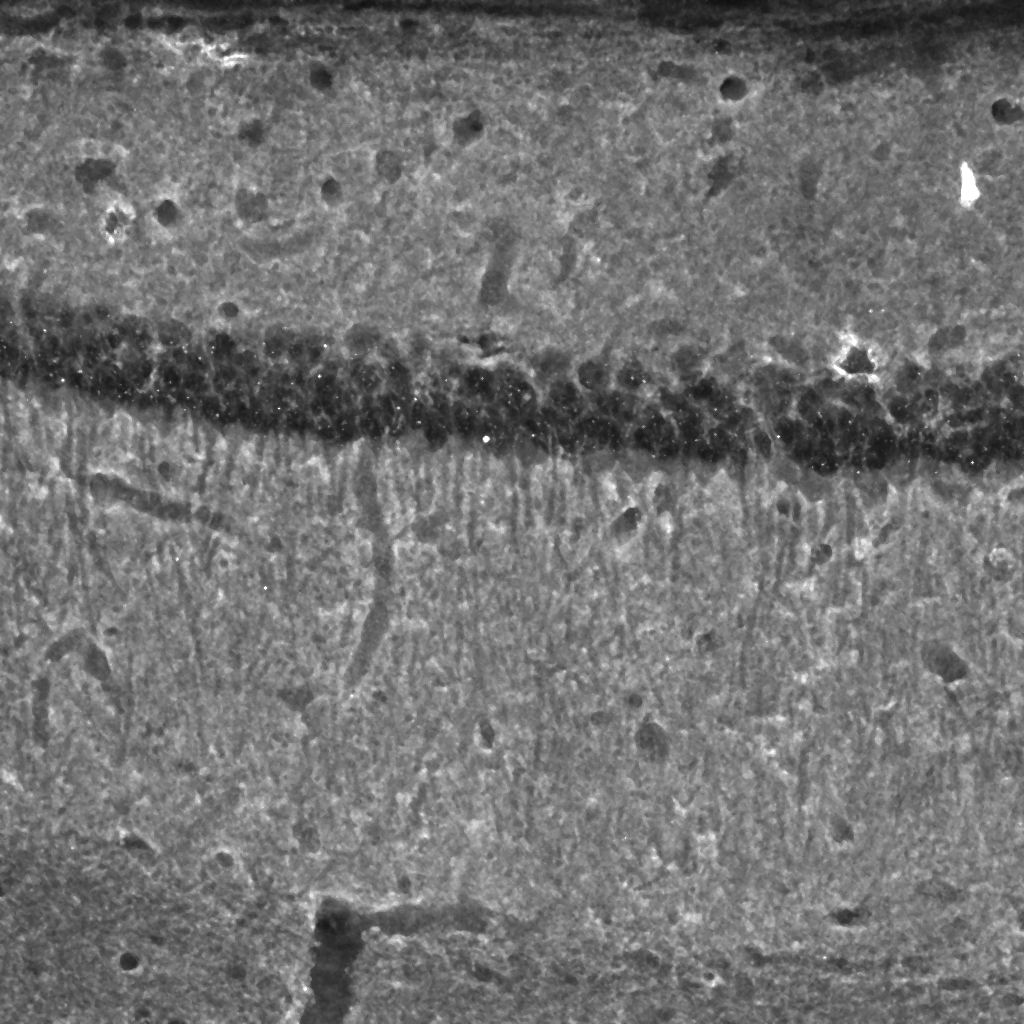

Supplement: Supplementary file 16 — Source data Fig. 7 [file 44319_2025_644_MOESM16_ESM.zip › Figure 7/Figure 7B BC.tif]

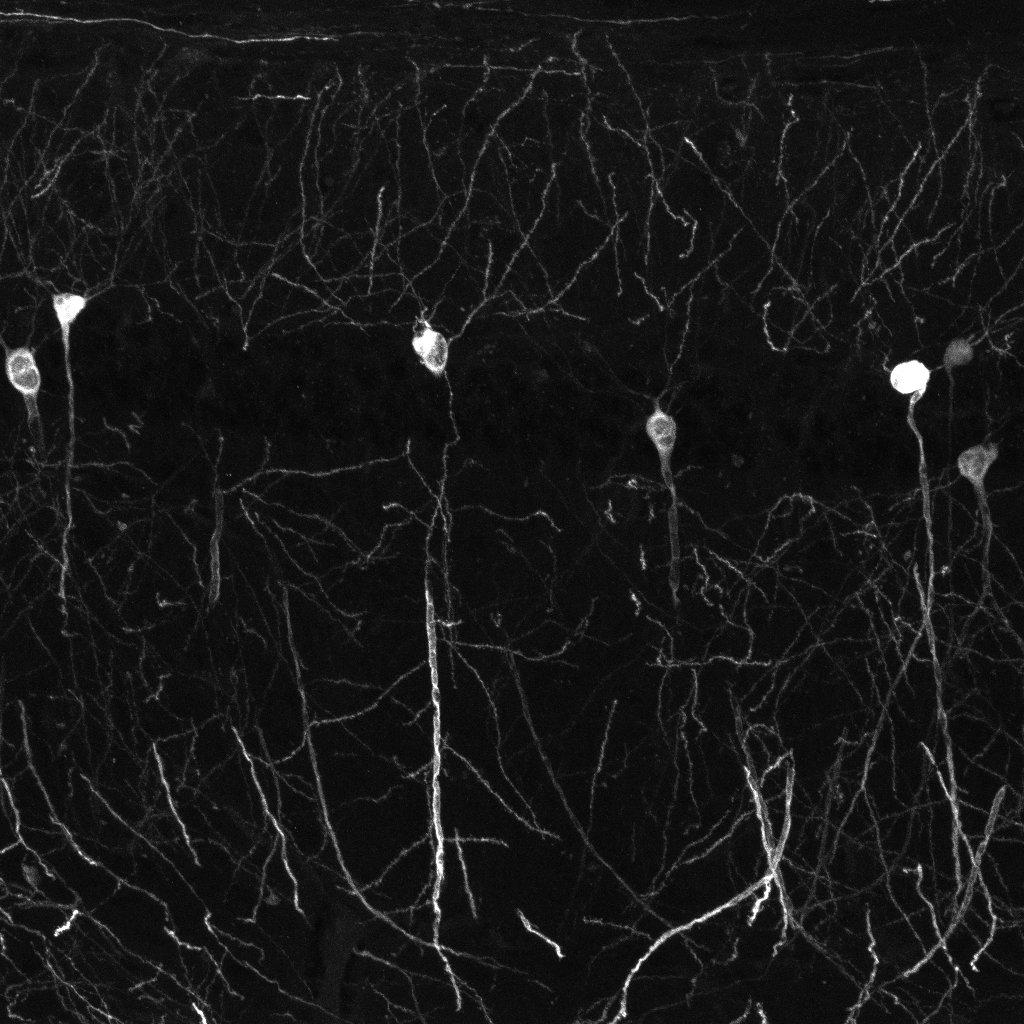

Supplement: Supplementary file 16 — Source data Fig. 7 [file 44319_2025_644_MOESM16_ESM.zip › Figure 7/Figure 7B YFP.tif]

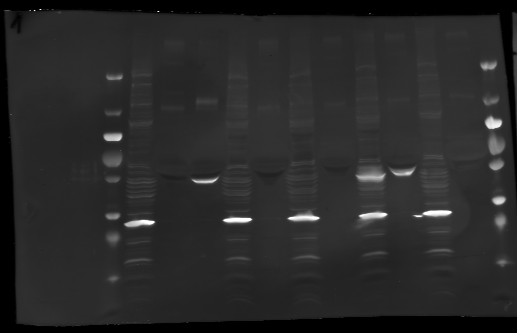

Supplement: Supplementary file 17 — Figure EV3 Source Data [file 44319_2025_644_MOESM17_ESM.zip › Figure EV3/Extended view 3B Rb Neo-epitope.tiff]

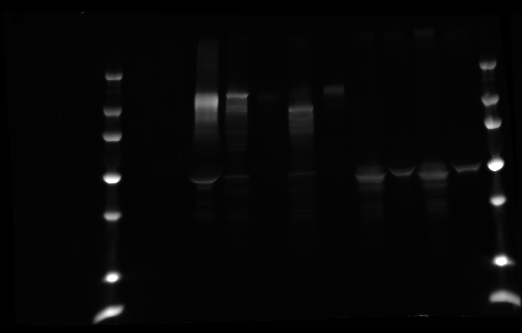

Supplement: Supplementary file 17 — Figure EV3 Source Data [file 44319_2025_644_MOESM17_ESM.zip › Figure EV3/Extended View 3B.tif]

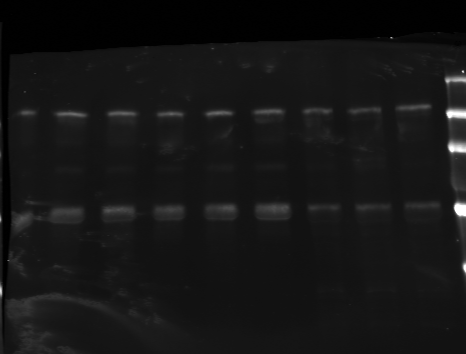

Supplement: Supplementary file 18 — Figure EV4 Source Data [file 44319_2025_644_MOESM18_ESM.zip › Figure EV4/Extended view 4 Ctl-PFR-PFR+Aniso.tif]

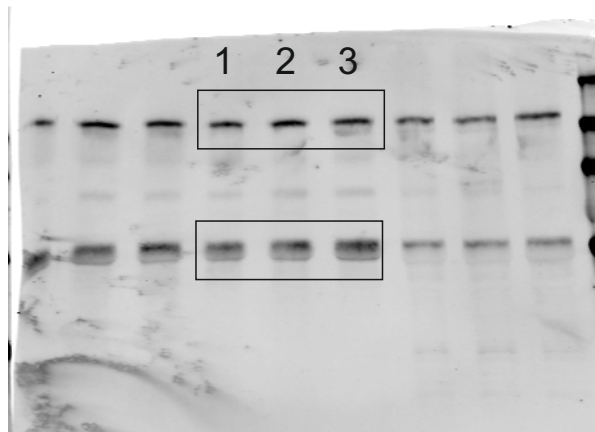

1. Ctl
2. PFR
3. PFR+Anisomycin

ms anti brevican

Supplement: Supplementary file 18 — Figure EV4 Source Data [file 44319_2025_644_MOESM18_ESM.zip › Figure EV4/Extended view 4.pdf]
