# Supplementary material for: Activity-dependent extracellular proteolytic cascade cleaves the ECM component brevican to promote structural plasticity
Source: EMBO Rep. 2025 Nov 19;27(1):163–85. doi: 10.1038/s44319-025-00644-w (PMC12796228; doi:10.1038/s44319-025-00644-w)

ms anti brevican

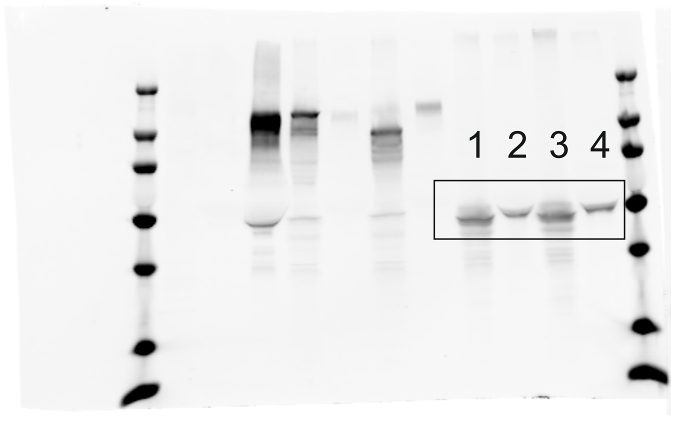

1. BCneo cell lysate
2. BCneo spernatant
3. BCneo+1 cell lysate
4. BCneo+1 supernatant

rb anti neo

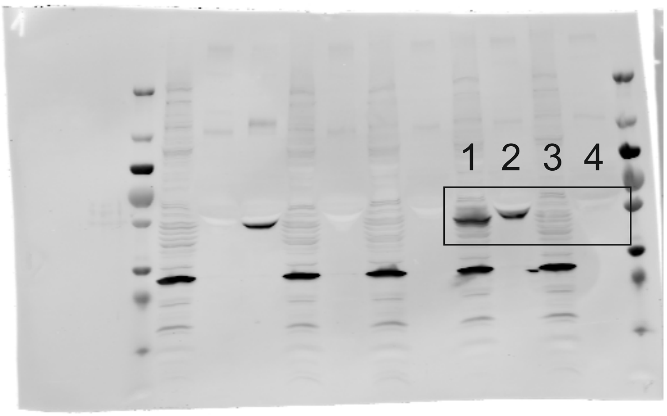

Supplement: Supplementary file 17 — Figure EV3 Source Data [file 44319_2025_644_MOESM17_ESM.zip › Figure EV3/Extended view 3B.pdf]
