# Supplementary material for: Activity-dependent extracellular proteolytic cascade cleaves the ECM component brevican to promote structural plasticity
Source: EMBO Rep. 2025 Nov 19;27(1):163–85. doi: 10.1038/s44319-025-00644-w (PMC12796228; doi:10.1038/s44319-025-00644-w)
Supplement: Supplementary file 19 — Expanded View Figures [file 44319_2025_644_MOESM19_ESM.pdf]

Expanded View Figures

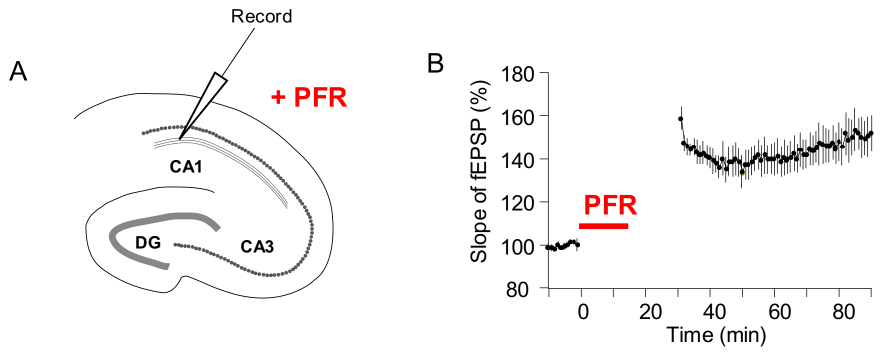

Figure EV1. Extracellular electrophysiological recordings were made in the CA1 region of an acute hippocampal slice following PFR treatment.

Note the robust and lasting increase in the slope of the field excitatory postsynaptic potential (fEPSP) 15 min after PFR treatment, which represents the induction of cLTP.

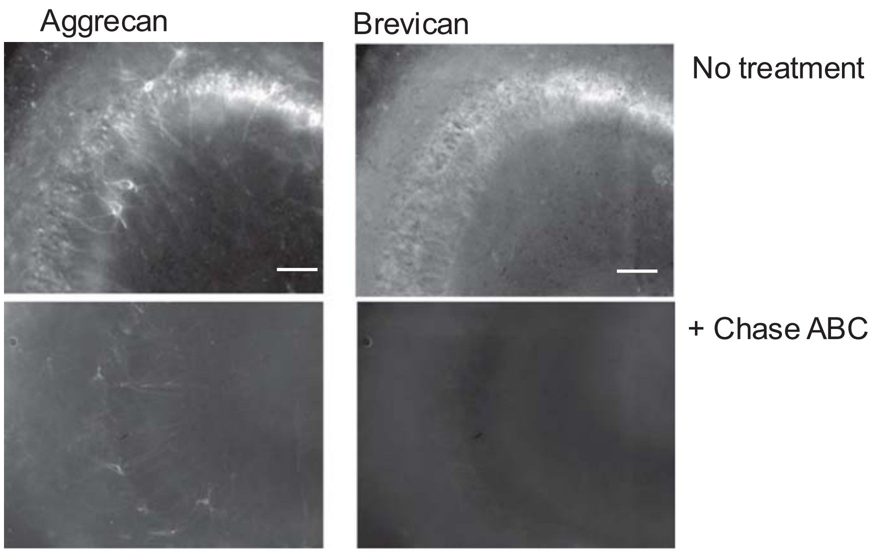

Figure EV2. Chondroitinase ABC (Chase ABC) digestion abolishes ECM staining.

Acute hippocampal slices from rats were stained with the ECM proteins aggrecan (left) and brevican (right). Incubation with chondroitinase ABC (lower panel) led to a strong reduction of aggrecan and brevican staining, indicating its solubilization (Scale bar 500 μm).

 © The Author(s)

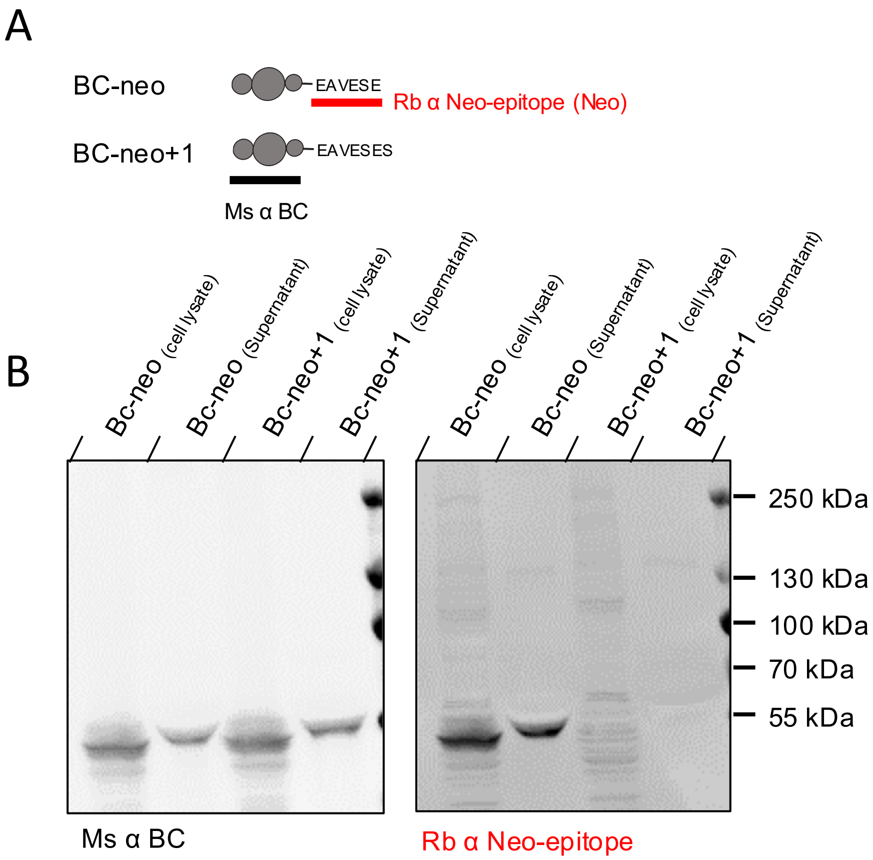

Figure EV3. Neo-Epitope antibody detects exclusively proteolytically cleaved brevican between Glu^{395}-Ser^{396}.

(**A**) Constructs overexpressed in HEK293T cells. Rat brevican construct representing the ADAMTS-derived N-terminal proteolytic fragment (BC-neo). Red underlined are the amino acids that served as epitope to generate the rb α Neo-epitope antibody. The C-terminus of BC-neo+1 includes the serine (S) that follows predicted ADAMTS cleavage site. (**B**) Western blots of Cell lysates and supernatants of HEK293T cells transfected with either BC-neo or BC-neo+1 constructs. Both constructs were detected in cell the lysate and supernatant using the Ms α BC antibody (left), which is not selective for the cleavage site. Rb α Neo antibody detected exclusively the BC-neo construct and failed to recognize BC-neo+1, confirming its high specificity for the ADAMTS-derived proteolytic fragment of brevican. Source data are available online for this figure.

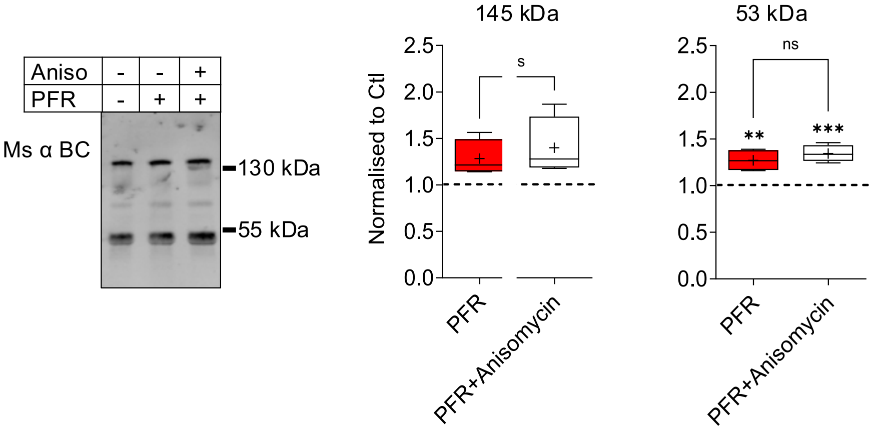

Figure EV4. The protein synthesis inhibitor anisomycin did not affect the increase in brevican levels induced by activity.

Hippocampal slices were pre-incubated with 20 μM anisomycin for 20–30 min prior to stimulation. Anisomycin had no effect on brevican full-length protein or brevican neo (53 kDa: Ctl vs. PFR: $P = 0.04$, Ctl vs. PFR+Anisomycin: $P < 0.001$. $n = 4$. One-way ANOVA, Šídák's multiple comparisons test, n.s. $P > 0.05$, *$P < 0.05$, **$P < 0.01$, ***$P < 0.001$. Box plot depicts the interquartile range (IQR, box), median is indicated as line, average as + and whiskers indicate minimal to maximal data point). For detailed statistics see Table EV8. Source data are available online for this figure.

 © The Author(s)

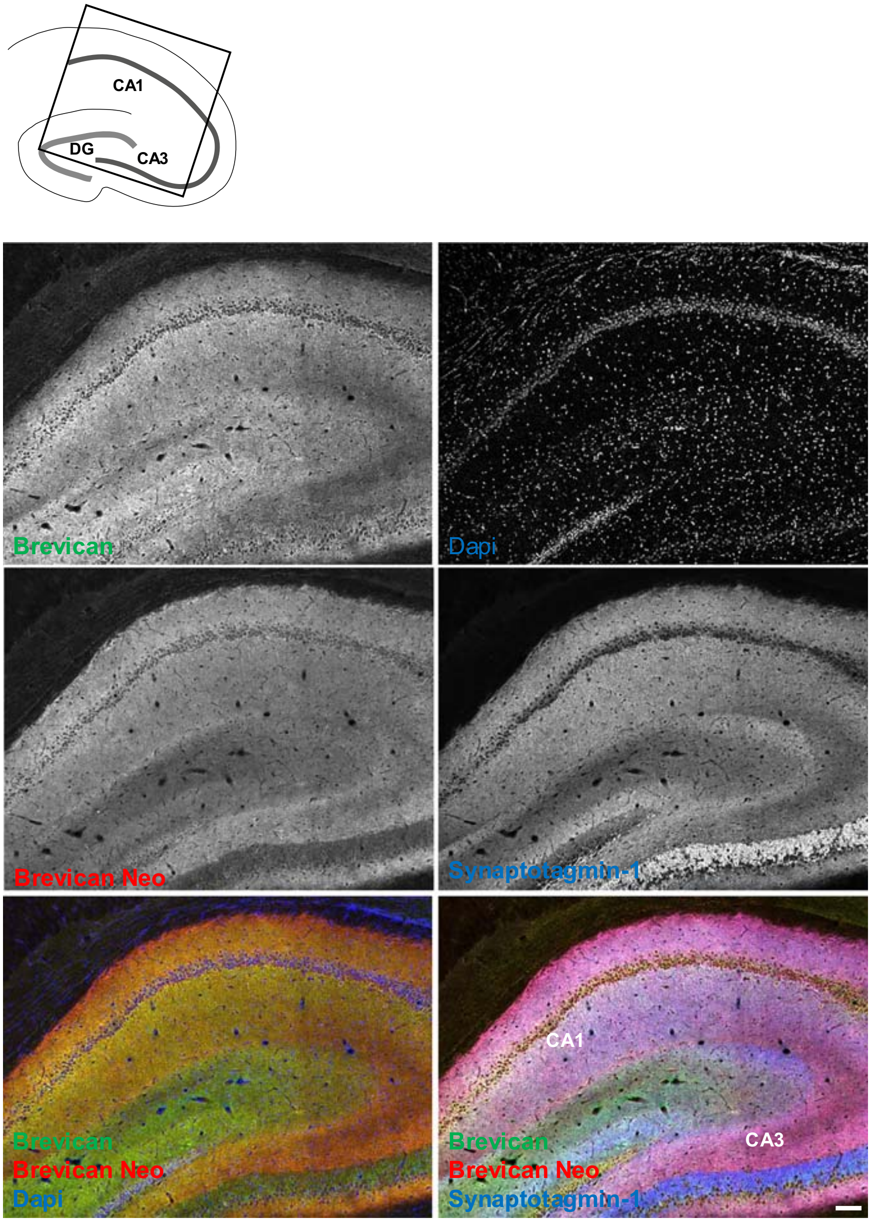

Figure EV5. Distribution of brevican and brevican neo in the hippocampus.

Cryosections of paraformaldehyde-fixed adult rat brains were stained for brevican using guinea pig anti-brevican (green; see also Valenzuela et al, 2014), brevican neo (red), synaptotagmin-1 (blue) antibody and DAPI. A tile scan of the hippocampus is depicted. Note the diffuse staining of both brevican and brevican neo throughout the hippocampus (scale bar 100 µm).
